# Identification and Development of Cyclic Peptide Inhibitors of Hypoxia Inducible Factors 1 and 2 That Disrupt Hypoxia-Response Signaling in Cancer Cells

**DOI:** 10.1021/jacs.3c10508

**Published:** 2024-03-19

**Authors:** Andrew T. Ball, Soran Mohammed, Cyrielle Doigneaux, Reece M. Gardner, James W. Easton, Steven Turner, Jonathan W. Essex, Garry Pairaudeau, Ali Tavassoli

**Affiliations:** † School of Chemistry, University of Southampton, Southampton SO17 1BJ, U.K.; ‡ Discovery Sciences IMED Biotech Unit, AstraZeneca, 310 Cambridge Science Park, Milton Road, Cambridge CB4 0WG, U.K.

## Abstract

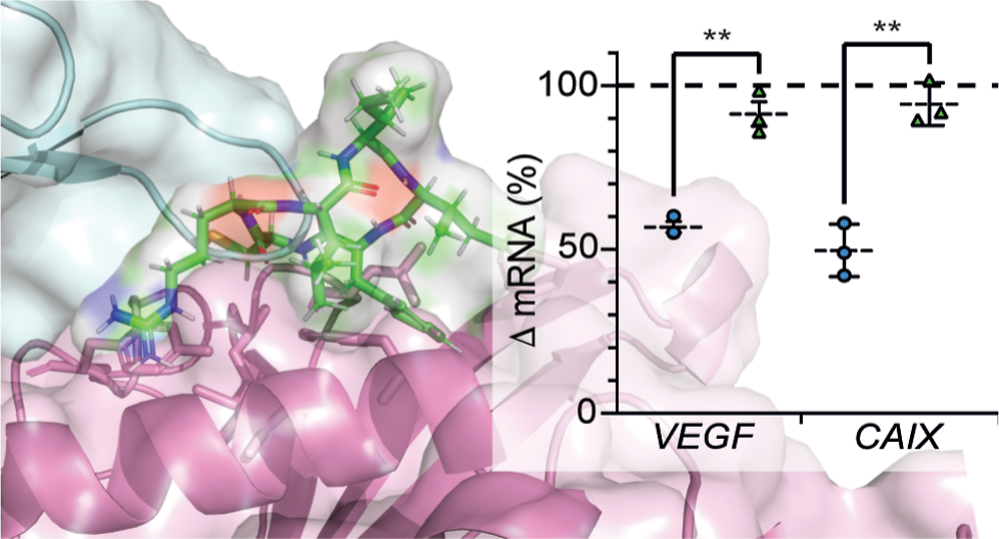

Hypoxia inducible factor (HIF) is a heterodimeric transcription factor composed of an oxygen-regulated α subunit and a constitutively expressed β subunit that serves as the master regulator of the cellular response to low oxygen concentrations. The HIF transcription factor senses and responds to hypoxia by significantly altering transcription and reprogramming cells to enable adaptation to a hypoxic microenvironment. Given the central role played by HIF in the survival and growth of tumors in hypoxia, inhibition of this transcription factor serves as a potential therapeutic approach for treating a variety of cancers. Here, we report the identification, optimization, and characterization of a series of cyclic peptides that disrupt the function of HIF-1 and HIF-2 transcription factors by inhibiting the interaction of both HIF-1α and HIF-2α with HIF-1β. These compounds are shown to bind to HIF-α and disrupt the protein–protein interaction between the α and β subunits of the transcription factor, resulting in disruption of hypoxia-response signaling by our lead molecule in several cancer cell lines.

## Introduction

Reduced intracellular oxygen concentration (hypoxia) plays a key role in multiple pathological conditions, such as cardiac arrest, stroke, and cancer.^1^ Hypoxia has particular relevance in cancer as solid tumors contain hypoxic regions that occur due to tumor growth exceeding the capacity of the surrounding vascular infrastructure.^2^ Hypoxia inducible factors (HIFs) are heterodimeric transcription factors that assemble in hypoxia and reprogram gene expression to allow survival and growth of cells in a low oxygen microenvironment.^3^ The expression of several hundred genes has been directly linked to HIF-1 activation, and genomic analysis of hypoxia-response element (HRE) sequences estimates that HIF-1 mediates the expression of up to 1% of the genome.^4,5^ While HIF activity impacts a diverse set of cellular pathways, the primary means by which hypoxic response is enacted is through the reprogramming of glucose metabolism, and the promotion of angiogenesis and proliferation.^6^ In tumors, HIF-mediated hypoxia-response promotes an aggressive phenotype that negatively correlates with patient outcome;^7,8^ genetic disruption of the HIF signaling pathway suppresses tumor growth and inhibition of HIF has long been proposed to be an attractive target for cancer therapy.^9−11^

HIF is a heterodimeric transcription factor, composed of an oxygen-sensitive α subunit and a constitutively expressed β subunit (also known as the aryl hydrocarbon nuclear receptor translocator, ARNT). The α-subunit of HIF is continually expressed but subject to post-translational modifications by oxygen-dependent proline hydroxylases (PHD).^12,13^ The hydroxylation of two prolines in HIF-1α (P402 and P564) enables recognition by the Von Hippel–Lindau protein and its associated E3 ligase complex, which triggers rapid ubiquitination and proteasomal degradation. In hypoxia, HIF-α degradation does not occur (due to the absence of the molecular oxygen required for prolyl hydroxylation), leading to an increase in the HIF-α concentration that causes its translocation to the nucleus, where it forms a dimeric complex with HIF-β to form the active HIF transcription factor. Thus, HIF activity is acutely oxygen-sensitive, with HIF-1α having a half-life of less than 5 min in normoxia.^14^ There are 3 isoforms of HIF-α (HIF-1α, HIF-2α, and HIF-3α), which all interact with HIF-1β. HIF-1α is expressed ubiquitously, whereas HIF-2α appears to be expressed in a more tissue-specific or environmentally conditional manner. The role of HIF-3α is less well understood, but this isoform is thought to be a regulator of HIF activity due to the absence of the C-terminal transactivation domain.^15^ Interestingly, HIF-1α and HIF-2α appear to have nonredundant roles that each produce distinct phenotypes due to their distinct target genes, and in tissues where both isoforms are expressed, they have synergistic roles in promoting the hypoxic response.^16^ Inhibition of the protein–protein interaction (PPI) between the α and β subunit of HIF is proposed as a key point of therapeutic intervention in the hypoxia-response pathway.^17^ The PAS-B domain of HIF-2α has a cavity that can accommodate small molecules, whereas this cavity is much smaller in HIF-1α; this difference has allowed the design of compounds that selectively inhibit the HIF-2α/HIF-1β PPI and inhibit HIF-2 activity in tumors,^18−20^ leading to a marketed drug (belzutifan) for the treatment of a subset of renal cell carcinoma (RCC) whose hypoxia response is solely driven by HIF-2.^21^ However, in a phase 2 clinical trial for belzutifan in patients with RCC associated with VHL disease, only 49% of patients responded, and all of these were partial responses.^22^ One hypothesis for this outcome is that the hypoxia response in this population’s tumors is being driven by both HIF-1 and HIF-2.^23−25^

We have previously reported a cyclic peptide (*cyclo*-CLLFVY) that specifically inhibits the interaction of HIF-1α with HIF-1β and disrupts HIF-1 activity in vitro and in cells.^26^ However, given the synergistic role played by HIF-1 and HIF-2 in cancer, a dual inhibitor of HIF-1 and HIF-2 would be of value, both as a research tool and as a starting point for the development of a therapeutic agent.

Here, we report cyclic peptide inhibitors of both the HIF-1α/HIF-1β and the HIF-2α/HIF-1β PPI, identified from a library of 3.2 million cyclic hexapeptides using a genetically encoded, intracellular high-throughput screening platform.^27^

## Results and Discussion

### Identification of the Inhibitors of HIF-1 and 2 Isoforms by SICLOPPS

**Figure 1:**
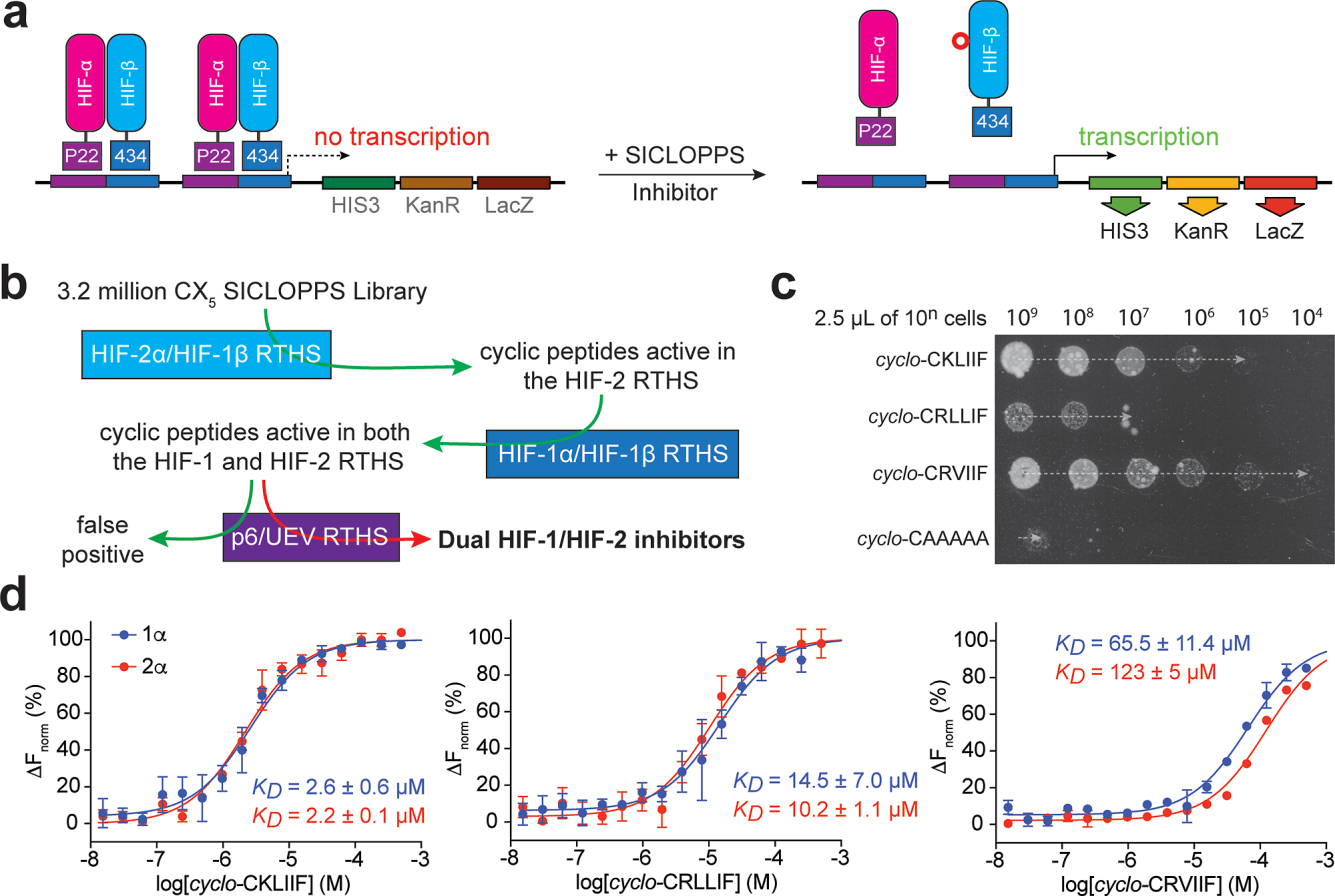
Identification of cyclic peptide inhibitors of HIF-1 and HIF-2. (a) Constructed RTHS links the HIF-1α/HIF-1β PPI to the life or death of the *E. coli* host via three reporter genes (HIS3, KanR, LacZ). (b) CXXXXX SICLOPPS library was screened in a HIF-2 RTHS, and plasmid-encoding hits in this screen were transformed into a HIF-1 RTHS and screened. Plasmid-encoding cyclic peptides that were active in both RTHS were transformed into a RTHS for an unrelated PPI (p6/UEV) and assayed. Plasmid-encoding peptides that were active in the HIF-1 and HIF-2 RTHS but not in the p6/UEV RTHS were potential dual HIF-1/HIF-2 inhibitors. (c) Activity of the top 3 most active plasmids assessed by drop-spotting in the HIF-1 RTHS, and the identity of the encoded cyclic peptides was revealed by DNA sequencing of the corresponding SICLOPPS plasmids. (d) Binding affinity of the top 3 most active cyclic peptides to the PAS-B domain of HIF-1α (blue) and HIF-2α (red) assessed by MST. All data are shown as mean (*n* = 2) ± SEM.

We used a previously reported genetically encoded platform that combines an intracellular split-intein circular ligation of peptides and proteins (SICLOPPS) library of 3.2 × 10^6^*cyclo*-CXXXXX cyclic peptides (CX_5_; where X = any canonical amino acid) with an *Escherichia coli* (*E. coli*) bacterial reverse two-hybrid system (RTHS) reporting on the HIF-2α/HIF-1β PPI.^26^ In this RTHS, each of the two targeted proteins (e.g., HIF-2α and HIF-1β) is expressed as an N-terminal fusion with a heterodimeric variant of the bacteriophage 434 repressor.^28^ Interaction of the targeted proteins leads to the formation of a functional repressor that suppresses expression of three reporter genes (HIS3, KanR, and LacZ), resulting in cell death on selective media.^29,30^ Library members that disrupt this PPI will also disrupt the assembly of the chimeric 434 repressor, which leads to the survival and growth of the RTHS on selective media (Figure 1a). To identify cyclic peptides that disrupt both the HIF-1α/HIF-1β and the HIF-2α/HIF-1β PPI (Figure 1b), a CX_5_ library was transformed into the HIF-2 RTHS with a transformation efficiency of 6.2 × 10^7^, giving ∼20-fold coverage of the library. Upon plating and incubation on selective media, 125 colonies were picked, and their SICLOPPS plasmids were isolated. These plasmids were transformed into a previously reported HIF-1 RTHS;^26,35^ surviving colonies were picked, and the SICLOPPS plasmids were isolated. False positives were identified by transforming the SICLOPPS plasmids identified as active in both HIF-1 and HIF-2 RTHS into a previously reported RTHS and monitoring the interactions between HIV p6 and the human TGS101 proteins (p6/UEV RTHS).^31,32^ Except for the target PPI (p6/UEV), this RTHS is identical to the HIF-1 and HIF-2 RTHS, therefore any hits that were active in the p6/UEV RTHS were discarded (Figure 1b). We observed growth enhancement in the p6/UEV RTHS for 11 of the 35 SICLOPPS plasmids, indicating that they were false positives or nonselective. The activity of the remaining 24 hits was ranked by drop-spotting in the HIF-1 RTHS; 3 SICLOPPS plasmids bestowed significant growth enhancement to the HIF-1 RTHS on selective media compared to a plasmid-encoding *cyclo*-CAAAAA as a control (Figure 1c). These 3 SICLOPPS plasmids were sequenced to reveal the identity of the encoded cyclic peptides as *cyclo*-CKLIIF, *cyclo*-CRVIIF, and *cyclo*-CRLLIF (Figure S1). These cyclic peptides were synthesized by solid-phase peptide synthesis, and their affinity for the PAS-B domain of HIF-1α and HIF-2α was measured by microscale thermophoresis (MST). The peptide *cyclo*-CKLIIF showed the greatest affinity for both the HIF-1α and HIF-2α PAS-B domains, with a *K*_D_ of 2.6 ± 0.6 μM and 2.2 ± 0.1 μM, respectively (Figure 1d). Likewise, *cyclo*-CRLLIF appeared to have similar affinity for both isoforms, with a *K*_D_ of 14.5 ± 7 and 10.2 ± 1.1 μM. In contrast, *cyclo*-CRVIIF had weaker affinity for both isoforms with a 2-fold selectivity for HIF-1α over HIF-2α, with a *K*_D_ of 65 ± 11 and 123 ± 5 μM, respectively (Figure 1d). The binding curve of *cyclo*-CKLIIF to HIF-1α had a Hill slope of 1.33, 1.38 to HIF-2α, and 1.22 and 1.45, respectively, for *cyclo*-CRLLIF, indicating 1:1 binding stoichiometry between these cyclic peptides and HIF-α proteins. The discrepancy in the order of activity between drop-spotting and measured *K*_D_ is likely a consequence of differences in expression and/or splicing rates, which would affect the intracellular concentration of each of the top 3 hits.

### Identification of an Active Pharmacophore

**Figure 2:**
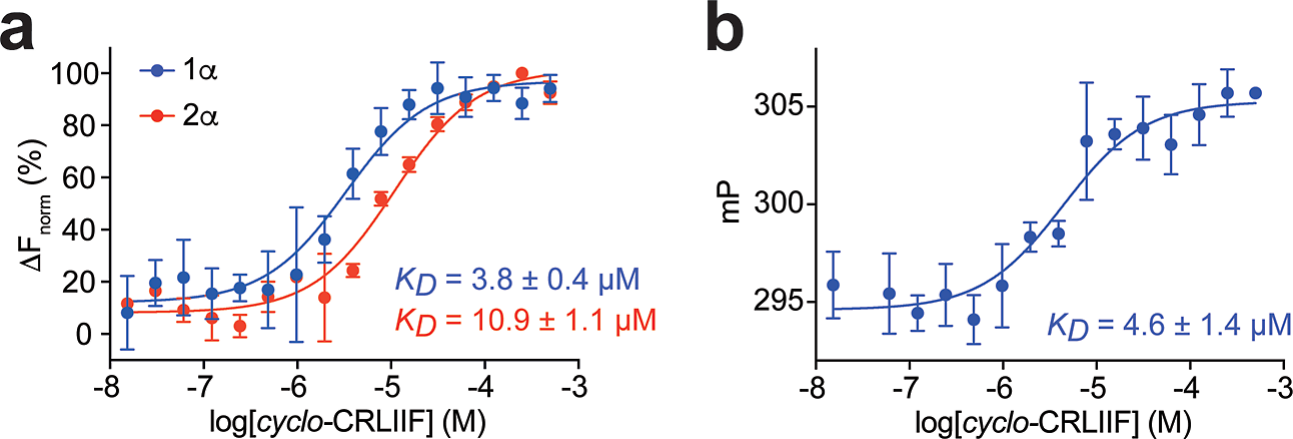
Assessing the binding of *cyclo*-CRLIIF to the PAS-B domain of HIF-α proteins. (a) Binding affinity of *cyclo*-CRLIIF to HIF-1α and HIF-2α by MST; data are shown as mean (*n* = 2) ± SEM. (b) Binding affinity of *cyclo*-CRLIIF to HIF-1α measured by FP; data are shown as mean (*n* = 3) ± SEM.

Despite the high degree of sequence homology between *cyclo*-CKLIIF and *cyclo*-CRLLIF, there is a ∼5-fold difference in their affinity for HIF-α proteins. We investigated whether this was due to the differing residues at position 2 (K or R) or position 4 (V or I) through the design and synthesis of 2 intermediary peptides, *cyclo*-CRLIIF and *cyclo*-CKLLIF, where the residues at the 2 or 4 positions had been swapped. These two cyclic peptides were synthesized and tested by MST for binding to HIF-1α PAS-B. Interestingly, *cyclo*-CRLIIF bound to HIF-1α with a *K*_D_ of 3.8 ± 0.4 μM (Figure 2a), while *cyclo*-CKLLIF bound to HIF-1α with a *K*_D_ of 12.0 ± 2.0 μM (Figure S2). The R2K-substitution was observed to have less of an effect than the L4I change, as the affinity for HIF-1α of *cyclo*-CRLIIF resembled that of *cyclo*-CKLIIF (3.8 vs 2.6 μM). Given their relative similarity in affinity for HIF-1α, *cyclo*- CRLIIF was chosen as the lead peptide for further derivatization, as the orthogonal protection required for the lysine residue of *cyclo*-CKLIIF during synthesis and cyclization reduced solubility, which led to significantly reduced yields of the cyclic peptide. A fluorescence polarization (FP) assay was developed and used to further verify and quantify the binding of *cyclo*-CRLIIF to the HIF-1α protein. A dose-dependent change in polarization was observed corresponding to a *K*_D_ of 4.6 ± 1.4 μM for the binding of *cyclo*-CRLIIF to the Cy5-labeled HIF-1α (Figure 2b), in line with the *K*_D_ observed for this cyclic peptide by MST.

**Figure 3:**
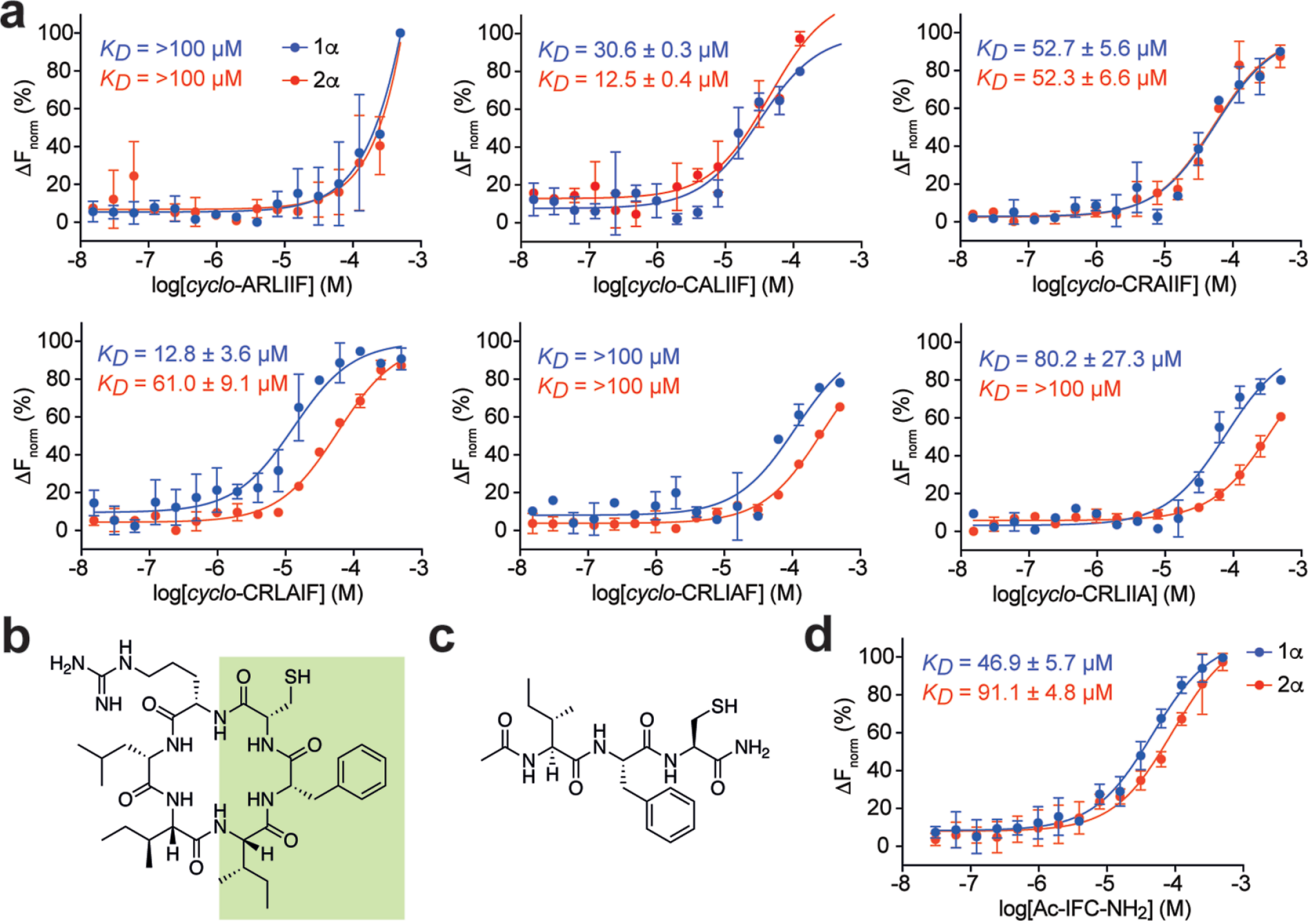
Identification of a tripeptide pharmacophore in *cyclo*-CRLIIF. (a) Binding affinity of the 6 alanine-scan derivatives of *cyclo*-CRLIIF to the PAS-B domain of HIF-1α (blue) and HIF-2α (red) assessed by MST. (b) Alanine scanning data indicates that a continuous IFC tripeptide (highlighted) is important for the binding of *cyclo*-CRLIIF to its target. (c) Capped analogue of the IFC tripeptide. (d) Binding affinity of the capped IFC tripeptide to the PAS-B domain of HIF-1α (blue) and HIF-2α (red) assessed by MST. All data are shown as mean (*n* = 2) ± SEM.

We used alanine scanning to identify the pharmacophore of *cyclo*-CRLIIF. The affinity of each alanine analogue for HIF-1α was measured by MST (Figure 3a); while all alanine substituents reduced the affinity of the peptide for the PAS-B domains to some extent (likely due to the effect of the alanine-substitution on the conformation and/or lipophilicity of the cyclic peptide), C1A, I5A, and F6A substitutions had the most deleterious effect on binding affinity (Figure 3a). The loss of activity of the C1A mutation (i.e., ARLIIF) was surprising, as the cysteine residue is present in position 1 of all library members, as it is required for intein splicing. CRLIAF and CRLIIA also exhibited large losses in binding affinity compared to the parent compound against both HIF-1α and HIF-2α. The alanine scanning data suggests that the contiguous IFC pharmacophore engages with the HIF-α proteins (Figure 3b), leading to disruption of the targeted PPI; this hypothesis is further supported by the presence of an IFC motif in all of the top 3 original hits (Figure 1d).

The necessity of cysteine 1 for target engagement raised the possibility that the interaction between the cyclic peptide and protein is mediated through disulfide bond formation with a cysteine residue on HIF-1α.^33^ While this was considered unlikely given the presence of the reducing agent tris(2-carboxyethyl)phosphine (TCEP) in excess in the MST buffers, the possibility was nonetheless directly probed using three mutant HIF-1α PAS-B proteins, each with 1 of its 3 cysteines replaced with alanine (C255A, C334A, and C337A; Figure S3a). We reasoned that if the formation of a disulfide bond with the target protein was required for activity, then *cyclo*-CRLIIF would not bind the mutant protein, where the target cysteine has been replaced with alanine. We observed that *cyclo*-CRLLIF bound to the 3 C-to-A mutant HIF-1α proteins with similar affinity to the wild-type (Figure S3b–d), indicating that the interaction between *cyclo*-CRLIIF and HIF-1α is not mediated by a disulfide bond.

Previous work from our group has shown that the linear peptide pharmacophore of a cyclic peptide is capable of target engagement and inhibition,^34,35^ we therefore assessed whether the IFC pharmacophore identified above binds HIF-1α and HIF-2α as a linear tripeptide. We synthesized a variant of the IFC tripeptide acetyl capped at the N-terminus and a C-terminal amide (Figure 3c), reasoning that the resulting termini better resemble the characteristics of these groups when contained within a cyclic peptide backbone (e.g., ionization at physiological pH). The resulting tripeptide bound to HIF-1α with a *K*_D_ of 46.9 ± 5.7 μM and HIF-2α with a *K*_D_ of 91.1 ± 4.8 μM (Figure 3d), further confirming the role of this tripeptide in the interaction of *cyclo*-CRLIIF with HIF-α proteins. The ∼10-fold loss of affinity observed (cf. cyclo-CRLIIF) is consistent with our previous observation^35^ and is likely due to the loss of conformational rigidity associated with being constrained within a macrocyclic scaffold.

### Modeling the *cyclo*-CRLIIF Binding Site on HIF-α

**Figure 4:**
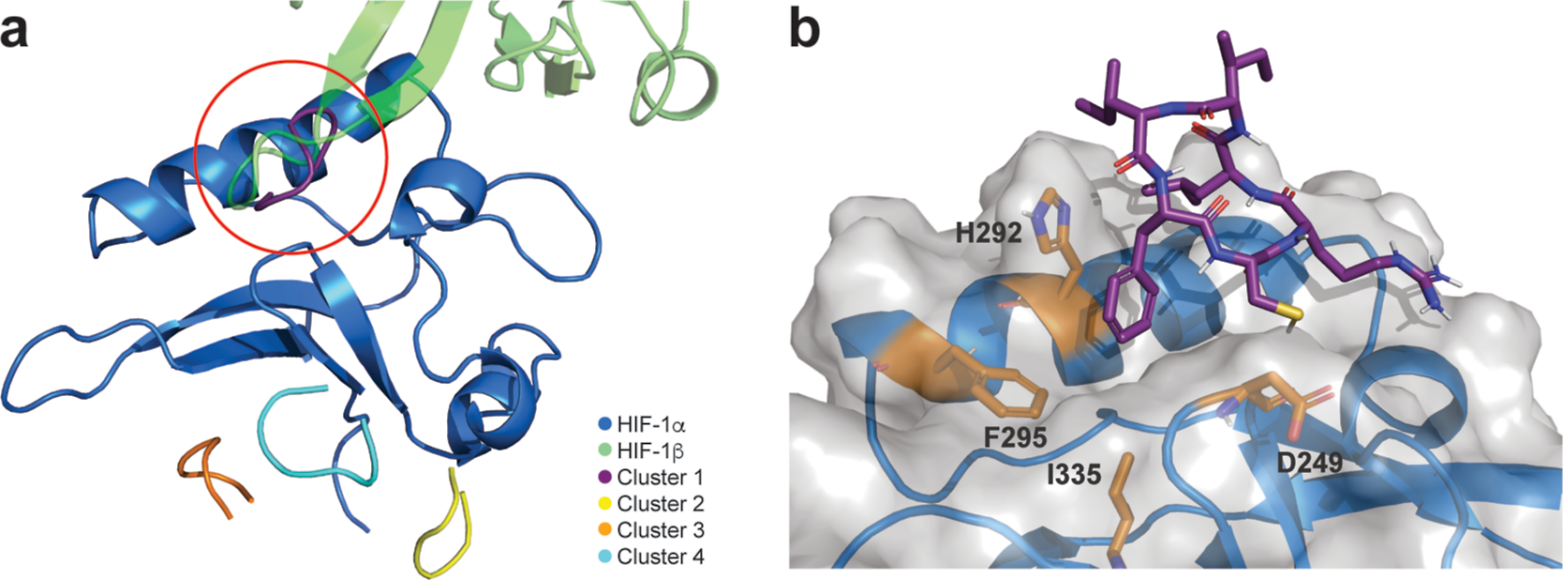
Simulation and docking derived poses for *cyclo*-CRLIIF binding to the HIF-1α PAS-B domain. (a) Representative structures for the top four clusters from REST2 simulations of *cyclo*-CRLIIF docking to the HIF-1α PAS-B domain; red circle highlighting the area of binding overlap between cluster 1 and HIF-1β. (b) Primary binding mode of *cyclo*-CRLIIF as identified through REST2 simulations with key interacting protein residues depicted atomistically, labeled, and colored orange.

The interaction between *cyclo*-CRLIIF and HIF-1α was modeled using replica exchange with solute scaling (REST2) enhanced sampling molecular dynamics simulations.^36^ The most populated binding site of *cyclo*-CRLIIF (Cluster 1, Figure 4a), constituting 14.2% of the simulation frames, directly overlapped a loop from HIF-1β (Figure 4a). To validate this observation further, we conducted molecular docking of the REST2-derived (Figure S4a) and a Rosetta-derived (Figure S4b) *cyclo*-CRLIIF structure to HIF-1α using HADDOCK.^37^ The docking of both conformations (Figure S4c,d) identified the same binding site as the REST2 simulations (Figure 4a) is within the top three clusters. We evaluated the exact binding poses and key interactions of *cyclo*-CRLIIF proposed by our REST2 simulations and molecular docking based on the alanine scanning data (Figure 3a), which indicate that the C1, I5, and F6 residues are critical for binding. Visualization of the HADDOCK docked binding poses from our REST2-derived starting structure does not indicate any critical interactions between residues Ile5 or Phe6 to HIF-1α (Figure S4a). HADDOCK docked poses from our Rosetta-derived starting structure did not show any interaction between Cys1 and HIF-1α (Figure S4b), suggesting that the correct binding pose has not been identified through static docking approaches. Subsequently, we analyzed the binding poses of our REST2 simulation data and measured the hydrogen bonding interaction frequency between the peptide Cys1 across the simulation to all other heavy atoms in the system. The most frequent interaction was found to be between Cys1 and the backbone oxygen of D249 observed in 10.3% of the simulation frames (representative conformation shown in Figure 4b). We observed that conformations with this interaction had a buried F6 side chain driven by the hydrophobic pocket created by I335 and F295. However, I5 remained largely solvent exposed, with weak interactions toward H292 seen in 3.8% of simulation frames. The alanine scanning data indicates that I5 has a greater effect on binding than F6, which is either due to I5 playing a key role in the conformation of *cyclo*-CRLIIF (rather than being directly binding to HIF-1α) or because we have not captured the true binding pose in our simulations. Nonetheless, it should be noted that the interaction interface is consistent with the experimental results, indicating that the former has been correctly identified. This observation suggests that the residue 234–237 loop-turn region may be critical for mediating initial recruitment of the *cyclo*-CRLIIF peptide to the target binding site. Further analysis using LoopFinder^38^ identified residues 216–226 on HIF-1β as a “hotloop,” indicating that it is a potential hotspot that mediates the HIF-1α/HIF-1β PPI. In our proposed binding model, *cyclo*-CRLIIF disrupts the binding of this HIF-1β “hotloop” to HIF-1α. Molecular docking of *cyclo*-CRLIIF with the PAS-B domain of HIF-2α was also conducted. The top scoring cluster for both the REST-2-derived (Figure S4e) and Rosetta-derived (Figure S 4f) Same site on HIF-2α as that identified for HIF-1α. Together, this data provides a possible explanation for the disruption of both the HIF-1 and HIF-2 PPI by our cyclic peptide.

### Optimizing the Activity of *cyclo*-CRLIIF

**Figure 5:**
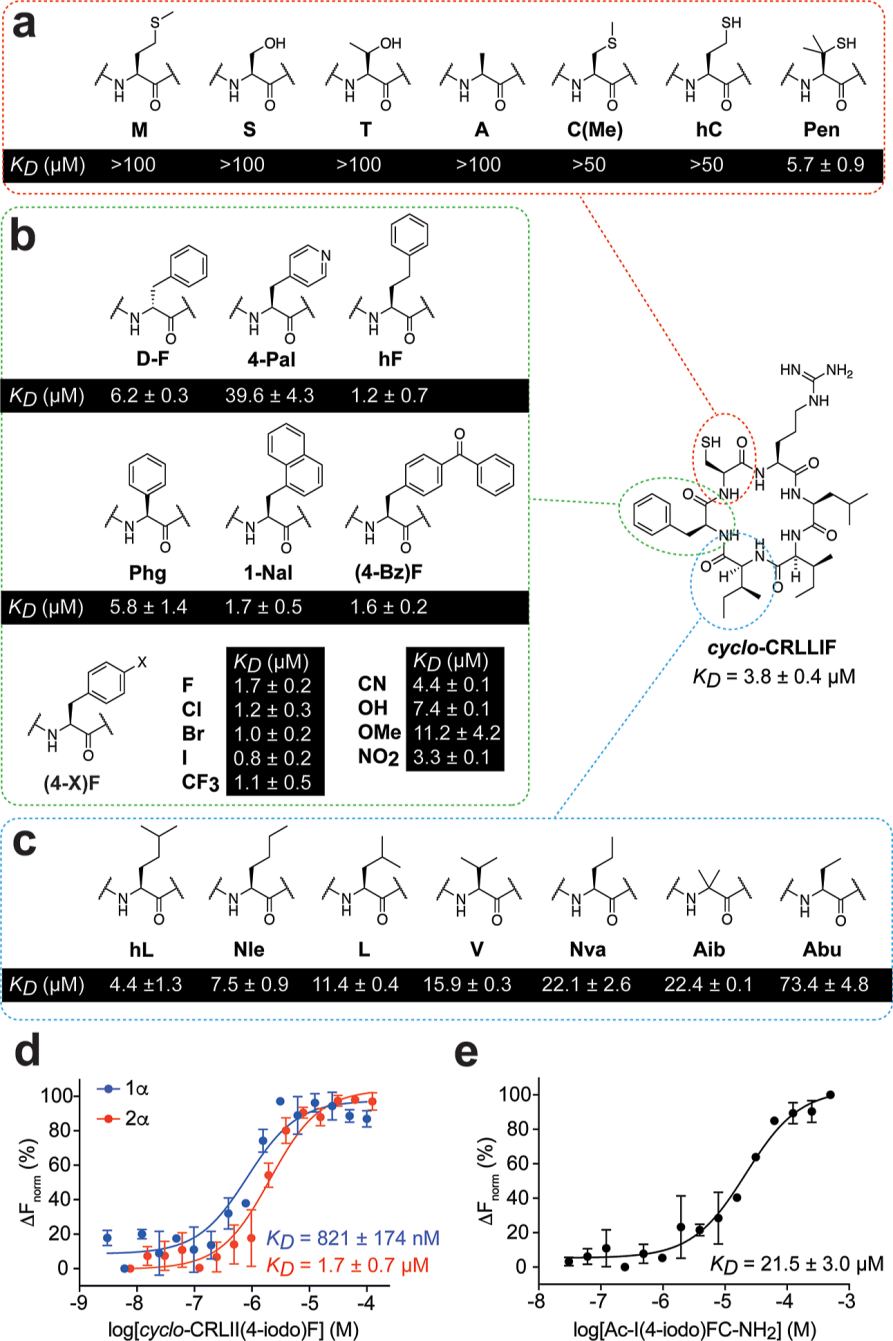
Optimizing the activity of *cyclo*-CRLIIF. (a) Substituents used for cysteine 1 and their resulting *K*_D_; binding curves in Figure S5. (b) Substituents used for phenylalanine 6 and their resulting *K*_D_; binding curves are in Figure S6. (c) Substituents used for isoleucine 5 and their resulting *K*_D_; binding curves are shown in Figure S7. (d) Binding affinity of cyclo-CRLII(4-iodo)F to the PAS-B domain of HIF-1α (blue) and HIF-2α (red) assessed by MST. (e) Binding affinity of Ac–I(4-iodo)FC-NH_2_ to the PAS-B domain of HIF-1α by MST. All data are shown as mean (*n* = 2) ± SEM.

We next aimed to optimize the binding affinity of *cyclo*-CRLIIF for HIF-1α via the incorporation of non-natural amino acids into the 3 pharmacophore residues C1, I5, and F6. Seven C1-substituted analogues were synthesized (Figure 5a). The substituent amino acids were primarily chosen to probe the requirement for a primary sulfur as well as to assess the optimal length of the side chain. Changes included replacing sulfur with oxygen (**S** and **T**), extending the length of the side chain by one carbon (**hC**), and S-methylation [**M** and **C(Me)**]; in all cases, however, replacement of cysteine was detrimental to affinity. Only the penicillamine derivative (**Pen**) bound HIF-1α with an affinity similar to that of the parent molecule, indicating that the steric bulk of the additional methyl groups on the β carbon does not significantly affect binding.

We synthesized and tested 15 cyclic peptide derivatives containing unnatural aromatic amino acid substitutions at the F6 position (Figure 5b). The positioning of the phenyl group was initially explored; the extension of the aliphatic side chain by one methylene through the homophenylalanine (**hF**) improved binding 3-fold to 1.2 ± 0.7 μM, whereas attachment of the phenyl group directly to the α-carbon (**Phg**) reduced affinity to a *K*_D_ of 5.8 ± 1.3 μM. Reversing the stereochemistry at the α-carbon (**D–F**) also resulted in a minor loss of binding activity (*K*_D_ of 6.2 ± 0.3 μM). Replacing the benzene ring with a pyridine (**4-Pal**) caused ∼11-fold loss in *K*_D_ to 39.6 ± 4.3 μM. The larger aromatic naphthalene (**1-Nal**) and benzophenone [**(4-Bz)F**] appeared to be accommodated in the binding pocket with a *K*_D_ of 1.7 ± 0.5 and 1.6 ± 0.2 μM, respectively.

We also synthesized a series of para-substituted phenylalanine derivatives to probe the requirements of this position. We observed a 13-fold range in *K*_D_ between the most potent derivative, 4-iodophenylalanine [**(4-I)F**], at 0.8 ± 0.2 μM and the weakest, 4-methoxyphenylalanine [**(4-OMe)F**], at 11.2 ± 4.2 μM.

We synthesized and tested 8 derivatives of *cyclo*-CRLIIF with aliphatic non-natural amino acids at the I5 position (Figure 5c). The replacement groups were chosen to probe the optimal length and methylation of the hydrocarbon chain. While most substituents resulted in a weaker binding cyclic peptide, *K*_D_ values correlated with the length of the side chain. Branched-chain amino acids (**hL**, **L**, **V**, and **Aib**) exhibited improved activity over their linear chain counterparts (**Nle**, **Nva**, and **Abu**), indicating that structure and not just hydrophobicity is important for optimal binding at this position.

The (4-iodo)F-containing derivative, *cyclo*-CRLII(4-iodo)F, was the most potent cyclic peptide in our series, binding to HIF-1α with a *K*_D_ of 821 ± 147 nM, a 4.6-fold increase in binding affinity compared to the parent cyclic peptide. The affinity of this compound for HIF-2α PAS-B was measured as 1.7 ± 0.7 μM (Figure 5d), representing a 6.5-fold improvement in *K*_D_.

We sought to establish whether 4-iodophenyl substitution similarly improves the affinity of the capped derivative of the IFC pharmacophore (Figure 3c). We synthesized the 4-iodo derivative of this molecule [Ac–I(4-iodo)FC-NH_2_] and found that it binds to HIF-1α with a *K*_D_ of 21.5 ± 3.0 μM (Figure 5e). The ∼3 -fold improvement in affinity over the parent tripeptide molecule is in line with that observed for the cyclic peptide analogues and is further evidence for an IFC pharmacophore in the parent molecule.

We also assessed whether *cyclo*-CRLII(4-iodo)F was able to bind to G323E HIF-2α, a previously reported mutation in HIF-2α that confers resistance to PT2399 (an earlier derivative of the marketed drug belzutifan) by blocking the PT2399-binding pocket in HIF-2α.^39^ We observed *K*_D_ values of 7.2 ± 0.6 μM (Figure S8), an 8.7-fold reduction in binding affinity compared to the wild type protein (Figure 5d). While this data shows that *cyclo*-CRLII(4-iodo)F binds to G323E HIF-2α, the observed reduction in binding affinity (cf. wild-type protein, Figure 5d) suggests that the binding site of our cyclic peptide may also be affected by this mutation, either directly or indirectly (e.g., via structural changes to the binding site).

### Assessing the Activity of *cyclo*-CRLII(4-iodo)F in Cells

**Figure 6:**
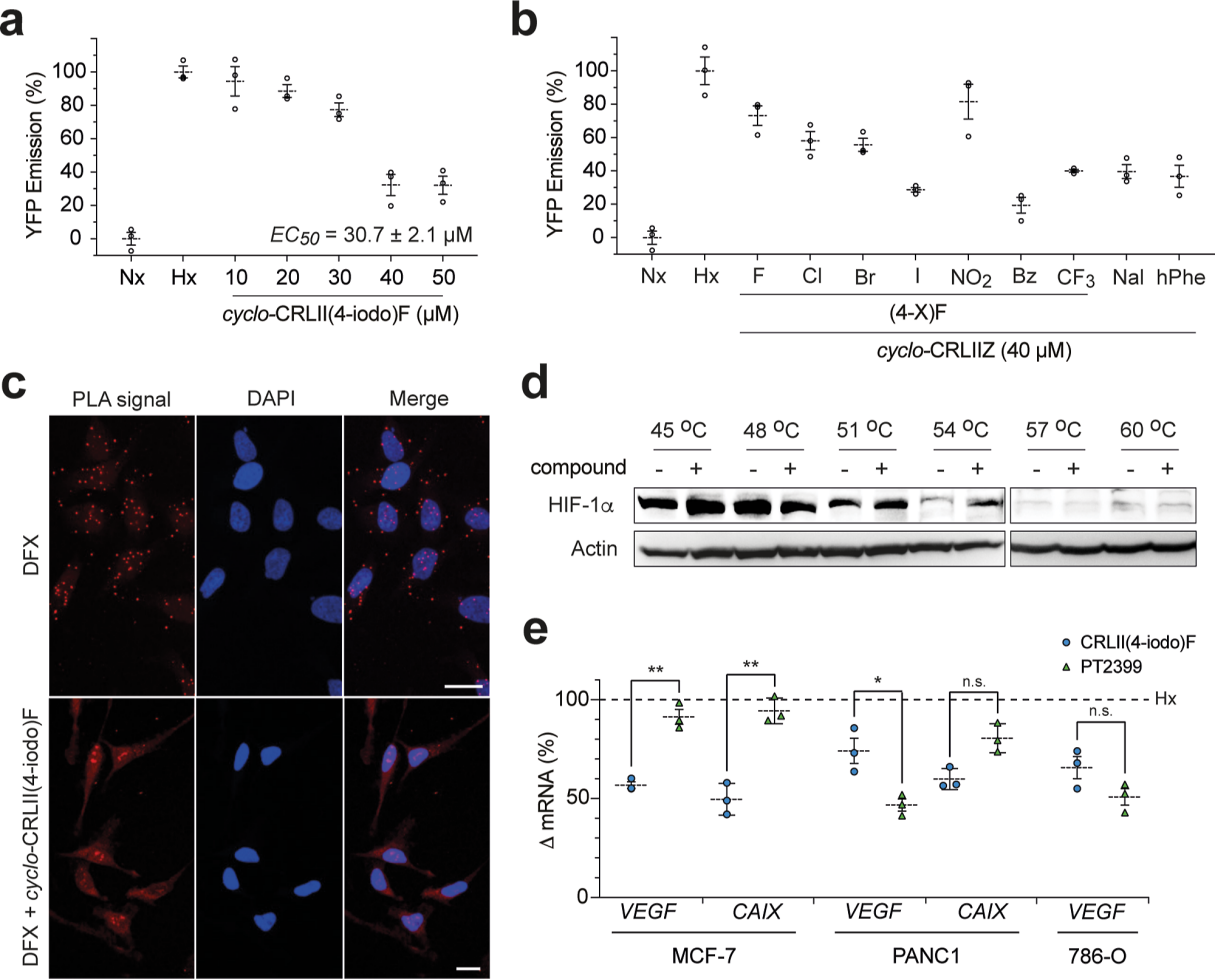
Assessing the activity of *cyclo*-CRLII(4-iodo)F in cells. (a) *cyclo*-CRLII(4-iodo)F reduces the HIF-driven expression of YFP in a T-REx-293 reporter cell line, Nx = normoxia, Hx = hypoxia. (b) Effect of the 9 most potent (by MST) *cyclo*-CRLIIF analogues on YFP expression in a T-Rex-293 reporter cell line, Nx = normoxia, Hx = hypoxia. (c) PLA assay is used to illustrate that *cyclo*-CRLII(4-iodo)F inhibits the HIF-1α/HIF-1β PPI in HeLa cells treated with the hypoxia mimetic DFX. The upper panels (DFX-treated cells) show the red puncta associated with a positive PLA signal, indicating the presence of the HIF-1α/HIF-1β PPI. The red puncta are absent in the images in lower panels [DFX + *cyclo*-CRLII(4-iodo)F], indicating disruption of the targeted PPI. (d) Cellular thermal shift assay was used to demonstrate the binding of *cyclo*-CRLII(4-iodo)F to HIF-1α in cells, as shown by the 2.9 ± 0.4-fold higher intensity band in compound-treated samples at 54 °C. (e) Effect of *cyclo*-CRLII(4-iodo)F (50 μM) or PT2399 (2 μM) on the expression of *CAIX* and *VEGF* genes in MCF-7, Panc-1, and 786-O cells by qPCR; data are normalized to the levels of each gene in hypoxia for each cell line (Hx = hypoxia, dotted line). All data are shown as mean (*n* = 3) ± SEM; **p < 0.01, *p < 0.05.

Following in vitro characterization and optimization of binding, we next assessed the effect of our cyclic peptides in mammalian cells. A cell line with a stably integrated reporter was constructed, in which the expression of yellow fluorescent protein (YFP) was placed under the control of a HRE. Thus, hypoxia would be expected to increase YFP expression (and subsequent fluorescence) in these cells, whereas treatment with a cell-permeable HIF inhibitor would be expected to reduce this fluorescence signal. A fusion cassette was designed containing the YFP gene preceded by three copies of the HRE sequence. This cassette was stably integrated into T-REx-293 cells as previously described.^12^ We assessed the activity of *cyclo*-CRLII(4-iodo)F in this cell line, and observed a dose-dependent reduction in the hypoxia-mediated YFP fluorescence with an EC_50_ of 30.7 ± 2.1 μM (Figure 6a). The effect of this compound on the viability of T-Rex-293 was assessed with no effect observed at doses up to 75 μM (Figure S9a). We next tested the effect of the 9 most potent *cyclo*-CRLIIF derivatives in vitro (Figure 5) in this cell line. It should be noted that the relative activity of each compound in this assay will be a combination of its cell permeability and HIF-inhibiting activity. We observed a range of cell activity for these molecules, suggesting that despite their relative similarity in in vitro activity, there is variance in their cell permeability. The **(4-NO**_**2**_**)Phe** analogue was the least active, and the **(4-I)Phe** and **(4-Bz)Phe** derivatives were the most active (Figure 6b). We therefore continued to further characterize the cell activity of the 4-iodo-phenylalanine derivative.

We used a previously reported^26^ proximity-ligation assay (PLA) to assess whether *cyclo*-CRLII(4-iodo)F disrupts the HIF-1α/HIF-1β PPI in cells. HeLa cells were treated with the hypoxia mimic desferrioxamine (DFX) and treated with either DMSO (control) or 50 μM of *cyclo*-CRLII(4-iodo)F; in the control-treated samples, we observed the red puncta associated with the PLA signal from the targeted PPI (Figure 6c, upper panels). Whereas in cells treated with DFX and 50 μM *cyclo*-CRLII(4-iodo)F, the red puncta were not observed, illustrating that this molecule disrupts the HIF-1α/HIf-1β PPI in cells (Figure 6c, lower panels).

Next, we used a cellular-thermal shift assay^40^ to assess whether our lead molecule binds to HIF-1α in the intracellular environment. We observed a 2.9 ± 0.4-fold increased level of HIF-1α protein in Panc-1 (pancreatic cancer) cells treated with 40 μM *cyclo*-CRLII(4-iodo)F compared to DMSO control in samples heated to 54 °C, indicating intracellular binding of this compound to its protein target (Figure 6d).

The effect of our lead inhibitor on hypoxia-response signaling was measured by qPCR in cancer cell lines. Cells were treated with either 2 μM of an analogue of the HIF-2-specific inhibitor belzutifan, PT2399 (control), or 50 μM of *cyclo*-CRLII(4-iodo)F, incubated in hypoxia for 24 h, and the levels of the hypoxia-response genes *CAIX*(41,42) and *VEGF*(43) were measured by qPCR. We observed a 51% drop in *CAIX* transcription and a 43% drop in *VEGF* transcription in hypoxic MCF-7 (breast cancer) cells treated with 50 μM of *cyclo*-CRLII(4-iodo)F versus hypoxic MCF-7 cells treated with DMSO only (Figure 6e). The HIF-2-specific inhibitor PT2399 did not inhibit the hypoxia-driven expression of either VEGF or CAIX in MCF-7 cells (Figure 6e), indicating that in MCF-7 cells, hypoxia response is mainly mediated by HIF-1. Treatment with *cyclo*-CRLII(4-iodo)F also resulted in a 41% drop in *CAIX* expression in hypoxic Panc-1 (pancreatic cancer) cells and a 26% drop in *VEGF* transcription compared to DMSO-treated hypoxic cells (Figure 6e). Treatment with PT2399 resulted in a 23% drop in *CAIX* transcription and a 53% drop in *VEGF* transcription (Figure 6e), indicating that HIF-2 plays a partial role in the hypoxia response in Panc-1 cells. We also observed a 49% drop in *CAIX* expression in hypoxic HeLa (cervical cancer) cells treated with *cyclo*-CRLII(4-iodo)F (Figure S9b). To further assess the HIF-2 inhibition activity of this compound, we used 786-O cells, a ccRCC cell line that does not express functional HIF-1α, with high levels of constitutively expressed HIF-2α.^44^ We observed a 35% drop in HIF-2-driven *VEGF* transcription in 786-O cells treated with 50 μM of *cyclo*-CRLII(4-iodo)F compared to a 50% decrease in 786-O cells treated with PT2399. Together, our data demonstrate that *cyclo*-CRLII(4-iodo)F inhibits hypoxia-response signaling in a variety of cancer cell lines by binding to the PAS-B domain of the HIF-α protein and disrupting the PPI between the α and β subunits of HIF.

## Conclusions

Given the central role played by HIF proteins in the survival and growth of solid tumors, there is significant potential for a dual HIF-1/HIF-2 inhibitor for the treatment of a variety of cancers. While the relative contribution of each isoform to the survival and growth of tumors is dependent on multiple factors, including tumor type and stage of tumor, there is significant clinical evidence linking elevated levels of both HIF-1α and HIF-2α to poor patient outcomes in a wide variety of cancers. Furthermore, nuclear expression of both HIF-1α and HIF-2α has been observed in the majority of patient samples examined in a wide range of solid tumors, including breast, colon, ovarian, and pancreatic, indicating that both isoforms (HIF-1 and HIF-2) are driving hypoxia response in these tumors.^45^ Thus, the therapeutic strategy of inhibiting both HIF-1 and HIF-2 can be reasonably envisaged to be superior to the inhibition of just a single HIF isoform for most cancers. But the 48% amino acid homology between these proteins has made the identification of a direct dual HIF-1/HIF-2 inhibitor challenging by traditional drug discovery approaches. We used a genetically encoded library of 3.2 × 10^6^ cyclic peptides in combination with a cell-based assay to identify cyclic peptides capable of disrupting both the HIF-1 and HIF-2 PPI. The 3 lead molecules identified in this screen all contained the same tripeptide pharmacophore that was shown to bind the HIF-1α protein when synthesized as a capped tripeptide. In previous work, we have shown that a similarly capped dipeptide pharmacophore of a cyclic peptide hit (named compound **14**) retains target activity in vitro and in cells and is functional in a variety of in vivo models.^34^ Further chemical development of the IFC tripeptide pharmacophore is required to improve its affinity, and this is one possible route for taking these molecules forward. Our computational studies indicated that *cyclo*-CRLIIF binds to the same site on both HIF-1α and HIF-2α and that this site overlaps with a key binding loop from HIF-1β, providing a potential explanation for how this peptide disrupts both the HIF-1α/HIF-1β and HIF-2α/HIF-1β PPI. Our experimental and modeling data also illustrate that the binding site of our cyclic peptides are different to that of the selective HIF-2 inhibitor belzutifan.

The recent progress toward the clinic of a handful of cyclic peptides, identified *de novo* from genetically encoded libraries,^46,47^ has further demonstrated the value and therapeutic potential of this class of molecules and their use in drug discovery against the most challenging targets.^48,49^ Similarly, the cyclic peptides reported here may be further developed via the incorporation of non-natural amino acids and backbones in a similar approach as that taken for other larger cyclic peptides.^49,50^ As a starting point, we synthesized a small library of analogues with non-natural amino acids, which resulted in several cyclic peptides with improved activity. Cell-based assays showed that several members of this library were cell permeable and inhibitors of hypoxia-response signaling in a variety of cell lines by binding to HIF-α and disrupting the HIF-1α/HIF-1β and HIF-2α/HIF-1β PPI.

Concurrent optimization of the lead structure against HIF-1 and HIF-2 and its development as a dual inhibitor will pose unique challenges, with the need to drive SAR simultaneously for both targets. Nonetheless, given the central role played by HIF-1 and HIF-2 in the survival and growth of the majority of solid tumors and the direct correlation between HIF-α levels and patient mortality in many cancers,^51,52^ a therapeutic agent that directly inhibits both HIF-1 and HIF2 is expected to be of significant benefit to cancer patients.

## Data Availability

The data supporting the findings of this study are available within the paper and its Supporting Information. The raw data underpinning this study are openly available from the University of Southampton data repository: https://doi.org/10.5258/SOTON/D2988.

## Supporting Information Available

Structures of the top 3 HIF-1α/HIF-1β PPI inhibitors identified in this study, MST dose-response curves, atomistic structures of HADDOCK docked binding poses, materials and methods, and compound data containing HPLC and MS data for all compounds (PDF)
