# Supplementary material for: Identification and Development of Cyclic Peptide Inhibitors of Hypoxia Inducible Factors 1 and 2 That Disrupt Hypoxia-Response Signaling in Cancer Cells
Source: J Am Chem Soc. 2024 Mar 19;146(13):8877–86. doi: 10.1021/jacs.3c10508 (PMC10996005; doi:10.1021/jacs.3c10508)
Supplement: Supplementary file 1 — ja3c10508_si_001.pdf [file ja3c10508_si_001.pdf]

Supplementary Data

Identification and development of cyclic peptide inhibitors of Hypoxia Inducible Factors 1 and 2 that disrupt hypoxia-response signaling in cancer cells

Andrew T. Ball,[1,†] Soran Mohammed,[1,†] Cyrielle Doigneaux,[1] Reece M. Gardner,[1] James W. Easton,[1] Steven Turner,[1] Jonathan W. Essex,[1] Garry Pairaudeau,[2] and Ali Tavassoli*[1]

1. School of Chemistry, University of Southampton, Southampton, SO17 1BJ, U.K.
2. Discovery Sciences IMED Biotech Unit, AstraZeneca, 310 Cambridge Science Park, Milton Road, Cambridge CB4 0WG, U.K.
† Authors contributed equally
* e-mail: ali1@soton.ac.uk

Supplementary Figures

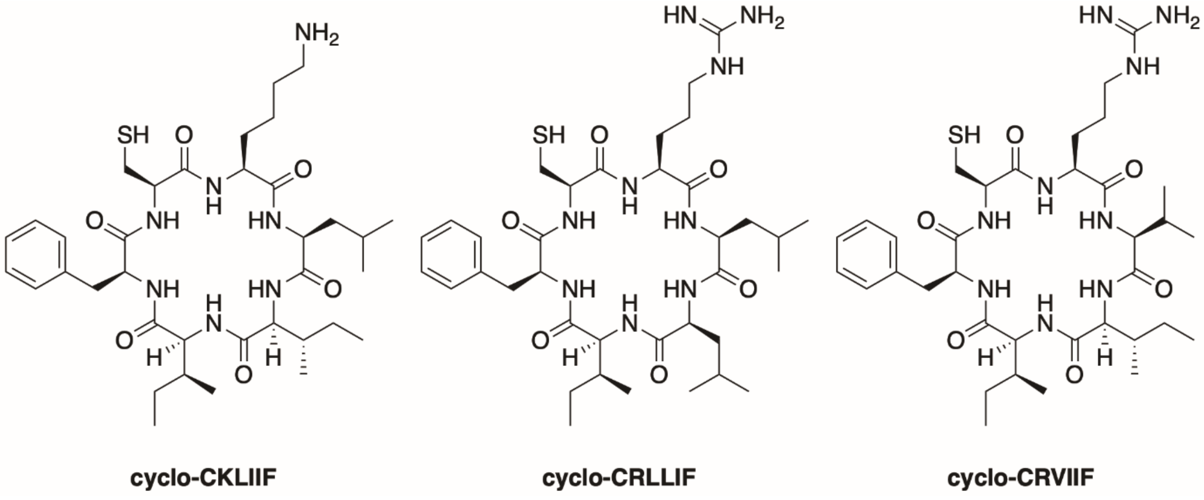

Figure S1. Structures of the top 3 HIF-1α/HIF-1β PPI inhibitors identified in this study.

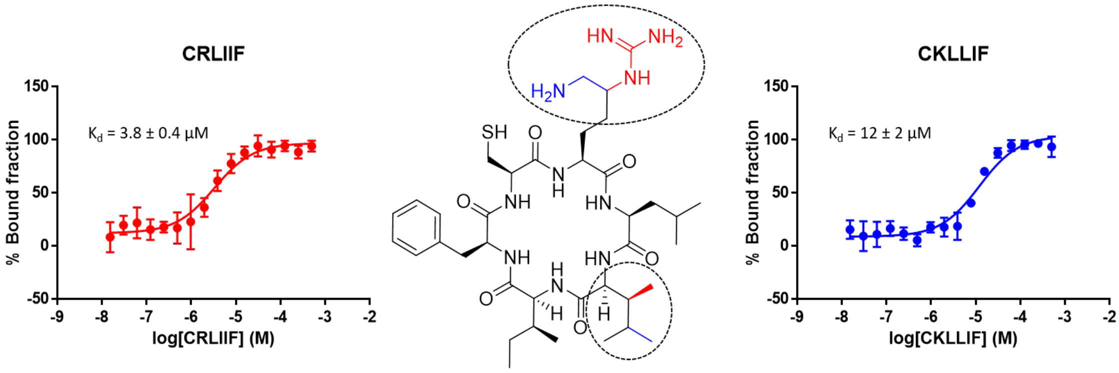

Figure S2. MST dose-response curves against 1α PAS-B, and comparison of the structures of CRLIIF and CKLLIF. Residues are colour-coded according to the differences in side chain structure between the peptides. All data is shown as mean (n=3) ± SEM.

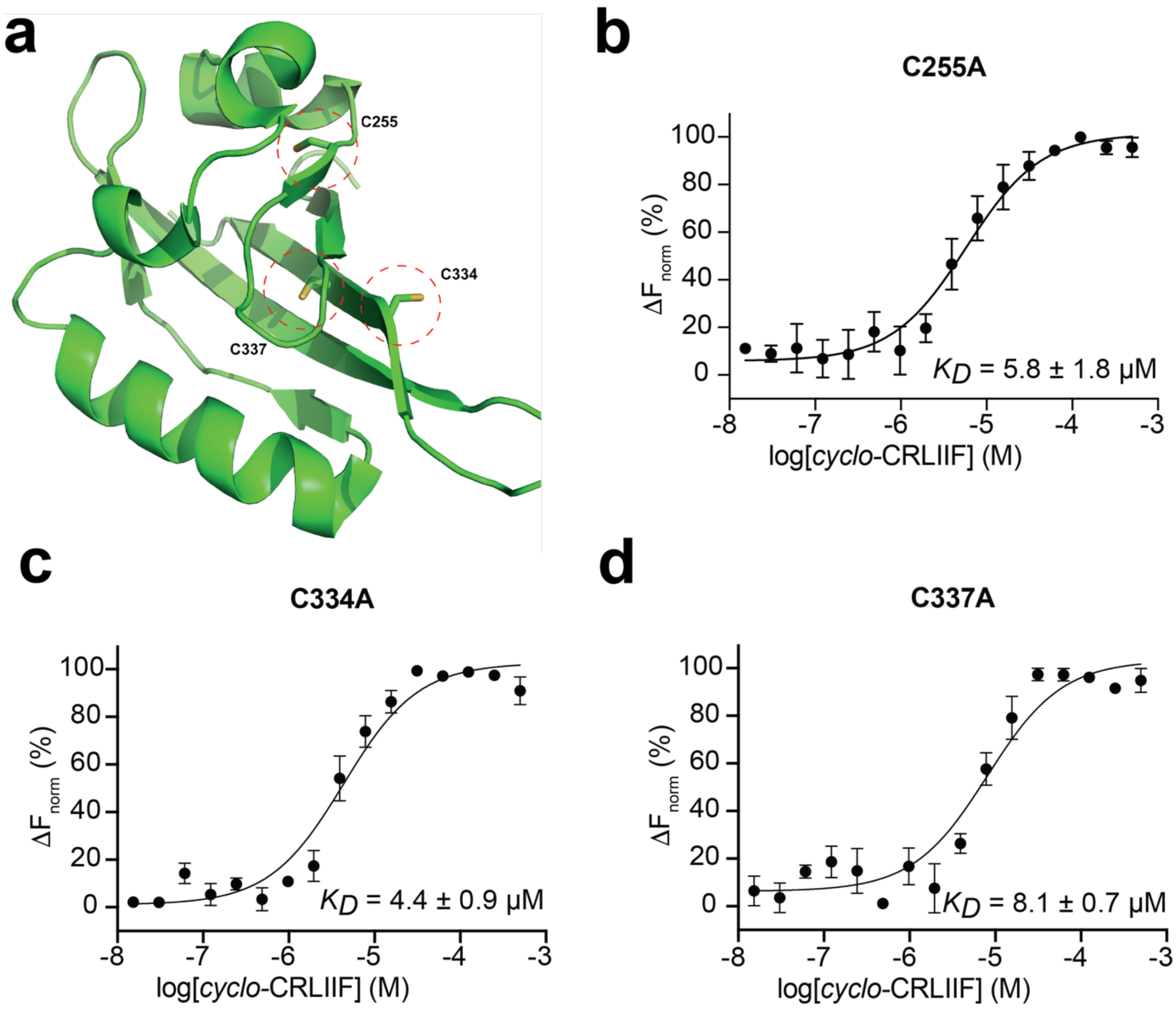

Figure S3. Assessing the binding of CRLIIF to cysteine mutants of HIF-1α PAS-B. (a) Structure of the HIF-1α PAS-B domain with the cysteine residues highlighted (adapted PDB 4H6J). **(b)** Affinity of C255A 1α-PAS-B for CRLIIF by MST. **(c)** Affinity of C334A 1α-PAS-B for CRLIIF by MST. **(d)** Affinity of C337A 1α-PAS-B for CRLIIF by MST. All data is shown as mean (n=3) ± SEM.

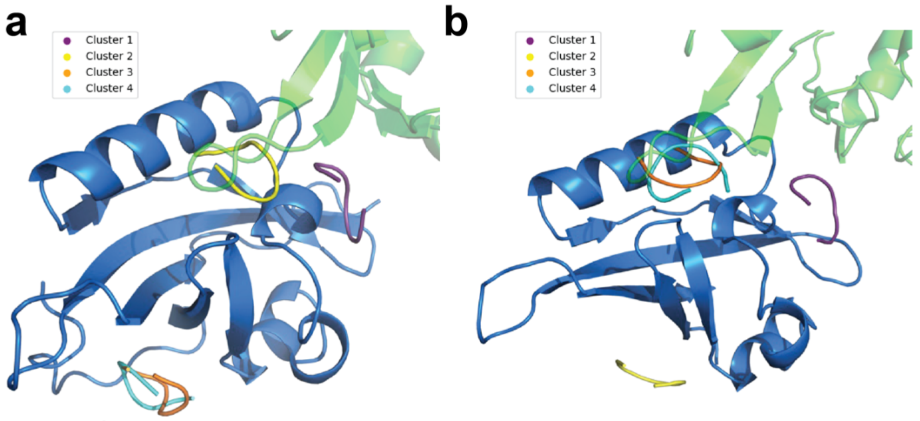

Cluster	Cluster average HADDOCK Score	Representative structure HADDOCK score
1	82.1	73.311
2	90.2	83.333
3	90.8	73.378
4	94.7	79.967

Cluster	Cluster average HADDOCK Score	Representative structure HADDOCK score
1	68.8	62.269
2	71	45.788
3	71.4	62.837
4	81.7	79.136

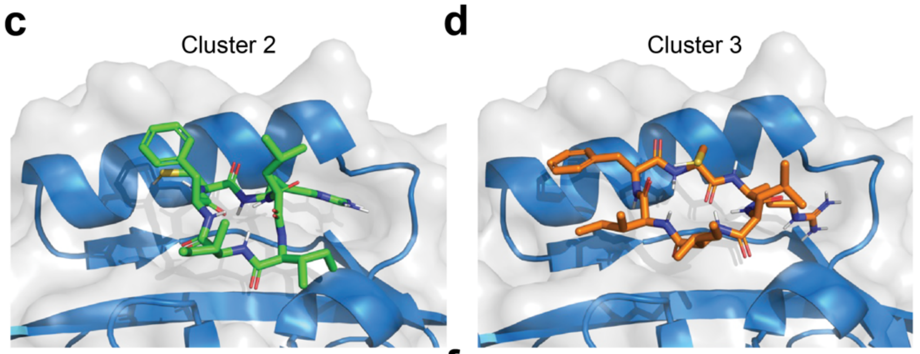

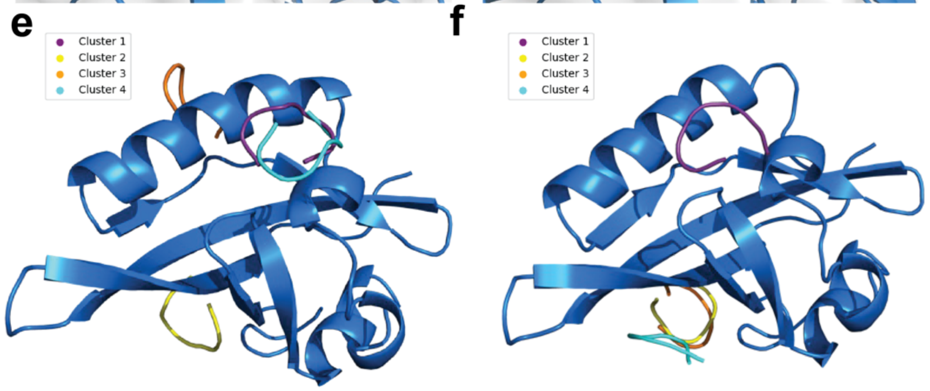

Cluster	Cluster average HADDOCK Score	Representative structure HADDOCK score
1	84.9	76.866
2	85.4	68.948
3	86.3	80.036
4	88.2	82.069

Cluster	Cluster average HADDOCK Score	Representative structure HADDOCK score
1	80.5	61.088
2	82.9	79.612
3	83.5	78.745
4	85.7	70.212

Figure S4. Atomistic structures of HADDOCK docked binding poses. (a) Representative structures for the top four HADDOCK clusters of CRLIIF docking to the HIF-1α PAS-B domain using the Rosetta and **(b)** REST2 derived peptide conformation. A transparent overlay of HIF-1β (green) is included to illustrate the HIF-1β binding site. **(c)** atomistic structures of HADDOCK docked binding poses; the best scoring structure for the cluster that overlapped the HIF-1β binding site using REST2 and **(d)** Rosetta derived starting structures of CRLIIF. **(e)** Representative structures for the top four HADDOCK clusters of CRLIIF docking to the HIF-1α PAS-B domain using the Rosetta and **(f)** REST2 derived peptide conformation. Corresponding HADDOCK scores for the top four clustered states are provided for each independent HADDOCK run using the two starting structures.

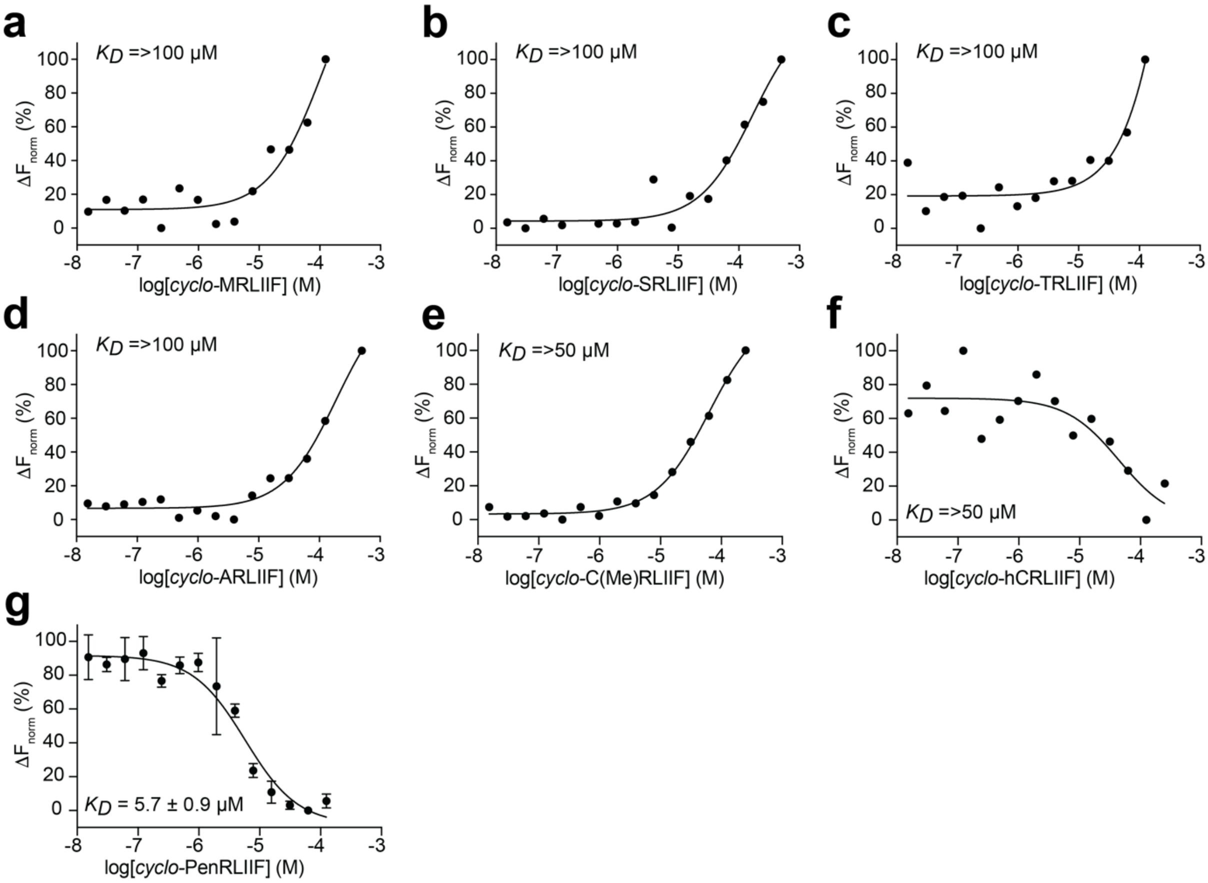

Figure S5. Dose-response curves for C1 substituted derivatives of CRLIIF measure by MST against HIF-1α PAS-B. (a) *cyclo*-MRLIIF, n=1. **(b)** *cyclo*-SRLIIF, **(c)** *cyclo*-TRLIIF, n=1. **(d)** *cyclo*-ARLIIF, n=1. **(e)** *cyclo*-C(Me)RLIIF, n=1. **(f)** *cyclo*-hCRLIIF, n=1. **(g)** *cyclo*-PenRLIIF, data shown as mean ± SEM, n=2.

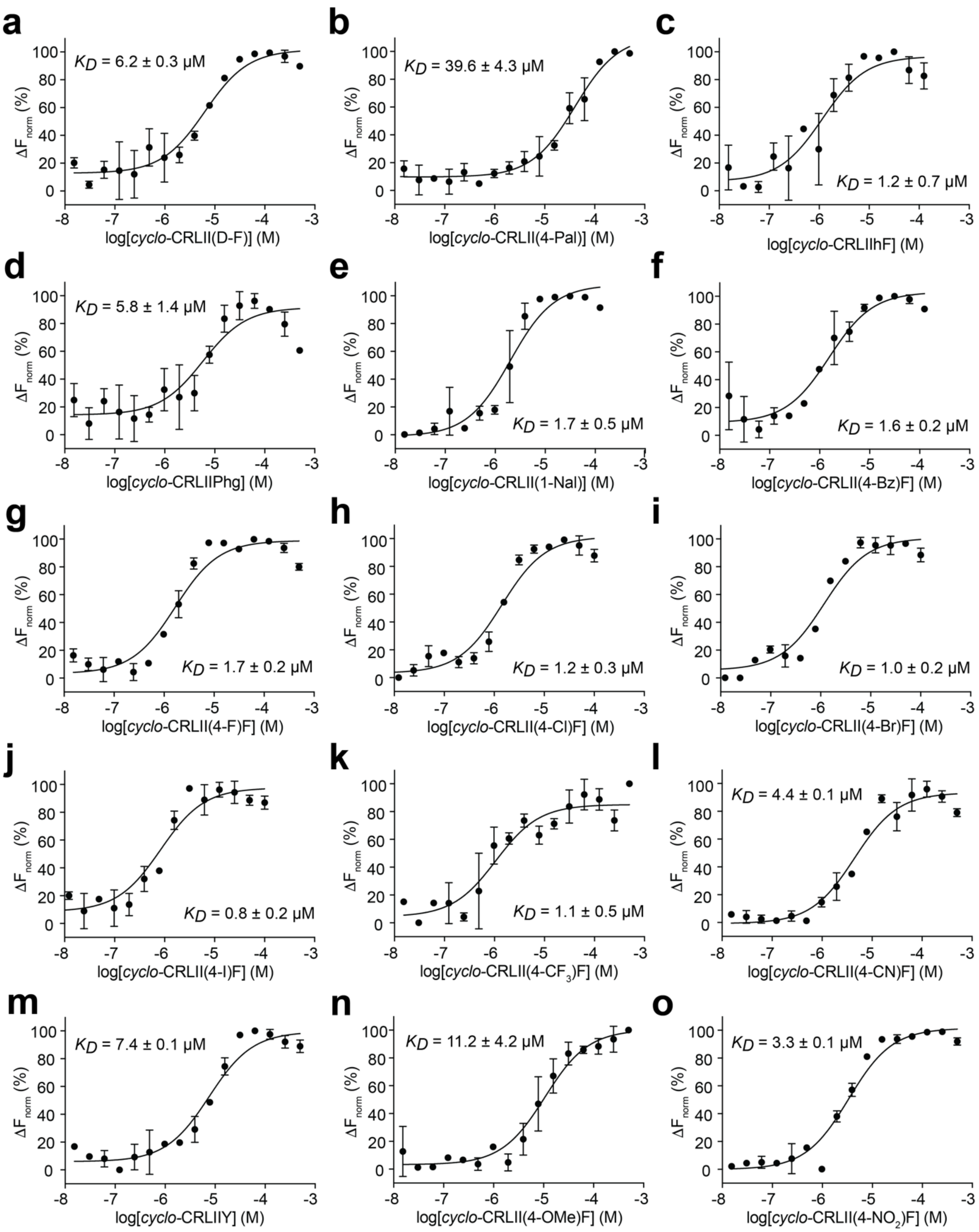

Figure S6. Dose-response curves for F6 substituted derivatives of CRLIIF measure by MST against HIF-1α PAS-B. (a) *cyclo*-CRLII(D-F). **(b)** *cyclo*-CRLII(4-Pal). **(c)** *cyclo*-CRLIIhF. **(d)** *cyclo*-CRLIIPhg. **(e)** *cyclo*-CRLII(1-Nal). **(f)** *cyclo*-CRLII(4-Bz)F. **(g)** *cyclo*-CRLII(4-F)F. **(h)** *cyclo*-CRLII(4-Cl)F. **(i)** *cyclo*-CRLII(4-Br)F. **(j)** *cyclo*-CRLII(4-I)F. **(k)** *cyclo*-CRLII(4-CF_3)F. **(l)** *cyclo*-CRLII(4-CN)F. **(m)** *cyclo*-CRLIIY. **(n)** *cyclo*-CRLII(4-OMe)F. **(o)** *cyclo*-CRLII(4-NO_2)F. All data shown as mean ± SEM, n=2.

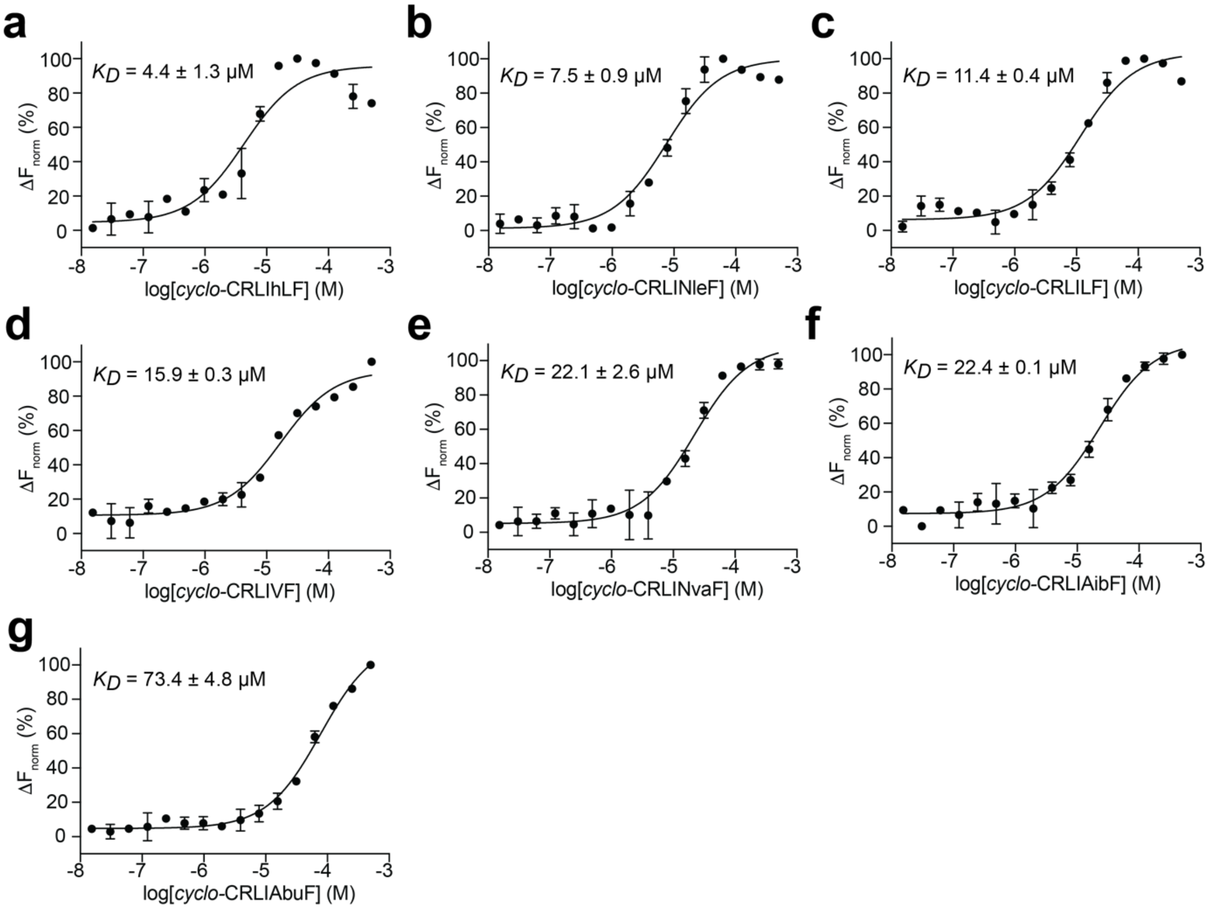

Figure S7. Dose-response curves for I5 substituted derivatives of CRLIIF measure by MST against HIF-1α PAS-B. (a) *cyclo*-CRLIhLF. **(b)** *cyclo*-CRLINleF. **(c)** *cyclo*-CRLILF. **(d)** *cyclo*-CRLIVF. **(e)** *cyclo*-CRLINvaF. **(f)** *cyclo*-CRLIAibF. **(g)** *cyclo*-CRLIAbuF. All data is mean ± SEM, n=2.

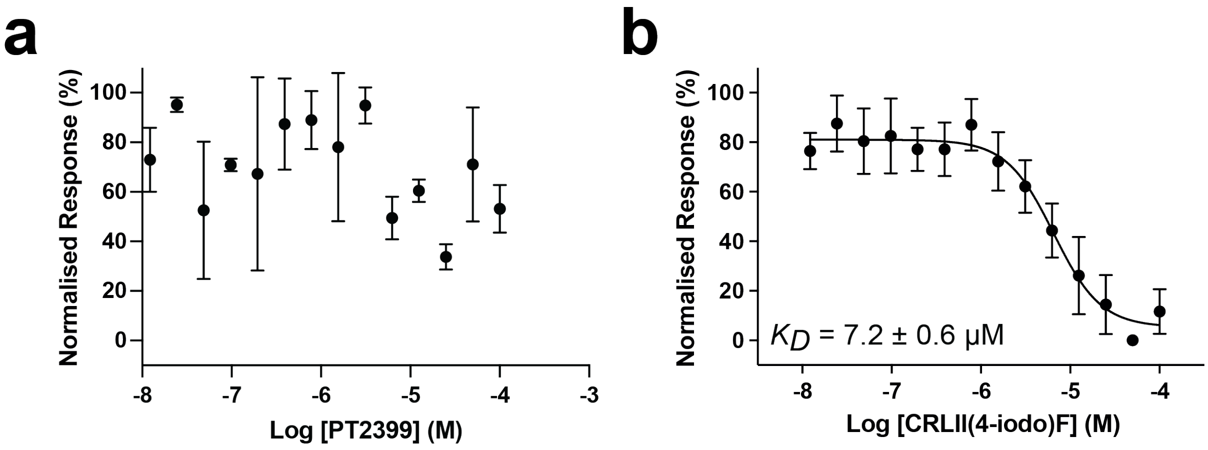

Figure S8. Assessing the binding of PT2399 and *cyclo*-CRLII(4-iodo)F to G323E HIF-2α PAS-B by MST. (a) PT2399. **(b)** *cyclo*-CRLII(4-iodo)F.

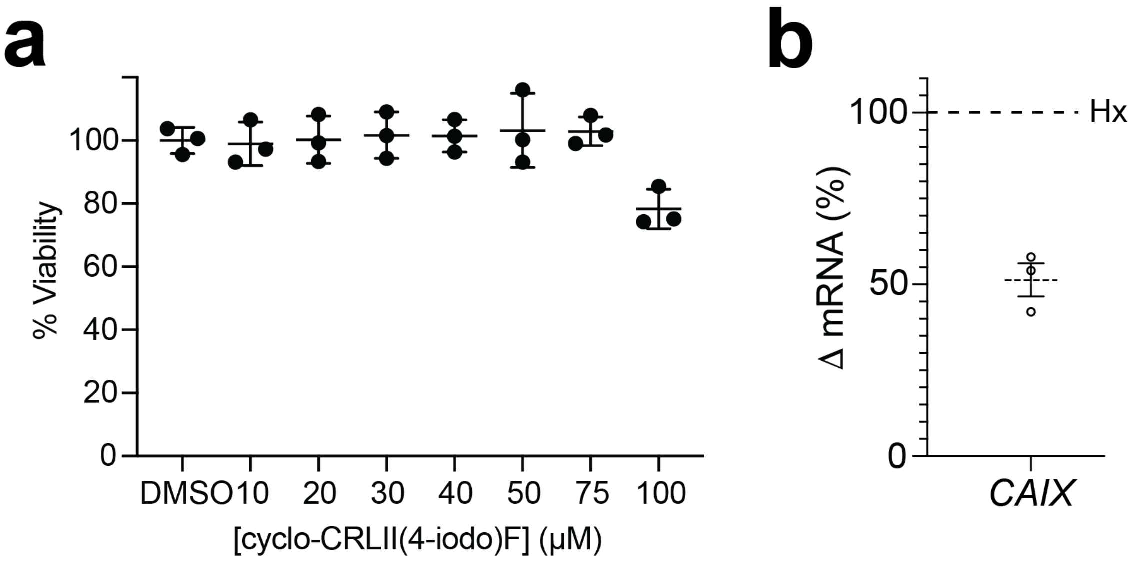

Figure S9. (a) The effect of *cyclo*-CRLII(4-iodo)F on the viability of T-ReX-293 cells. **(b)** The effect of *cyclo*-CRLII(4-iodo)F on the expression of *CAIX* in HeLa cells by qPCR; data is normalized to CAIX levels in hypoxia in DMSO-treated cells (Hx = hypoxia, dotted line). Data is shown as mean (n=3) ± SEM.

Material and Methods

All reagents were purchased from Fisher Scientific or Sigma Aldrich UK unless specified otherwise. Restriction enzymes and microbiology reagents were purchased from New England Biolabs Inc., Fisher Scientific (UK) or Promega (UK) and were used as directed by the manufacturer. Primers and oligonucleotides were synthesised by Integrated DNA Technologies (UK). DNA sequencing was carried out by Eurofins MWG Operon (Germany).

Construction of the RTHS and SICLOPPS library, screening, purification of recombinant HIF proteins, and the PLA was conducted as previously described in Miranda *et al*., J. Am. Chem. Soc., 2013, 135(28); 10418-10425.

All the cells used in this study were cultures in DMEM medium with 10% FBS and incubated in humidified atmosphere with 5% CO_2 unless stated otherwise. For studies in hypoxia, cells were incubated in a Don Whitley DW35 hypoxia incubator at 1% Oxygen; isolation of mRNA and protein for these experiments was carried out in a hypoxic environment.

Protein labelling with MST dye

Protein was buffer exchanged from storage buffer into labelling buffer (0.1 M $NaHCO_3$, pH 8.5) using a PD spintrap G-25 column (GE healthcare). The protein concentration was then adjusted to 20 µM. The commercially sourced MST dye (NanoTemper) was prepared as a 470 µM solution in DMSO, which was then further diluted to 60 µM using labelling buffer. To the protein solution was added an equal volume of dye solution, and the labelling reaction was left for 1 hour in the dark at room temperature. The labelled protein was separated from unreacted dye by elution through a PD minitrap G-25 column (GE healthcare). Labelled protein was then aliquoted and stored at -80 °C. Extent of labelling was measured by the absorbance obtained using the UV-vis mode on a NanoDrop ND-1000 Spectrophotometer (NanoDrop Technologies, USA).

MST

Labelled protein was thawed on ice and diluted to 50 nM in assay buffer containing 50 mM Tris pH 8.0, 150 mM NaCl, 5% (v/v) glycerol, 1 mM TCEP and 0.05% TWEEN-20. The protein solution was then centrifuged at 12000 rpm for 15 minutes at 4 °C and left on ice. Compounds were prepared and serially diluted in DMSO at 10x assay concentration, before diluting to 2x assay concentration in assay buffer. The 2x compound solutions were then mixed 1:1 (v/v) with 50 nM labelled protein solution and left to incubate for 1 hour at room temperature. Solutions were then loaded into Monolith NT.115 Premium Capillaries (NanoTemper) and placed in the sample tray. MST experiments were carried out

using a Monolith NT.115 system (NanoTemper) using 50% LED power and 50% MST power with the temperature set to 25 °C.

Fluorescence Polarisation

Labelled protein and serial dilution samples were prepared as previously described for MST. 25 µL of each dilution was dispensed, in triplicate, into wells in a flat-bottomed, black 384-well microtitre plate (Greiner Bio-One). Polarisation readings were then taken using a Clariostar plate reader (BMG Labtech), using gain and focus settings that were auto-optimised using the supplied software, set to 300 mP units from a control sample.

Solid Phase Peptide Synthesis

Peptides were synthesised by Fmoc solid-phase peptide synthesis using Wang resin preloaded with the first amino acid residue. Coupling and deprotection steps were performed at room temperature in a sintered funnel with agitation through the bottom of the sinter by stream of argon. Coupling solutions were prepared using Fmoc-protected amino acid (3 eq.) and HOBt hydrate (5 eq.) dissolved in DMF, to which DIC (3 eq.) was added. The mixture was stirred for 3 minutes, after which the solution was added to the resin and agitated for 1 h. The resin was washed three times with DMF, then three times DCM and finally three times Et_2O. Fmoc deprotection was carried out by agitating the resin with 20% piperidine in DMF for 20 mins. The resin was washed as before, and successful deprotection checked using the Kaiser test prior to moving on. After deprotection of the final residue, the dry resin was transferred to a 12 mL vial and stirred with TFA/TIS/H2O (95:2.5:2.5) cocktail (10 mL per mmol peptide) for 2.5 h to cleave the peptide. The mixture was filtered through a cotton filter, and the filtrate concentrated in vacuo. Peptide was precipitated from the remaining residue with cold Et_2O. The Et_2O was removed and precipitate triturated a further two times with cold Et_2O, after which the solid was dried on a rotary evaporator. Depending on purity (determined by LCMS), the peptide was either used for subsequent reactions without further purification, or was dissolved in a H_2O:MeCN mixture (1:1) prior to purification by reverse-phase chromatography.

Peptide Cyclisation

Linear peptide, HATU (1.5 eq) and HOAt (3 eq) were dissolved in DMF (1 mL DMF per mg peptide). To the stirring mixture was added DIPEA (5 eq) and the reaction was left to stir at room temperature for 4-18 h. The reaction was monitored by LCMS. After reaction completion, the mixture was concentrated on a rotary evaporator and the remaining residue purified by reverse-phase chromatography if required, otherwise the residue was telescoped to the next reaction.

StBu Deprotection

The peptide and DTT (10 eq) were dissolved in 1 mL DMF. To the mixture was added 1M $(NH_4)_2CO_3$ solution (10 eq) in H_2O. The reaction was stirred at room temperature for 30 mins. The reaction mixture was filtered through a 0.2 µm Teflon syringe filter (Thermo Scientific) and purified directly by reverse-phase preparative HPLC.

2-Chlorotrityl Resin Loading

2-Chlorotrityl resin was swollen in DMF for 15 minutes prior to loading. The DMF was drained and to the resin was added a solution of Fmoc-amino acid (3 eq) and DIPEA (3 eq) in DMF. The resin was agitated for 1 h, after which 10 mL of MeOH was added on top of the reaction mixture and agitated for a further 1 h to cap unreacted sites. The resin was then drained and washed with DMF (3x), DCM (3x) and Et_2O (3x).

The loading was quantified by taking a small quantity of resin, weighed accurately and adding 1 mL 20% piperidine in DMF. The reaction was left for 30 mins before diluting 1:10 with DMF and measuring the absorbance of the dibenzofulvene-piperidine adduct in solution at 301 nm using a NanoDrop ND-1000 Spectrophotometer (NanoDrop Technologies).

Reverse Phase Chromatography

Reverse phase chromatography was performed on a Biotage Isolera One system, using linear gradients of solvents A (0.1% TFA/H2O) and B (0.1% TFA/MeCN). Peptide solutions were loaded into Biotage SNAP Ultra C18 30 g columns dissolved in either a H_2O:MeCN mixture where possible or DMF. Methods were run at 50 mL/min.

Analytical HPLC

Analytical HPLC was performed on an Agilent 1260 Infinity II HPLC system using linear gradients of solvents A (0.1% TFA/H2O) and B (0.1% TFA/MeCN). Peptides were eluted through an Agilent Poroshell 120 EC-C18 column (2.7 µm particle size, 3.0 × 100 mm) at 0.625 mL/min flow rate. Samples were injected as a 5% MeCN/H2O solution. Samples were detected using UV absorbance at 220 nm and 280 nm.

Preparative HPLC

Preparative HPLC was performed on a Waters 1525 HPLC system using linear gradients of solvents A (0.1% TFA/H_2O) and B (0.1% TFA/MeCN). Peptides were purified by preparative HPLC with a Waters Atlantis T3 column (5.0 µm particle size, 19 × 100 mm) at 17 mL/min flow rate.

Computational Chemistry

CRLIIF binding to HIF-1α was modelled using replica exchange with solute tempering 2 (REST2) in Gromacs 5.1.4 patched with Plumed 2.3.3 with the Amber14SB forcefield.[1]. The starting conformation of CRLIIF was created in XLeap then solvated in a dodecahedral box of TIP3P waters[2] using 1.0 nm radius between box edge and solute. Na^+ and Cl^- ions were added to a final concentration of 0.15 M and to neutralise the system. The system was subject to energy minimisation using steepest descent for 50,000 steps and additional minimisation using conjugate gradient for an additional 50,000 steps. Minimisations used PME for long-range electrostatics with a cut-off of 1.0 nm and long-range van der Waals cut-off of 1.0 nm. The system was equilibrated for 1 ns in NVT using the Nosé-Hoover thermostat at 300 K and 2.0 ps time constant alongside a 2 fs timestep using the leap-frog integrator. Hydrogen bonds were constrained using the LINCS algorithm. The CRLIIF peptide system was then simulated for 500 ns in NVT using REST2 across 16 replicas distributed between 300 K to 4000 K effective temperatures. Scaling was applied to the whole peptide. The resulting trajectory was then clustered by backbone dihedral angles using Dash[3] and a representative CRLIIF structure was selected from the most observed Dash cluster state. The CRLIIF structure was then placed in a simulation box containing the PAS-B domain of HIF-1α derived from the 4ZPR PDB structure and minimised as before. The structure was equilibrated in NVT and NPT for 100 ps using the v-rescaling thermostat[4] with 0.1 ps time constant and Parrinello-Rahman barostat[5] with 2.0 ps time constant. REST2 was performed with simulation parameters as described before for 200 ns with 16 replicas distributed between 300 K to 500 K. Scaling was only applied to the HIF-1α protein to encourage protein surface exploration of the CRLIIF peptide. The trajectory was then clustered using gmx cluster with a 0.2 nm RMSD cut-off. Interactions were determined for Cys1 3.2 Å distance cut-off respectively between the SG atom and all other heavy atoms of the system. Interactions were determined for Ile5 3.8 Å distance cut-off respectively between CD1 all other heavy atoms of the system. All distances were measured using MDAnalysis distance_array function.

Docking of CRLIIF to HIF-1α was performed using HADDOCK v2.4[6]. Two CRLIIF starting structures were generated, one using Rosetta 2021.16 simple_cycpep_predict[7] in which 8000 structures generation attempts were performed of which 3909 conformations were accepted using 25,000 cyclisation repeats per structure. Conformations were scored according to the rosetta scoring function whereby the best scoring structure was taken. The second structure was taken from the most frequently sampled gmx cluster conformation. Both structures were used for docking in independent docking runs to HIF-1α. The HIF-1α structure was created using ColabFold v1.5.3[8, 9] with the 4ZPR PDB crystal structure as a template.[10]. Five structures were generated and the model with

highest pLDDT score was relaxes with the inbuilt amber energy minimisation and used for subsequent docking steps. HADDOCK docking was performed by assigning the HIF-1α PAS-B domain (residues 214-335) and CRLIIF residues 1, 5 and 6 as active residue binders and clustered using the inbuilt RMSD clustering algorithm.

Cellular Thermal Shift Assay

Pancreatic cancer cells (PANC-1) were plated in a T75 flask at 80% confluency overnight under normoxic conditions. The cells were then dosed with compound and incubated for 18 hours under hypoxic conditions (1% O2, 94% N2, 5% CO2, 80% Humidity). The cells were subsequently washed with PBS and divided into several aliquots, each of which was exposed to different temperatures (45°C, 48°C, 51°C, and 54°C) for 30 seconds on a thermal cycler (Bio-Rad). Following the temperature exposure, the cells were lysed, and total cell protein was collected by cold centrifugation at 14,000g for 20 minutes. The concentration of the extracted proteins was measured using the BCA Protein Assay Kit (Sigma) following the manufacturer's instructions. The extracted proteins were then denatured by mixing with a half volume of 2X SDS loading buffer and heated at 70°C for 10 minutes. The denatured extracted proteins were loaded onto a 10% SDS PAGE gel and transferred onto a nitrocellulose membrane. The membrane was incubated with the HIF-1α primary antibody, and the protein bands were visualized using the Amersham ECL chemiluminescence reagent (Sigma).

Cytotoxicity Assay

The CytoTox-GloTM Cytotoxicity assay (Promega) was used to determine the cytotoxicity level of compound following the manufacturer's instructions. The cells were plated on a 96-well plate at 80% confluency overnight under normoxic conditions. Subsequently, the cells were dosed with various concentrations of compound and incubated for 18 hours under hypoxic conditions. The CytoTox-Glo reagent was added to the cells to determine the rate of viable cells in each treatment using a microplate reader.

Generation of HRE-eYFP integrated cell line

The Flp-In System (Fisher) was used to generate a constitutive expression cell line that expresses eYFP under the control of the VEGF HRE. The coding sequence of the yellow fluorescent protein (eYFP) was cloned downstream of the VEGF HRE sequence in the pcDNA5 plasmid, such that the entire cassette was under the regulation of a Tet-On gene expression system. The plasmid was transfected into a human embryonic kidney cell line (Flp-In™-293) and integrated using the manufacturer’s protocol.

Activity Assessment using Flp-In-HRE-eYFP cell line

The Flp-In-HRE-eYFP cells were plated on 24-well plates at 80% confluency and incubated overnight under normoxic conditions. The cells were then treated with different concentrations of compound and incubated for 18 hours under hypoxic conditions. After treatment, the cells were washed with PBS and lysed to extract the total cellular protein. The cell extract was cold centrifuged for 20 minutes at 14,000 RPM. An equal amount of the supernatant from each treatment was plated on a 96-well plate, and the emission of eYFP was measured using a microplate reader.

Realtime PCR (qPCR) Assay

Cells were plated on 6cm dishes at 80% confluency and incubated overnight under normoxic conditions. The cells were then treated with compound and incubated for 18 hours in hypoxic conditions. After treatment, the cells were washed with PBS and lysed under hypoxic conditions. The total RNA was extracted from the cells using a commercial RNA extraction kit (Fisher Scientific) following the manufacturer's instructions. A commercial reverse transcription kit (Promega) was used to generate cDNA. The MyTaq™ Mix master mix and appropriate primers targeting *CAIX* and *VEGF* were used to perform the qPCR reaction using a qPCR machine (Bio-Rad).

Compound Data

cyclo-CKLIIF

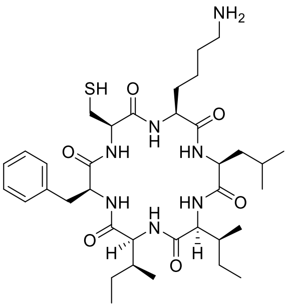

The linear peptide IIFC(StBu)K(Cbz)L was synthesised from Fmoc-Leu-Wang resin (Novabiochem) on a 0.2 mmol scale following the general procedure above, using Fmoc-Lys(Cbz)-OH. After precipitation from ether, the peptide was purified by RPC and lyophilised. The white solid was then dissolved in DMF and cyclised following the general procedure above. The peptide was concentrated, deprotected following the general procedure above , purified and lyophilised. The subsequent white solid was left stirring in 2 mL TFA over 3 days at room temperature, after which the product was purified by RP-HPLC and lyophilised to obtain the product as a white solid (10 mg, 7% overall yield). LCMS R_t: 1.71 min, Analytical HPLC R_t: 11.283 min (91% purity), LRMS *m/z* (ESI$^+$): 718.6 [M+H] (100%), 360.0 [M+2H] (85%), HRMS *m/z* (ESI$^+$): calc. for $C_{36}H_{60}N_7O_6S^+$ ([M+H$^+$]) 718.4320, found 718.4326.

a)

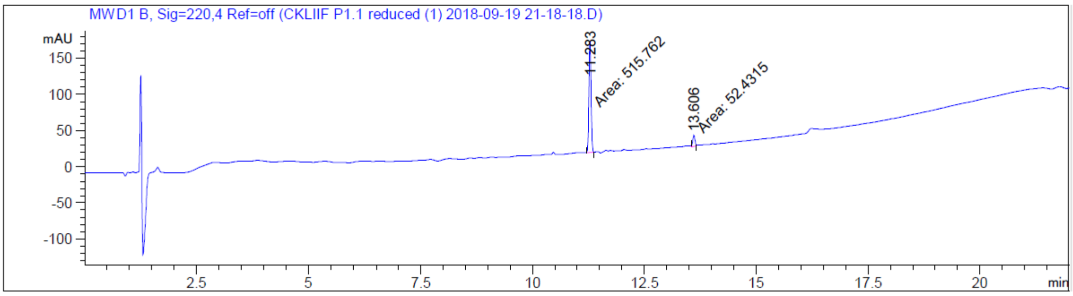

b)

Peak #	RetTime [min]	Type	Width [min]	Area [mAU*s]	Height [mAU]	Area %
1	11.283	MM	0.0557	515.76202	154.27473	90.7722
2	13.606	MM	0.0571	52.43149	15.29849	9.2278

c)

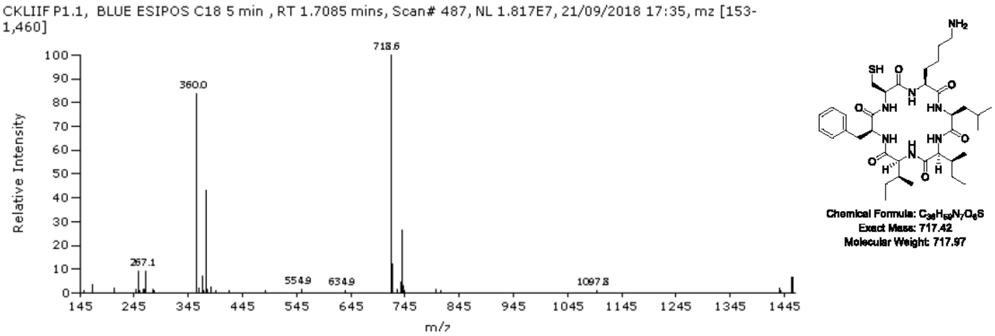

<u>*cyclo*-CRLLIF</u>

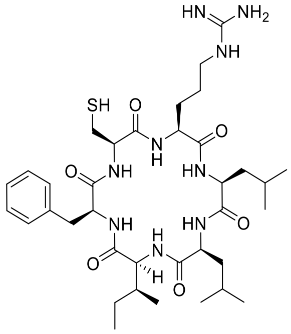

The linear peptide LIFC(StBu)RL was synthesised from Fmoc-Leu-Wang resin (Novabiochem) on a 0.2 mmol scale following the general procedure above. After precipitation from ether, the peptide was dissolved in DMF and cyclised following the general procedure above. The peptide was concentrated, and then deprotected following the general procedure above. The product was purified by RP-HPLC and lyophilised to obtain the product as a white solid (83 mg, 56% overall yield). LCMS R_t: 1.79 min, Analytical HPLC R_t: 12.298 min (95% purity), LRMS *m/z* (ESI$^+$): 834.7 [M+H] (100%), 418.1 [M+2H] (85%), HRMS *m/z* (ESI$^+$): calc. for $C_{36}H_{60}N_9O_6S^+$ ([M+H$^+$]) 746.4382, found 746.4366.

a)

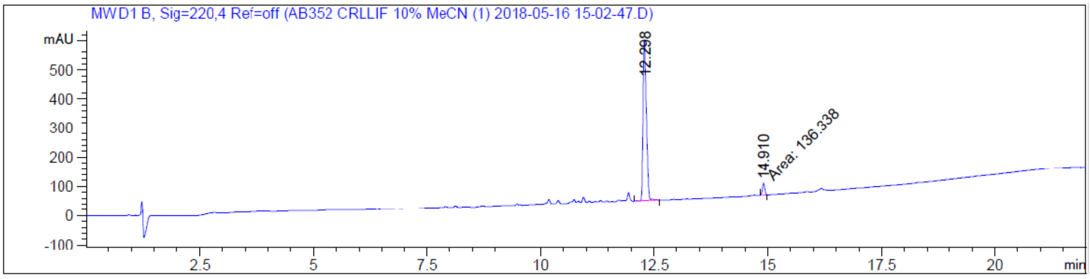

b)

Peak #	RetTime [min]	Type	Width [min]	Area [mAU*s]	Height [mAU]	Area %
1	12.298	BB	0.1084	2764.89526	471.84857	95.3007
2	14.910	MM	0.0575	136.33759	39.51272	4.6993

c)

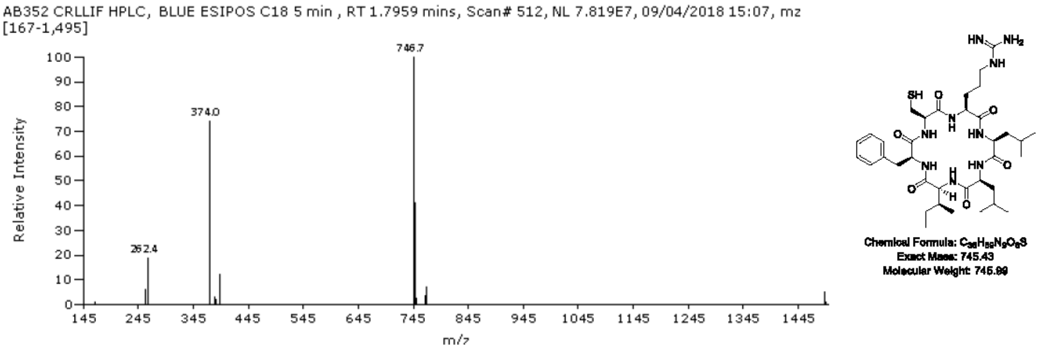

<u>*cyclo*-CRVIIF</u>

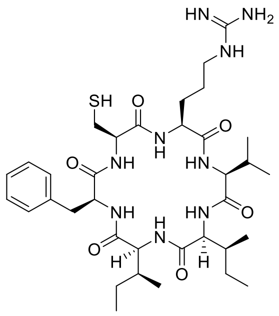

The linear peptide IIFC(StBu)RV was synthesised from Fmoc-Val-Wang resin (Novabiochem) on a 0.2 mmol scale following the general procedure above. After precipitation from ether, the peptide was dissolved in DMF and cyclised following the general procedure above. The peptide was concentrated, and then deprotected following the general procedure above. The product was purified by RP-HPLC and lyophilised to obtain the product as a white solid (18 mg, 12% overall yield). LCMS R_t: 1.75 min, Analytical HPLC R_t: 11.458 min (96% purity), LRMS *m/z* (ESI$^+$): 732.7 [M+H] (100%), 366.9 [M+2H] (85%), HRMS *m/z* (ESI$^+$): calc. for $C_{35}H_{58}N_9O_6S^+$ ([M+H$^+$]) 732.4225, found 732.4217.

a)

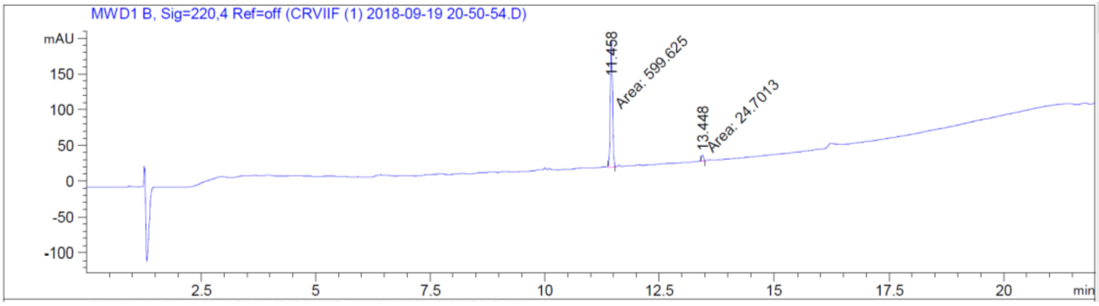

b)

Peak #	RetTime [min]	Type	Width [min]	Area [mAU*s]	Height [mAU]	Area %
1	11.458	MM	0.0561	599.62543	178.12672	96.0435
2	13.448	MM	0.0511	24.70134	8.05775	3.9565

c)

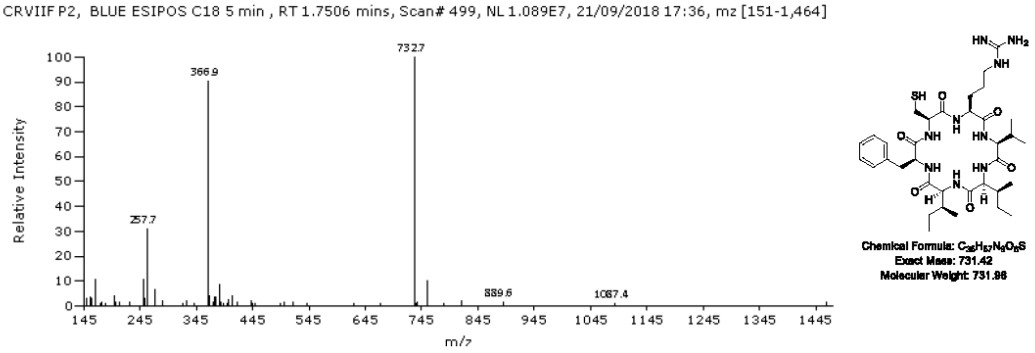

cyclo-CRLIIF

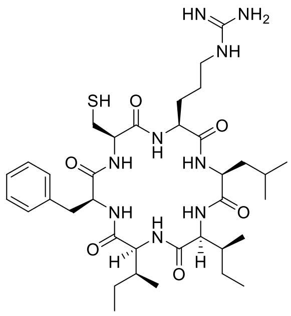

The linear peptide IIFC(StBu)RL was synthesised from Fmoc-Leu-Wang resin (Novabiochem) on a 0.2 mmol scale following the general procedure above. After precipitation from ether, the peptide was dissolved in DMF and cyclised following the general procedure above. The peptide was concentrated, and then deprotected following the general procedure above. The product was purified by RP-HPLC and lyophilised to obtain the product as a white solid (60 mg, 40% overall yield). LCMS R_t: 1.88 min, Analytical HPLC R_t: 12.499 min (99% purity), LRMS *m/z* (ESI$^+$): 746.6 [M+H] (100%), 374.0 [M+2H] (90%), HRMS *m/z* (ESI$^+$): calc. for $C_{36}H_{60}N_9O_6S^+$ ([M+H$^+$]) 746.4382, found 746.4380.

a)

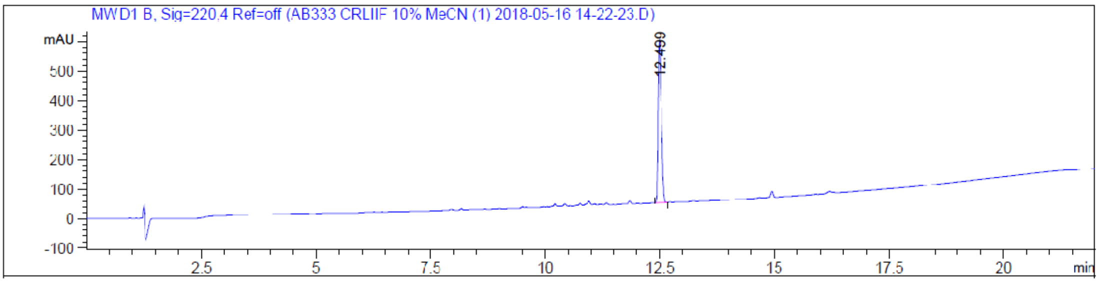

b)

Peak #	RetTime [min]	Type	Width [min]	Area [mAU*s]	Height [mAU]	Area %
1	12.499	BB	0.0924	2772.46265	535.12531	100.0000

c)

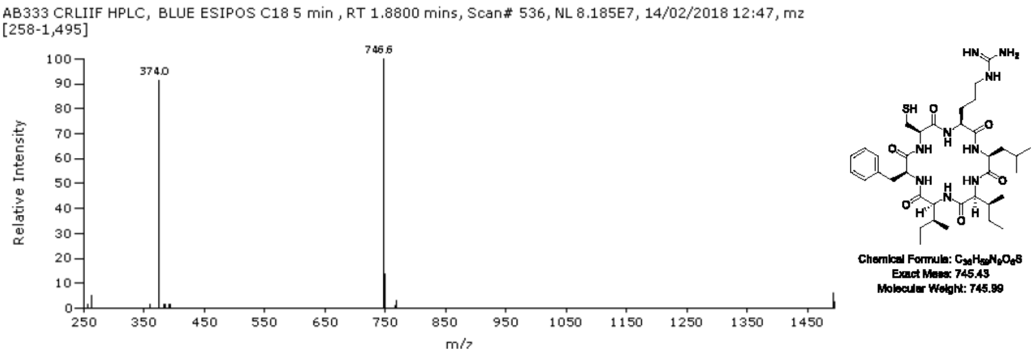

cyclo-CKLLIF

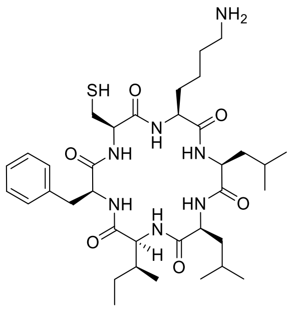

The linear peptide IIFC(StBu)K(Cbz)L was synthesised from Fmoc-Leu-Wang resin (Novabiochem) on a 0.2 mmol scale following the general procedure above using Fmoc-Lys(Cbz)-OH instead of Fmoc-Lys(Boc)-OH. After precipitation from ether, the peptide was purified by RPC and lyophilised. The white solid was then dissolved in DMF and cyclised following the general procedure above. The peptide was concentrated, deprotected following the general procedure above, purified by RPC and lyophilised. The subsequent white solid was left stirring in 2 mL TFA over 3 days at room temperature, after which the product was purified by RP-HPLC and lyophilised to obtain the product as a white solid (23 mg, 16% overall yield). LCMS R_t: 1.89 min, Analytical HPLC R_t: 12.115 min (97% purity), LRMS *m/z* (ESI^+): 719.1 [M+H] (100%), 360.1 [M+2H] (39%), HRMS *m/z* (ESI^+): calc. for $C_{36}H_{60}N_7O_6S^+$ ($[M+H^+]$) 718.4320, found 718.4325.

a)

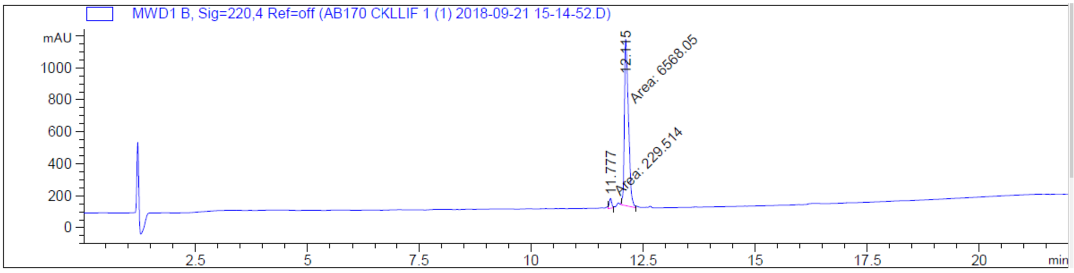

b)

Peak #	RetTime [min]	Type	Width [min]	Area [mAU*s]	Height [mAU]	Area %
1	11.777	MM	0.0609	229.51402	62.82053	3.3764
2	12.115	MM	0.1046	6568.04639	1046.96582	96.6236

c)

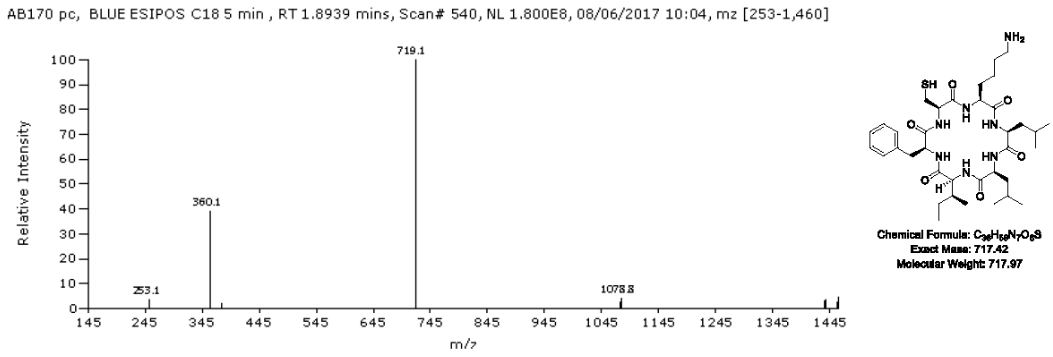

cyclo-CRLAIF

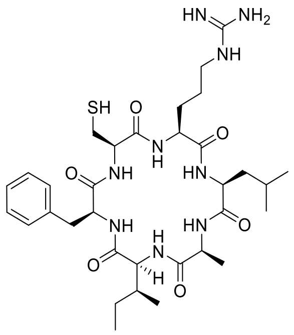

The linear peptide AIFC(StBu)RL was synthesised from Fmoc-Leu-Wang resin (Novabiochem) on a 0.1 mmol scale following the general procedure above. After precipitation from ether, the peptide was dissolved in DMF and cyclised following the general procedure above. The peptide was concentrated, and then deprotected following the general procedure above. The product was purified by RP-HPLC and lyophilised to obtain the product as a white solid (23 mg, 33% overall yield). LCMS R_t: 1.69 min, Analytical HPLC R_t: 10.659 min (99% purity), LRMS *m/z* (ESI^+): 704.7 [M+H] (100%), 353.0 [M+2H] (75%), HRMS *m/z* (ESI^+): calc. for $C_{33}H_{54}N_9O_6S^+$ ([M+H$^+$]) 704.3912, found 704.3905.

a)

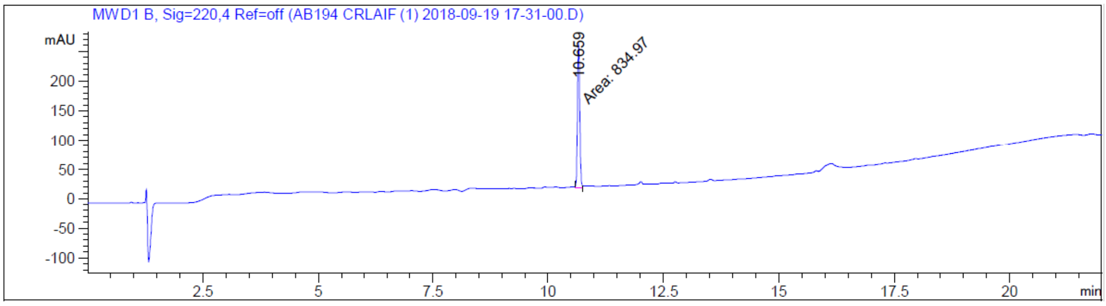

b)

Peak #	RetTime [min]	Type	Width [min]	Area [mAU*s]	Height [mAU]	Area %
1	10.659	MM	0.0564	834.96960	246.60391	100.0000

c)

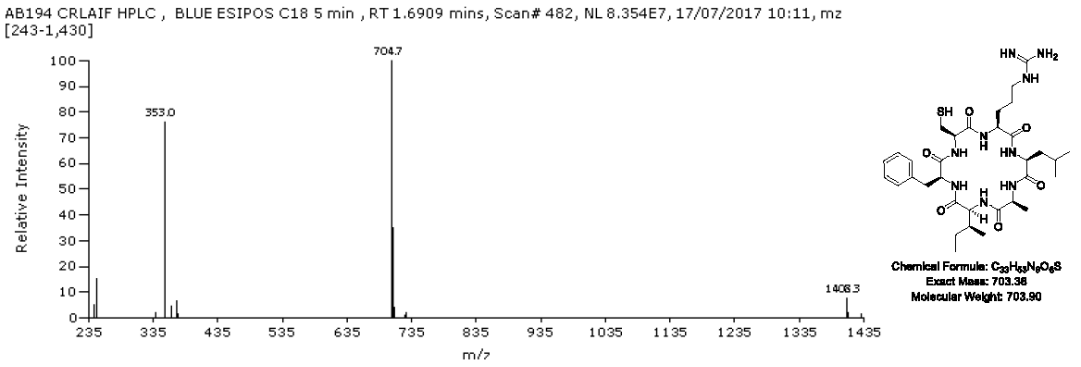

cyclo-CRLIAF

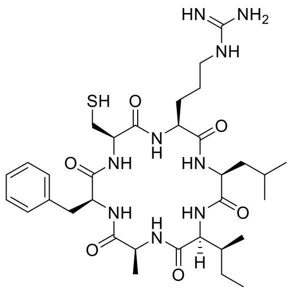

The linear peptide IAFC(StBu)RL was synthesised from Fmoc-Leu-Wang resin (Novabiochem) on a 0.1 mmol scale following the general procedure above. After precipitation from ether, the peptide was dissolved in DMF and cyclised following the general procedure above. The peptide was concentrated, and then deprotected following the general procedure above. The product was purified by RP-HPLC and lyophilised to obtain the product as a white solid (10 mg, 14% overall yield). LCMS R_t: 1.74 min, Analytical HPLC R_t: 10.937 min (93% purity), LRMS *m/z* (ESI^+): 353.1 [M+2H] (100%), 704.7 [M+H] (60%), HRMS *m/z* (ESI^+): calc. for $C_{33}H_{54}N_9O_6S^+$ ($[M+H^+]$) 704.3912, found 704.3907.

a)

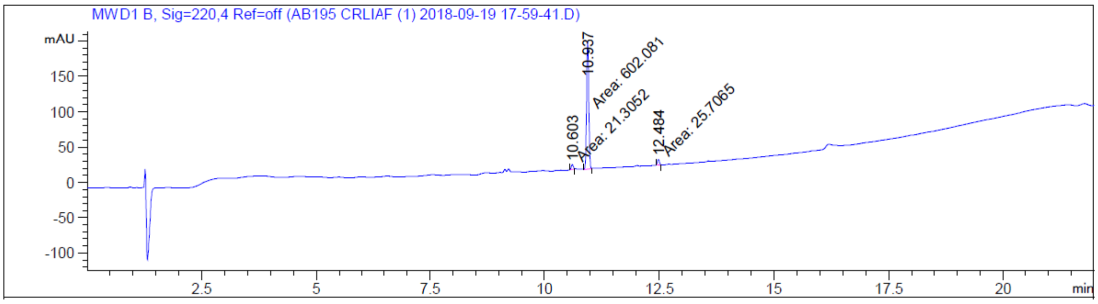

b)

Peak #	RetTime [min]	Type	Width [min]	Area [mAU*s]	Height [mAU]	Area %
1	10.603	MM	0.0509	21.30521	6.97623	3.2823
2	10.937	MM	0.0548	602.08051	182.94934	92.7573
3	12.484	MM	0.0517	25.70645	8.28711	3.9604

c)

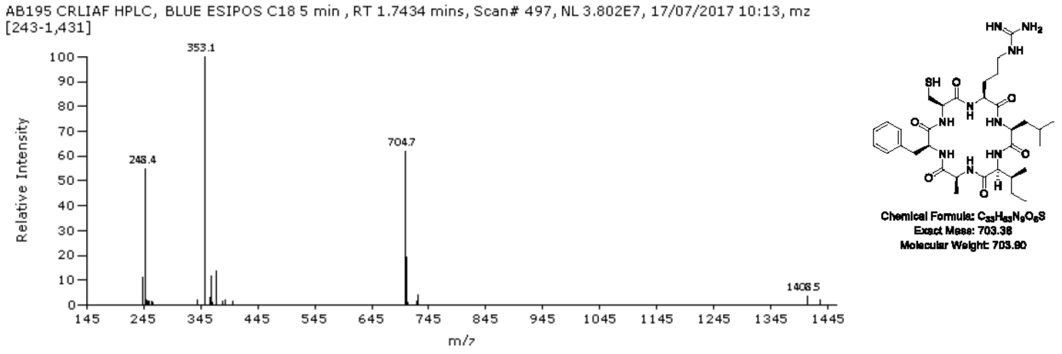

<u>*cyclo*-CRLIIA</u>

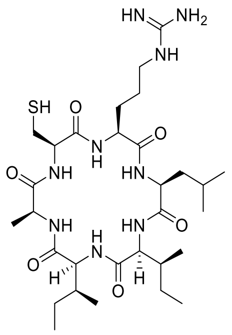

The linear peptide IIAC(StBu)RL was synthesised from Fmoc-Leu-Wang resin (Novabiochem) on a 0.1 mmol scale following the general procedure above. After precipitation from ether, the peptide was dissolved in DMF and cyclised following the general procedure above. The peptide was concentrated, and then deprotected following the general procedure above. The product was purified by RP-HPLC and lyophilised to obtain **8** as a white solid (31 mg, 46% overall yield). LCMS R_t: 1.71 min, Analytical HPLC R_t: 10.493 min (86% purity), LRMS *m/z* (ESI$^+$): 336.1 [M+2H] (100%), 237.1 [M+2H+Na] (70%), 670.7 [M+H] (50%), HRMS *m/z* (ESI$^+$): calc. for $C_{30}H_{56}N_9O_6S^+$ ([M+H$^+$]) 670.4069, found 670.4065.

a)

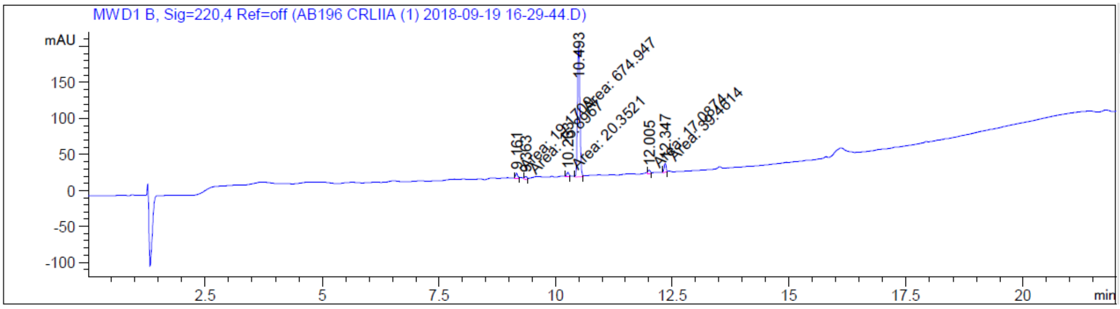

b)

Peak #	RetTime [min]	Type	Width [min]	Area [mAU*s]	Height [mAU]	Area %
1	9.161	MM	0.0524	19.17089	6.09556	2.4362
2	9.363	MM	0.0628	15.89673	4.21611	2.0201
3	10.263	MM	0.0573	20.35209	5.91810	2.5863
4	10.493	MM	0.0599	674.94666	187.81445	85.7712
5	12.005	MM	0.0602	17.08745	4.72755	2.1714
6	12.347	MM	0.0528	39.46145	12.46525	5.0147

c)

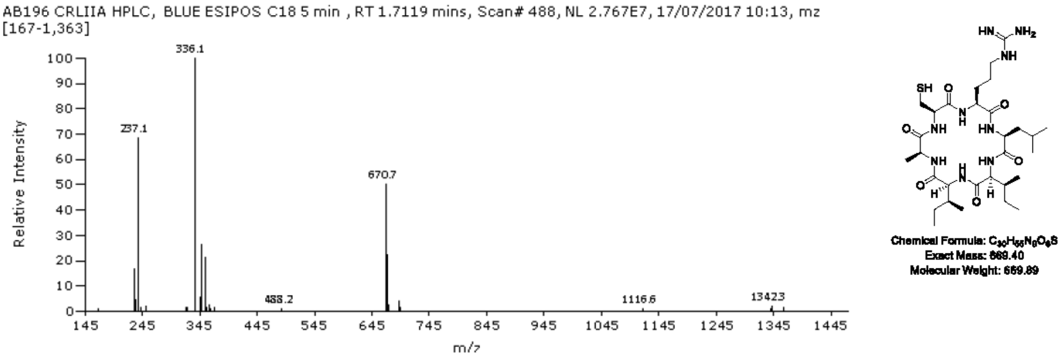

cyclo-ARLIIF

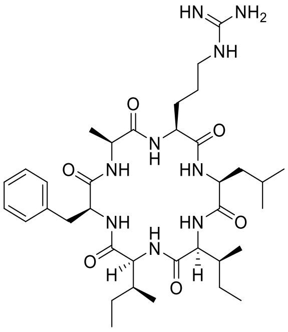

The linear peptide IIFARL was synthesised from Fmoc-Leu-Wang resin (Novabiochem) on a 0.1 mmol scale following the general procedure above. After precipitation from ether, the peptide was dissolved in DMF and cyclised following the general procedure above. The product was purified by RP-HPLC and lyophilised to obtain the product as a white solid (33 mg, 46% overall yield). LCMS R_t: 1.91 min, Analytical HPLC R_t: 12.120 min (94% purity) LRMS *m/z* (ESI$^+$): 358.2 [M+2H] (100%), 714.8 [M+H] (80%), HRMS *m/z* (ESI$^+$): calc. for $C_{36}H_{60}N_9O_6^+$ ([M+H$^+$]) 714.4661, found 714.4657.

a)
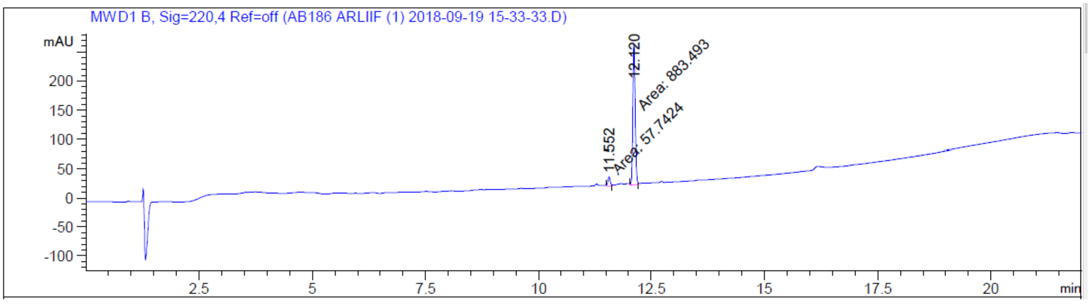

b)

Peak #	RetTime [min]	Type	Width [min]	Area [mAU*s]	Height [mAU]	Area %
1	11.552	MM	0.0602	57.74236	15.98884	6.1347
2	12.120	MM	0.0609	883.49316	241.76889	93.8653

c)
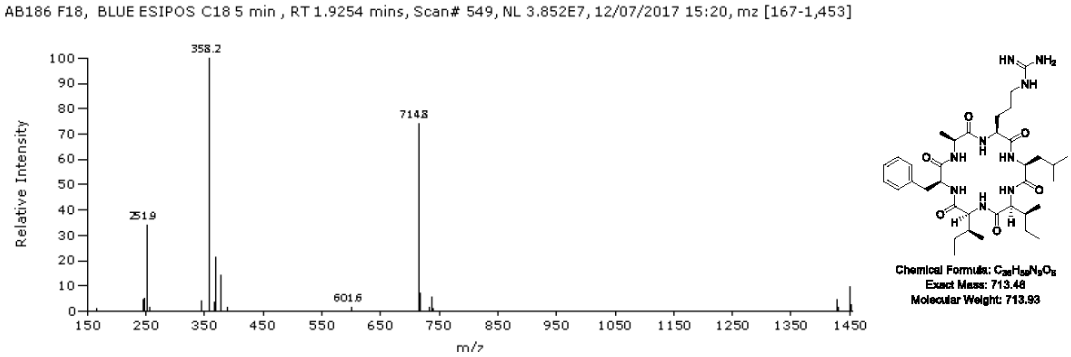

cyclo-CALIIF

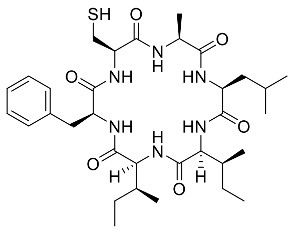

The linear peptide IIAC(StBu)RL was synthesised from Fmoc-Leu-Wang resin (Novabiochem) on a 0.2 mmol scale following the general procedure above. After precipitation from ether, the peptide was dissolved in DMF and cyclised following the general procedure above. The peptide was concentrated, and then deprotected following the general procedure above. The product was purified by RP-HPLC and lyophilised to obtain the product as a white solid (13 mg, 10% overall yield). LCMS R_t: 2.25 min, Analytical HPLC R_t: 14.566 min (90% purity), LRMS *m/z* (ESI$^+$): 350.4 [M+H+K] (100%), 683.6 [M+Na] (85%), 661.5 [M+H] (30%), HRMS *m/z* (ESI$^+$): calc. for $C_{33}H_{52}N_6O_6SNa^+$ ([M+Na$^+$]) 683.3561, found 683.3551.

a)

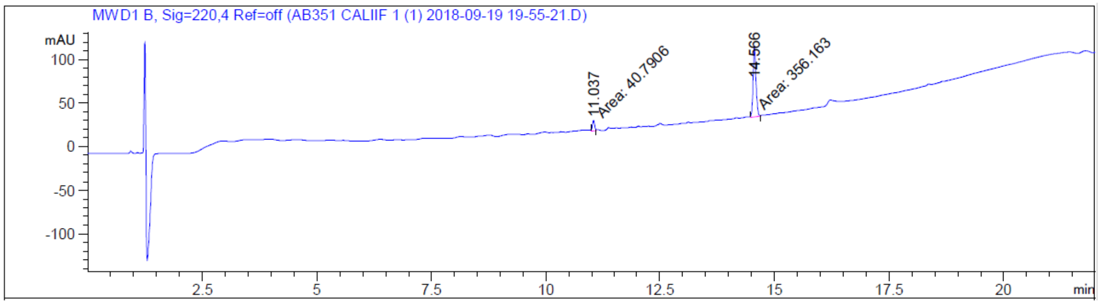

b)

Peak #	RetTime [min]	Type	Width [min]	Area [mAU*s]	Height [mAU]	Area %
1	11.037	MM	0.0570	40.79062	11.91971	10.2759
2	14.566	MM	0.0722	356.16293	82.18568	89.7241

c)

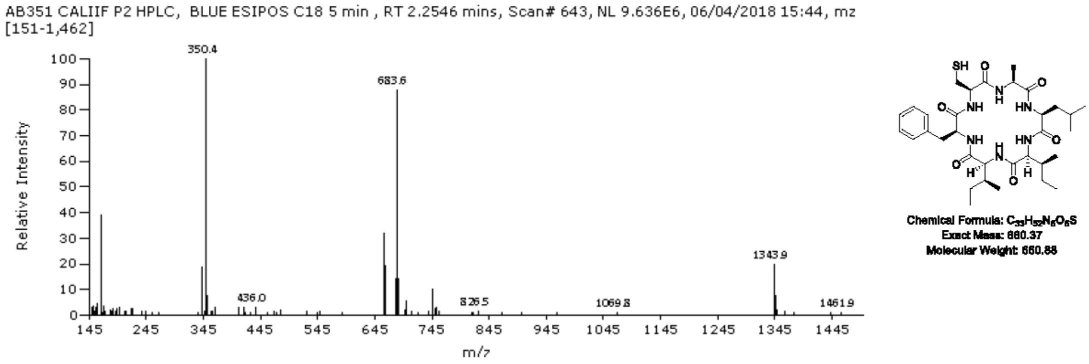

cyclo-CRAIIF

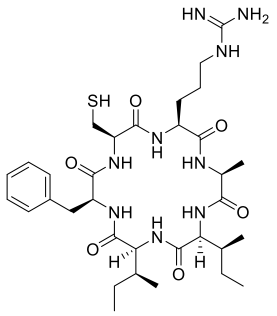

The linear peptide IIAC(StBu)RL was synthesised from Fmoc-Leu-Wang resin (Novabiochem) on a 0.1 mmol scale following the general procedure above. After precipitation from ether, the peptide was dissolved in DMF and cyclised following the general procedure above. The peptide was concentrated, and then deprotected following the general procedure above. The product was purified by RP-HPLC and lyophilised to obtain the product as a white solid (7 mg, 10% overall yield). LCMS R_t: 1.76 min, Analytical HPLC R_t: 11.081 min (90% purity), LRMS *m/z* (ESI$^+$): 353.1 [M+2H] (100%), 704.7 [M+H] (60%), HRMS *m/z* (ESI$^+$): calc. for $C_{33}H_{54}N_9O_6S^+$ ([M+H$^+$]) 704.3912, found 704.3905.

a)

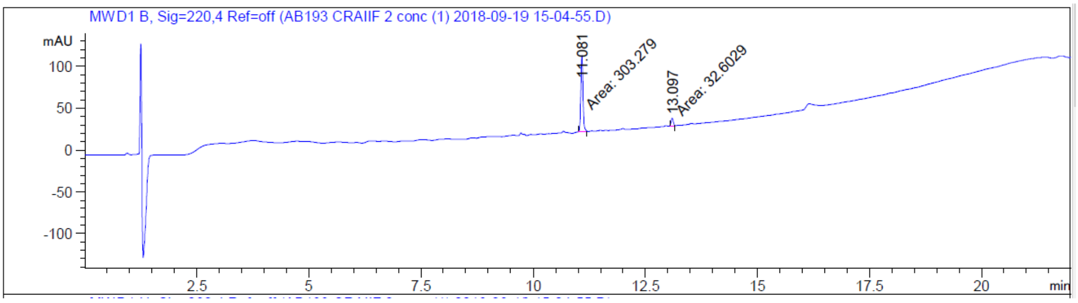

b)

Peak #	RetTime [min]	Type	Width [min]	Area [mAU*s]	Height [mAU]	Area %
1	11.081	MM	0.0553	303.27866	91.35619	90.2933
2	13.097	MM	0.0565	32.60291	9.62247	9.7067

c)

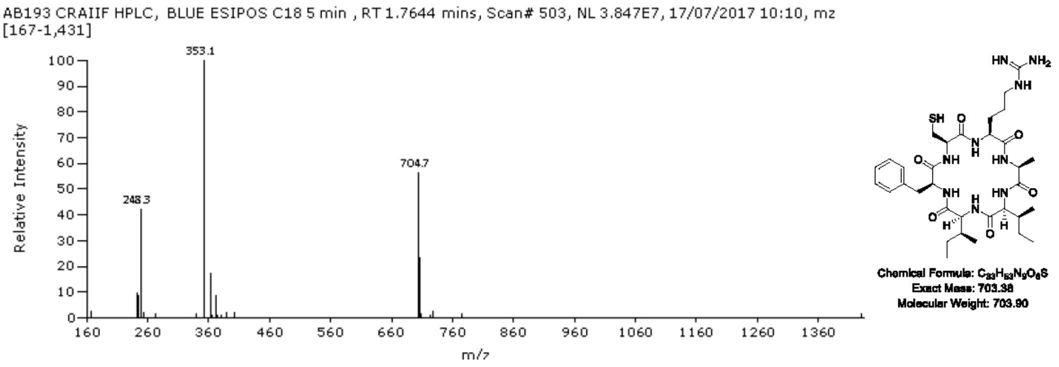

<u>*cyclo*-PenRLIIF</u>

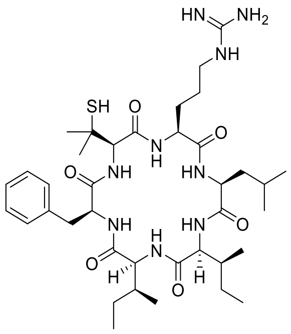

The peptide F-Pen-RLII was synthesised from Fmoc-Ile-Wang resin (Novabiochem) on a 0.1 mmol scale following the general procedure above 0, using Fmoc-Pen(Trt)-OH for the penicillamine coupling. Following precipitation from ether, the peptide as the TFA salt was dried and isolated as an off-white solid (72 mg, 79%) and used without further purification. LCMS R_t: 1.84 min, LRMS *m/z* (ESI^+): 397.2 [M+2H] (100%), 792.9 [M+H] (10%).

The product of the above reaction (72 mg, 0.079 mmol) was dissolved in MeOH (1 mL). To the stirring mixture was added dropwise a solution of aldrithiol-2 (35 mg, 0.159 mmol, 2 eq) in MeOH (1 mL). The reaction was left to stir at room temperature for 30 min after which no starting material was detected by LCMS. The reaction mixture was concentrated to dryness on a rotary evaporator, and to the residue was added Et_2O (2 mL). The yellow ether solution was removed, and the precipitate washed a further two times with Et_2O. The solid product was dried and isolated as an off-white solid (70 mg, 98%) and used without further purification. LCMS R_t: 1.94 min, LRMS *m/z* (ESI^+): 451.8 [M+2H] (100%).

The product of the above reaction (70 mg, 0.078 mmol), was mixed with HATU (41 mg), HOAt (25 mg) and DIPEA (47 µL) in DMF (90 mL) following the general procedure above. The product was purified by RP-chromatography and lyophilised to obtain the product as a white solid (22 mg, 32%). LCMS R_t: 2.07, LRMS *m/z* (ESI^+) 442.6 [M+2H] (100%), 884.0 [M+H] (10%).

The cyclic peptide was synthesised from the product of the above reaction (22 mg, 0.025 mmol), DTT (77 mg) and 1M aq. $(NH_4)_2CO_3$ (0.5 mL) in DMF (0.5 mL) following the general procedure above. The product was purified by RP-HPLC and lyophilised to obtain the product as a white solid (15 mg, 78%). LCMS R_t: 1.95 min, Analytical HPLC R_t: 13.384 (81% purity) LRMS *m/z* (ESI^+): 388.1 [M+2H] (100%), 774.9 [M+H] (55%), HRMS *m/z* (ESI^+): calc. for $C_{38}H_{64}N_9O_6S^+$ ([M+H^+]) 774.4695, found 774.4704.

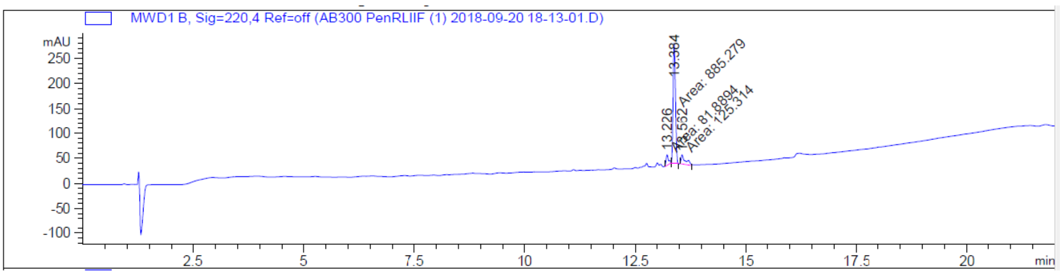

b)

Peak #	RetTime [min]	Type	Width [min]	Area [mAU*s]	Height [mAU]	Area %
1	13.226	MM	0.0634	81.88937	21.54298	7.4957
2	13.384	MM	0.0611	885.27917	241.54636	81.0337
3	13.562	MM	0.1143	125.31419	18.27048	11.4706

c)

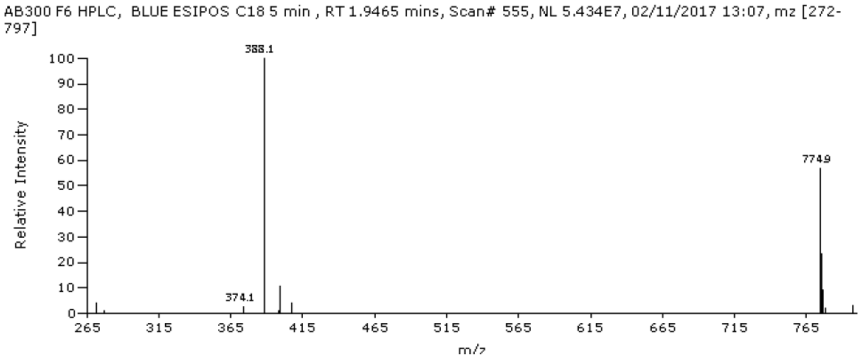

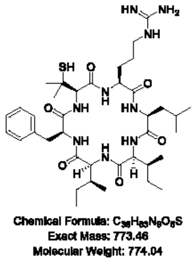

cyclo-hCRLIIF

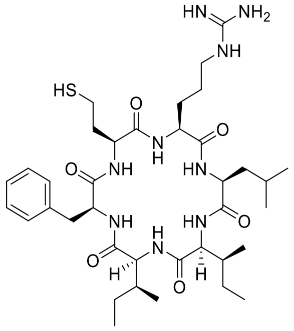

The linear peptide was synthesized and purified as above, and (47 mg, 0.06 mmol) was dissolved in MeOH (1 mL). To the stirring mixture was added dropwise a solution of aldrithiol-2 (26 mg, 0.12 mmol, 2 eq) in MeOH (1 mL). The reaction was left to stir at room temperature for 30 min after which no starting material was detected by LCMS. The reaction mixture was concentrated to dryness on a rotary evaporator, and to the residue was added Et_2O (2 mL). The yellow ether solution was removed, and the precipitate washed a further two times with Et_2O. The resulting solid was dried and isolated as an off-white solid (36 mg, 68%) and used without further purification. LCMS R_t: 1.96 min, LRMS *m/z* (ESI^+): 444.7 [M+2H] (100%).

The above product (36 mg, 0.041 mmol), HATU (19 mg), HOAt (11 mg) and DIPEA (21 µL) were mixed in DMF (40 mL) following the general procedure above. The product was purified by RP-HPLC and lyophilised to obtain a white solid (12 mg, 34%). LCMS R_t: 2.06 min, LRMS *m/z* (ESI^+) 435.6 [M+2H] (100%), 869.7 [M+H] (20%).

The above product (12 mg, 0.025 mmol), DTT (77 mg) and 1M aq. $(NH_4)_2CO_3$ (0.5 mL) were mixed in DMF (0.5 mL) following the general procedure above. The product was purified by RP-HPLC and lyophilised to obtain the product as a white solid (6 mg, 8%). LCMS R_t: 1.95 min, Analytical HPLC R_t: 12.750 min (84% purity) LRMS *m/z* (ESI^+): 381.1 [M+2H] (100%), 760.7 [M+H] (90%), HRMS *m/z* (ESI^+): calc. for $C_{37}H_{62}N_9O_6S^+$ ([M+H^+]) 760.4538, found 760.4533.

a)

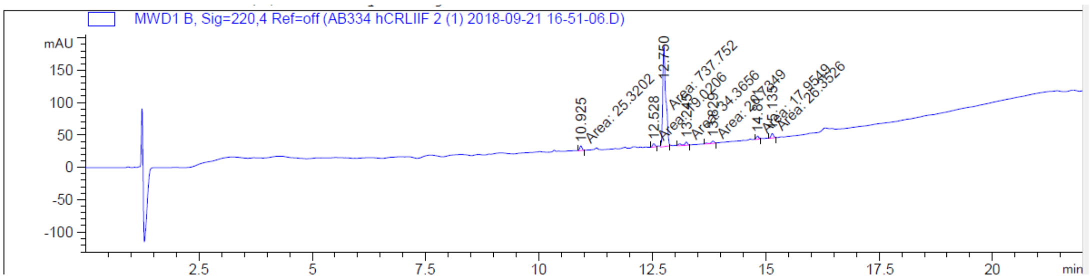

b)

Peak #	RetTime [min]	Type	Width [min]	Area [mAU*s]	Height [mAU]	Area %
1	10.925	MM	0.0584	25.32016	7.22924	2.8724
2	12.528	MM	0.0616	19.02062	5.14358	2.1578
3	12.750	MM	0.0779	737.75195	157.93483	83.6927
4	13.245	MM	0.1112	34.36560	5.15100	3.8985
5	13.829	MM	0.0980	20.73487	3.52498	2.3522
6	14.811	MM	0.0630	17.95490	4.75324	2.0369
7	15.135	MM	0.0626	26.35263	7.02035	2.9895

c)

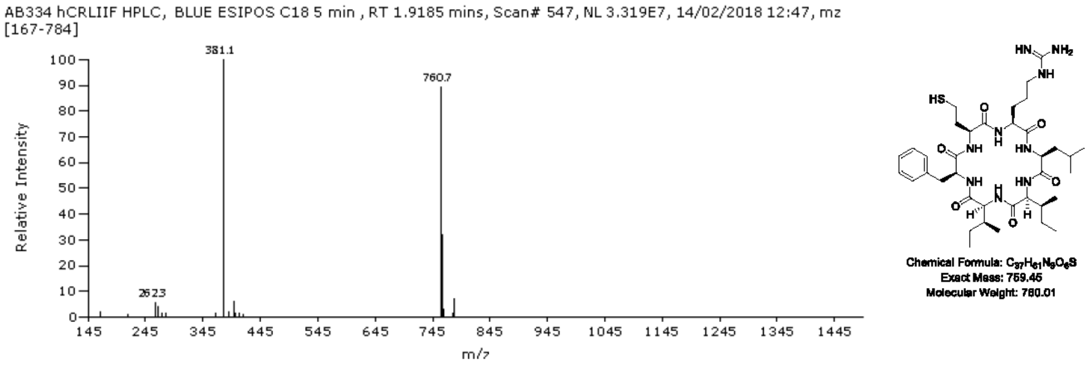

cyclo-MRLIIF

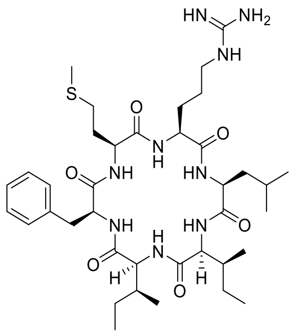

The linear peptide FMRLII was synthesised from Fmoc-Ile-Wang resin (Novabiochem) on a 0.1 mmol scale following the general procedure above. After precipitation from ether, the peptide was dissolved in DMF and cyclised following the general procedure above. The product was purified by RP-HPLC and lyophilised to give a white solid (47 mg, 61% overall yield). LCMS R_t: 2.31 min, Analytical HPLC R_t: 12.926 min (79% purity), LRMS *m/z* (ESI$^+$): 388.1 [M+2H] (100%), 774.9 [M+2H] (40%), HRMS *m/z* (ESI$^+$): calc. for $C_{38}H_{64}N_9O_6S^+$ ([M+H$^+$]) 774.4695, found 774.4687.

a)

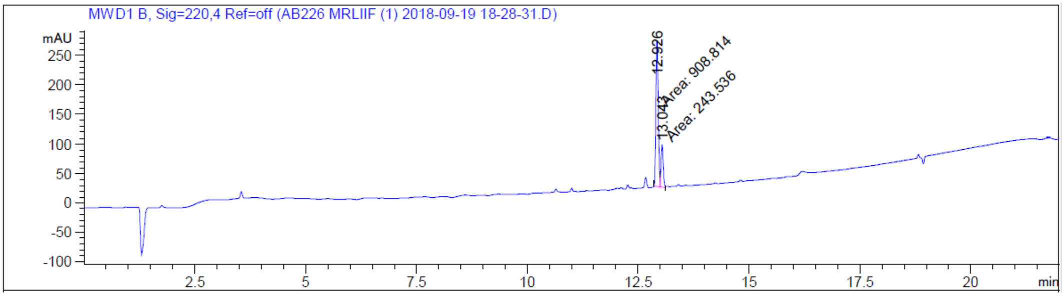

b)

Peak #	RetTime [min]	Type	Width [min]	Area [mAU*s]	Height [mAU]	Area %
1	12.926	MM	0.0608	908.81366	249.07721	78.8661
2	13.043	MM	0.0561	243.53629	72.38391	21.1339

c)

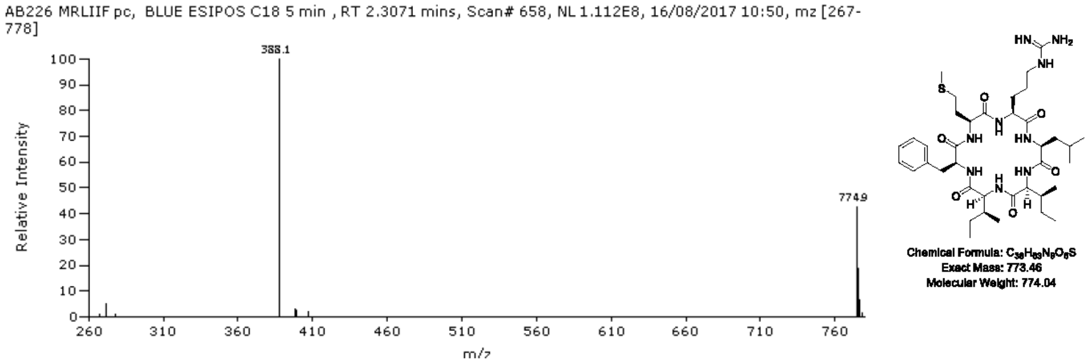

cyclo-SRLIIF

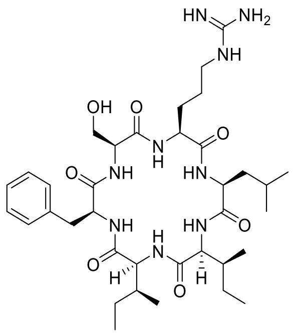

The linear peptide FSRLII was synthesised from Fmoc-Ile-Wang resin (Novabiochem) on a 0.1 mmol scale following the general procedure above. After precipitation from ether, the peptide was dissolved in DMF and cyclised following the general procedure above. The product was purified by RP-HPLC and lyophilised to give a white solid (16 mg, 22% overall yield). LCMS R_t: 2.13 min, Analytical HPLC R_t: 11.500 min (95% purity), LRMS *m/z* (ESI$^+$): 366.1 [M+2H] (100%), 730.8 [M+H] (40%), HRMS *m/z* (ESI$^+$): calc. for $C_{36}H_{60}N_9O_7^+$ ([M+H$^+$]) 730.4610, found 730.4616.

a)

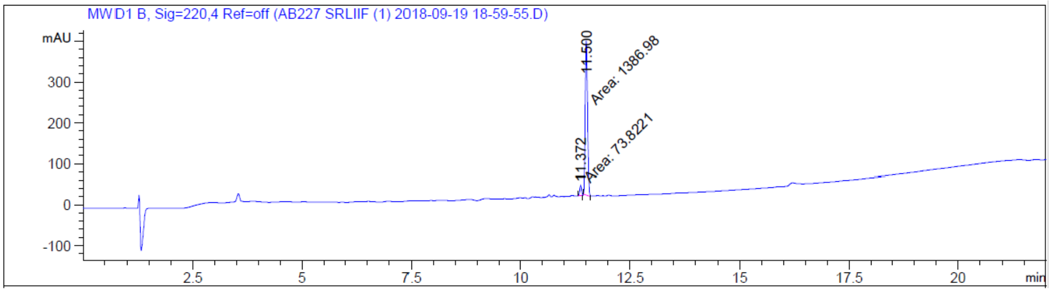

b)

Peak #	RetTime [min]	Type	Width [min]	Area [mAU*s]	Height [mAU]	Area %
1	11.372	MM	0.0496	73.82207	24.81717	5.0535
2	11.500	MM	0.0611	1386.98376	378.36252	94.9465

c)

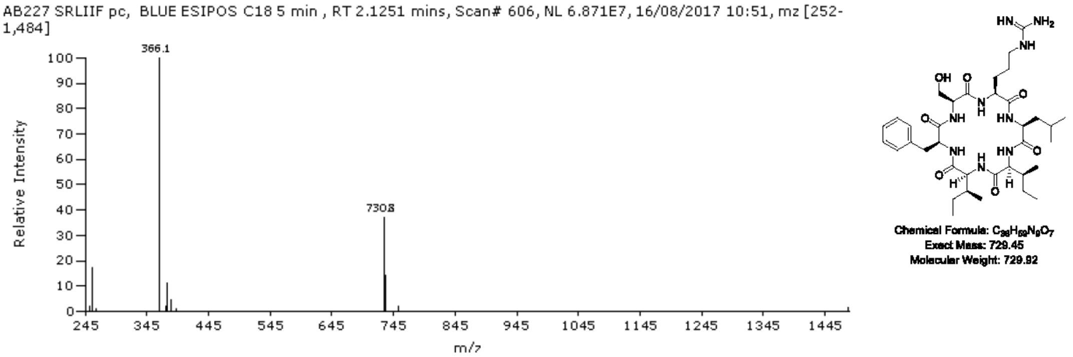

<u>*cyclo*-TRLIIF</u>

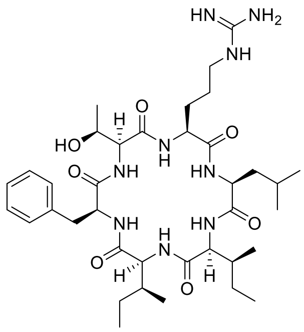

The linear peptide FTRLII was synthesised from Fmoc-Ile-Wang resin (Novabiochem) on a 0.1 mmol scale following the general procedure above. After precipitation from ether, the peptide was dissolved in DMF and cyclised following the general procedure above. The product was purified by RP-HPLC and lyophilised to give a white solid (26 mg, 35% overall yield). LCMS R_t: 2.17 min, Analytical HPLC R_t: 11.838 min (94% purity), LRMS *m/z* (ESI$^+$): 373.1 [M+2H] (100%), 744.8 [M+H] (50%), HRMS *m/z* (ESI$^+$): calc. for $C_{37}H_{62}N_9O_7^+$ ([M+H$^+$]) 744.4767, found 744.4763.

a)

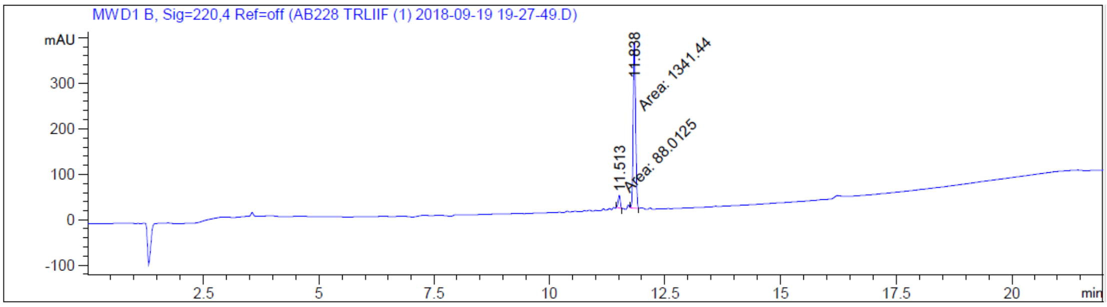

b)

Peak #	RetTime [min]	Type	Width [min]	Area [mAU*s]	Height [mAU]	Area %
1	11.513	MM	0.0524	88.01251	27.99261	6.1571
2	11.838	MM	0.0608	1341.44214	367.50507	93.8429

c)

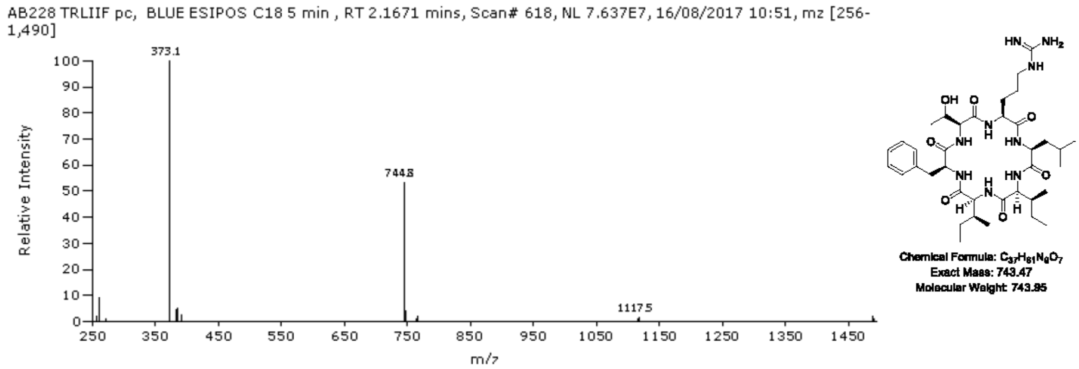

cyclo-C(Me)RLIIF

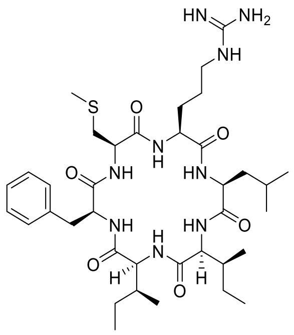

To a stirring solution of CRLIIF (8.0 mg, 0.011 mmol, 1 eq.) and MeI (1 µL, 2.3 mg, 0.016 mmol, 1.5 eq.) in 1 mL MeCN, was added DIPEA (2.8 µL, 2.1 mg, 0.016 mmol, 1.5 eq.). The mixture was stirred at room temperature for 3 hours, after which the product was purified by RP-HPLC and lyophilised to give a white solid (7 mg, 76% yield). LCMS R_t: 1.91 min, Analytical HPLC R_t: 12.722 min (89% purity), LRMS *m/z* (ESI$^+$): 760.6 [M+H] (100%), 381.0 [M+2H] (75%), HRMS *m/z* (ESI$^+$): calc. for $C_{37}H_{62}N_9O_6S^+$ ([M+H$^+$]) 760.4538, found 760.4542.

a)

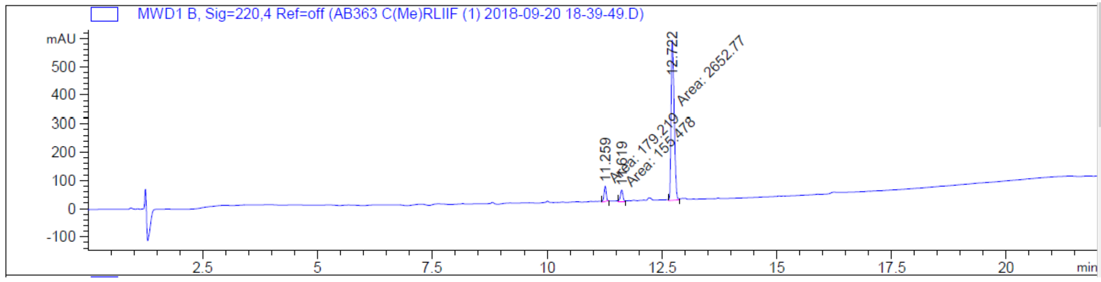

b)

Peak #	RetTime [min]	Type	Width [min]	Area [mAU*s]	Height [mAU]	Area %
1	11.259	MM	0.0552	179.21947	54.10040	5.9990
2	11.619	MM	0.0632	155.47798	41.02829	5.2043
3	12.722	MM	0.0778	2652.77295	567.99023	88.7966

c)

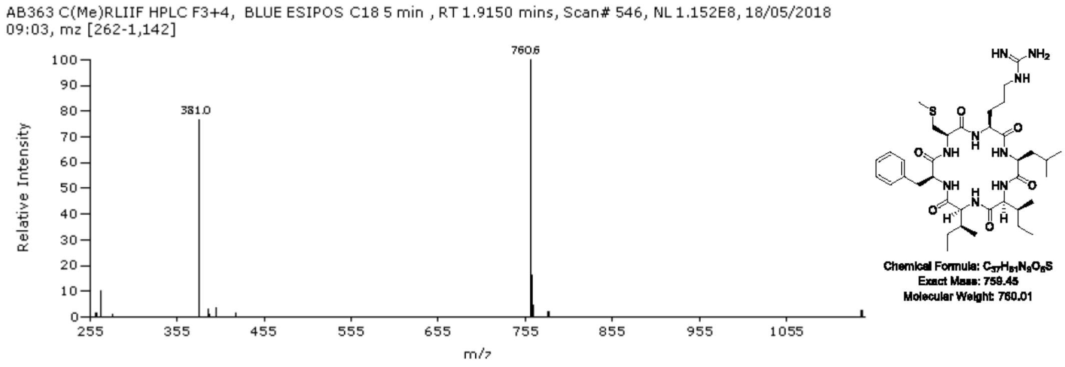

cyclo-CRLIIF(4-I)

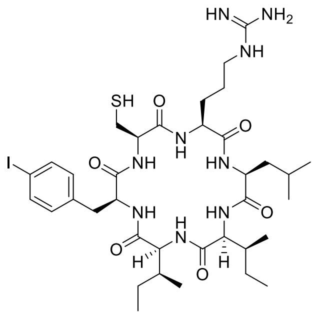

The linear peptide F(4-I)C(StBu)RLII was synthesised from Fmoc-Ile-Wang resin (Novabiochem) on a 0.1 mmol scale following the general procedure above. After precipitation from ether, the peptide was dissolved in DMF and cyclised following the general procedure above. The peptide was concentrated, and then deprotected following the general procedure above. The product was purified by RP-HPLC and lyophilised to give a white solid (46 mg, 53% overall yield). LCMS R_t: 2.49 min, Analytical HPLC R_t: 13.865 min (99% purity), LRMS *m/z* (ESI$^+$): 437.1 [M+2H] (100%), 872.7 [M+H] (60%), HRMS *m/z* (ESI$^+$): calc. for $C_{36}H_{59}IN_9O_6S^+$ ([M+H$^+$]) 872.3348, found 872.3365.

a)

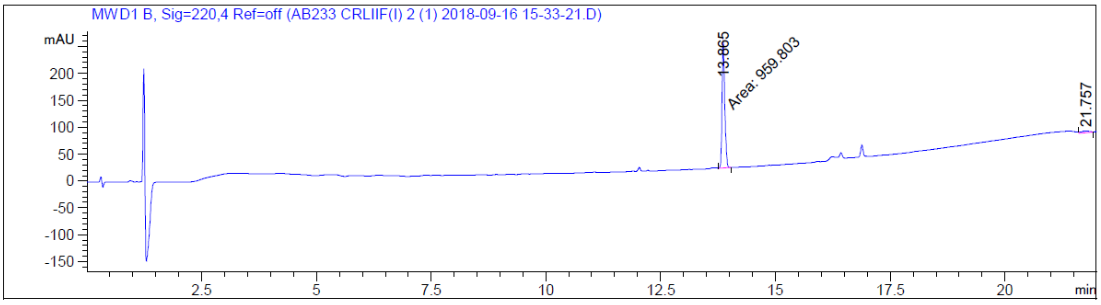

b)

Peak #	RetTime [min]	Type	Width [min]	Area [mAU*s]	Height [mAU]	Area %
1	13.865	FM	0.0676	959.80334	236.60320	100.0000

c)

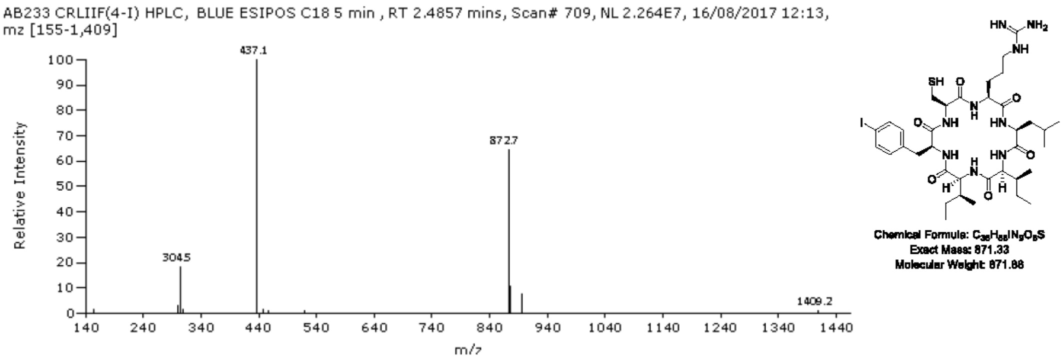

cyclo-CRLIIF(4-Br)

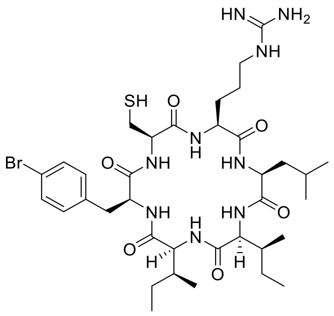

The linear peptide F(4-Br)C(StBu)RLII was synthesised from Fmoc-Ile-Wang resin (Novabiochem) on a 0.1 mmol scale following the general procedure above. After precipitation from ether, the peptide was dissolved in DMF and cyclised following the general procedure above. The peptide was concentrated, and then deprotected following the general procedure above. The product was purified by RP-HPLC and lyophilised to give a white solid (48 mg, 59% overall yield). LCMS R_t: 2.01 min, Analytical HPLC R_t: 13.592 min (94% purity), LRMS *m/z* (ESI^+): 826.7 [M+H, ^{81}Br] (100%), 824.8 [M+H, ^{79}Br] (95%), HRMS *m/z* (ESI^+): calc. for $C_{36}H_{59}BrN_9O_6S^+$ ($[M+H^+]$) 824.3487/826.3466, found 824.3487/826.3477.

a)

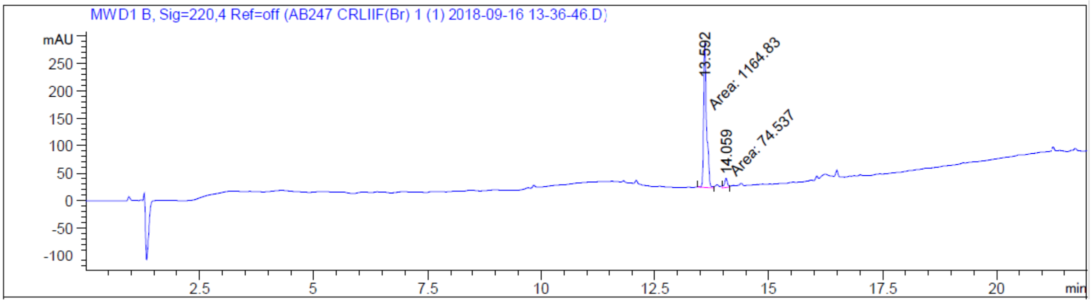

b)

Peak #	RetTime [min]	Type	Width [min]	Area [mAU*s]	Height [mAU]	Area %
1	13.592	MM	0.0726	1164.82617	267.38196	93.9859
2	14.059	MM	0.0746	74.53699	16.64364	6.0141

c)

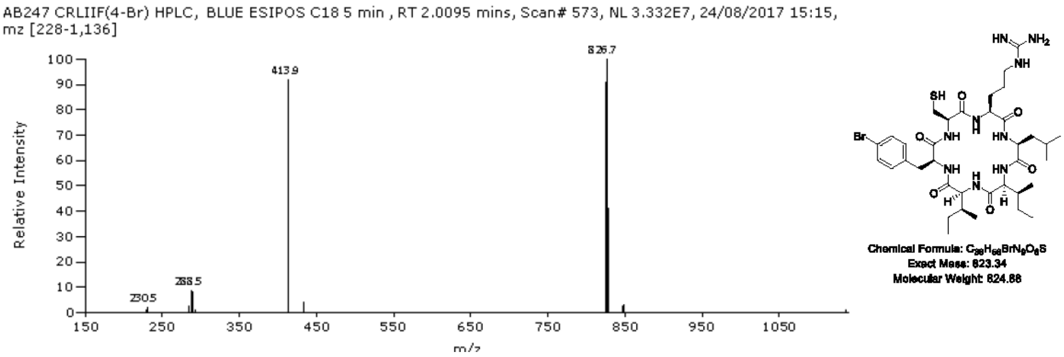

cyclo-CRLIIF(4-CF$_3$)

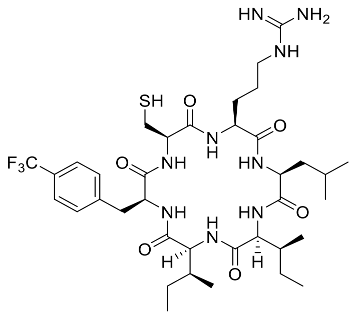

The linear peptide F(4-CF$_3$)C(StBu)RLII was synthesised from Fmoc-Ile-Wang resin (Novabiochem) on a 0.05 mmol scale following the general procedure above. After precipitation from ether, the peptide was dissolved in DMF and cyclised following the general procedure above. The peptide was concentrated, and then deprotected following the general procedure above. The product was purified by RP-HPLC and lyophilised to give a white solid (12 mg, 30% overall yield). LCMS R_t: 2.01 min, Analytical HPLC R_t: 13.742 min (99% purity), LRMS *m/z* (ESI$^+$): 408.1 [M+2H] (100%), 814.8 [M+H] (80%), HRMS *m/z* (ESI$^+$): calc. for $C_{37}H_{59}F_3N_9O_6S^+$ ([M+H$^+$]) 814.4256, found 814.4256.

a)

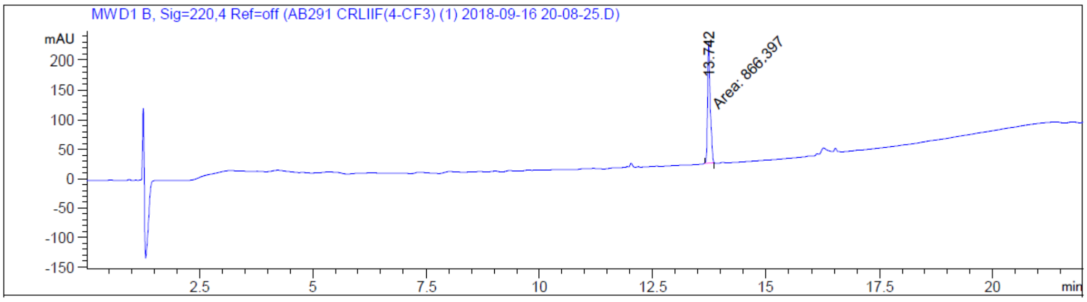

b)

Peak #	RetTime [min]	Type	Width [min]	Area [mAU*s]	Height [mAU]	Area %
1	13.742	MM	0.0700	866.39673	206.16460	100.0000

c)

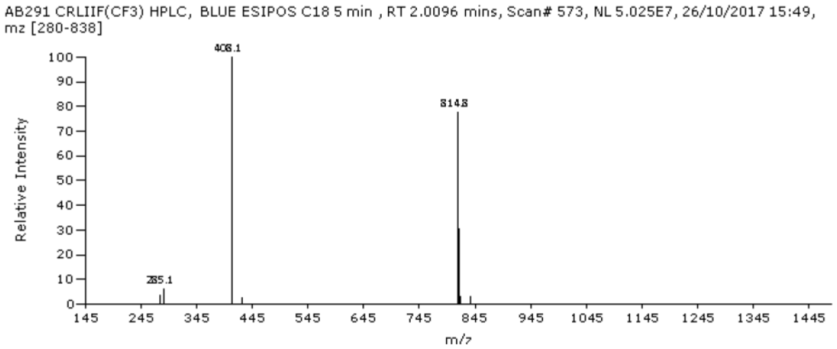

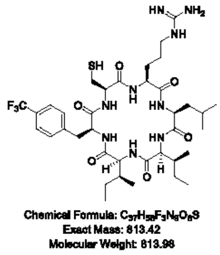

cyclo-CRLIIF(4-Cl)

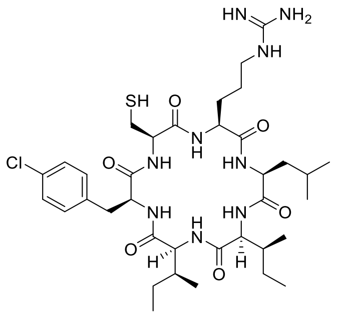

The linear peptide F(4-Cl)C(StBu)RLII was synthesised from Fmoc-Ile-Wang resin (Novabiochem) on a 0.1 mmol scale following the general procedure above. After precipitation from ether, the peptide was dissolved in DMF and cyclised following the general procedure above. The peptide was concentrated, and then deprotected following the general procedure above. The product was purified by RP-HPLC and lyophilised to give a white solid (52 mg, 67% overall yield). LCMS R_t: 1.99 min, Analytical HPLC R_t: 13.327 min (82% purity), LRMS *m/z* (ESI$^+$): 391.2 [M+2H] (100%), 780.8 [M+H] (75%), HRMS *m/z* (ESI$^+$): calc. for $C_{36}H_{59}ClN_9O_6S^+$ ([M+H$^+$]) 780.3992, found 780.3978.

a)

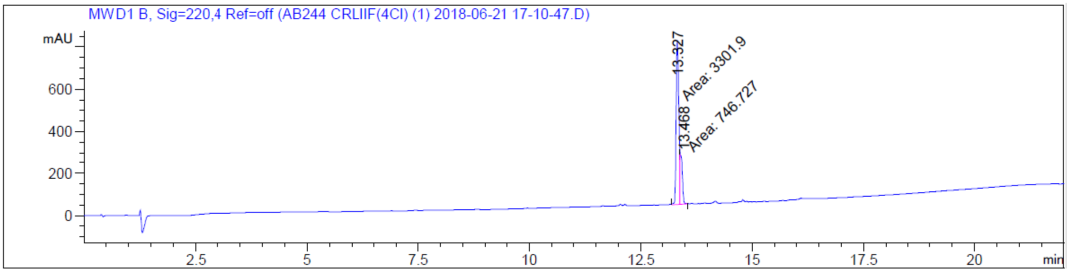

b)

Peak #	RetTime [min]	Type	Width [min]	Area [mAU*s]	Height [mAU]	Area %
1	13.327	FM	0.0685	3301.89600	803.42743	81.5560
2	13.468	MF	0.0518	746.72729	240.22414	18.4440

c)

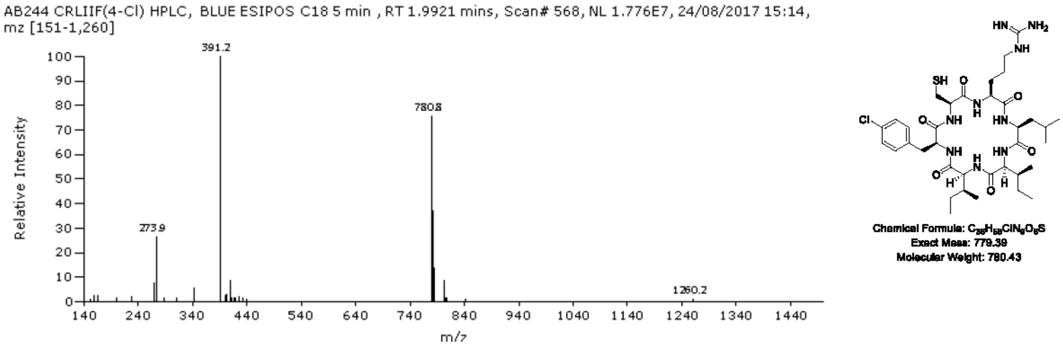

cyclo-CRLII(hF)

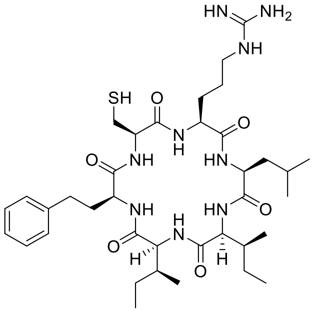

The linear peptide (hF)C(StBu)RLII was synthesised from Fmoc-Ile-Wang resin (Novabiochem) on a 0.05 mmol scale following the general procedure above. After precipitation from ether, the peptide was dissolved in DMF and cyclised following the general procedure above. The peptide was concentrated, and then deprotected following the general procedure above. The product was purified by RP-HPLC and lyophilised to give a white solid (14 mg, 36% overall yield). LCMS R_t: 1.93 min, Analytical HPLC R_t: 13.089 min (85% purity), LRMS *m/z* (ESI$^+$): 381.1 [M+2H] (100%), 760.8 [M+H] (45%), HRMS *m/z* (ESI$^+$): calc. for $C_{37}H_{62}N_9O_6S^+$ ([M+H$^+$]) 760.4538, found 760.4538.

a)

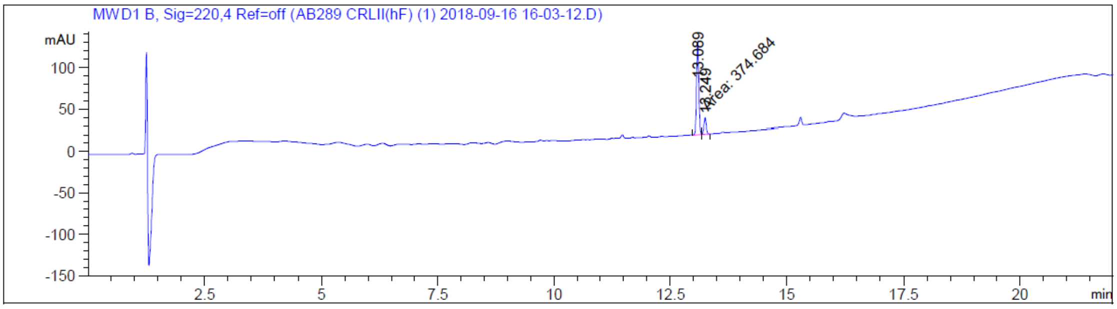

b)

Peak #	RetTime [min]	Type	Width [min]	Area [mAU*s]	Height [mAU]	Area %
1	13.089	FM	0.0560	374.68381	111.45501	85.3319
2	13.249	VB	0.0563	64.40622	18.65333	14.6681

c)

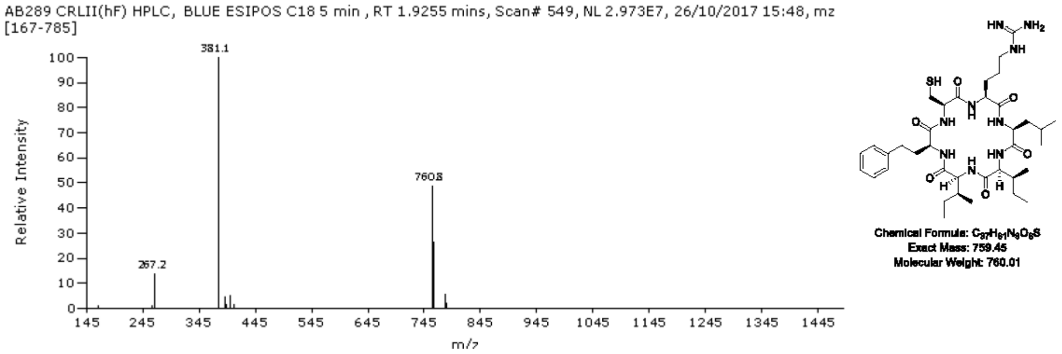

<u>*cyclo*-CRLII(1-Nal)</u>

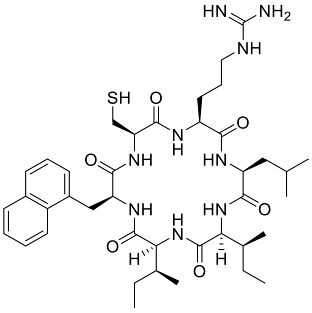

The linear peptide (1-Nal)C(StBu)RLII was synthesised from Fmoc-Ile-Wang resin (Novabiochem) on a 0.1 mmol scale following the general procedure above. After precipitation from ether, the peptide was dissolved in DMF and cyclised following the general procedure above. The peptide was concentrated, and then deprotected following the general procedure above. The product was purified by RP-HPLC and lyophilised to give a white solid (47 mg, 59% overall yield). LCMS R_t: 2.05 min, Analytical HPLC R_t: 14.025 min (87% purity), LRMS *m/z* (ESI$^+$): 399.2 [M+2H] (100%), 796.9 [M+H] (60%), HRMS *m/z* (ESI$^+$): calc. for $C_{40}H_{62}N_9O_6S^+$ ([M+H$^+$]) 796.4538, found 796.4556.

a)

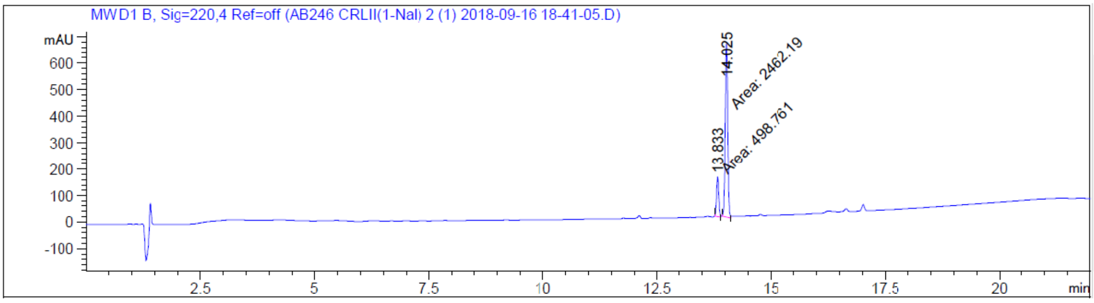

b)

Peak #	RetTime [min]	Type	Width [min]	Area [mAU*s]	Height [mAU]	Area %
1	13.833	MM	0.0552	498.76111	150.65335	16.8446
2	14.025	MM	0.0618	2462.18604	664.34113	83.1554

c)

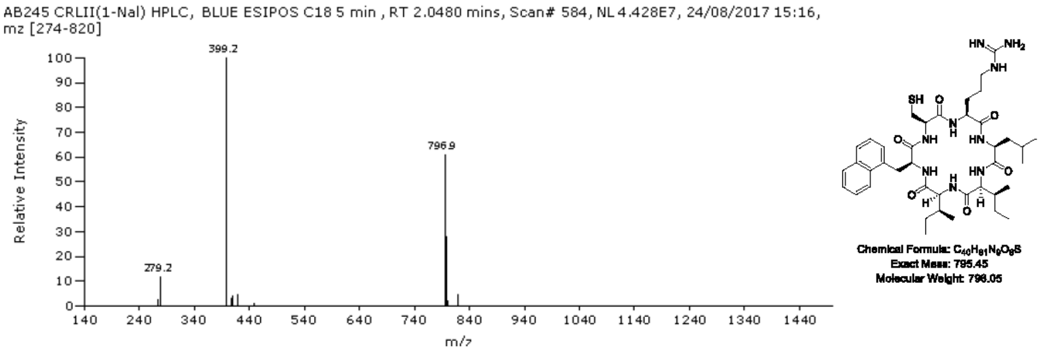

cyclo-CRLIIF(4-F)

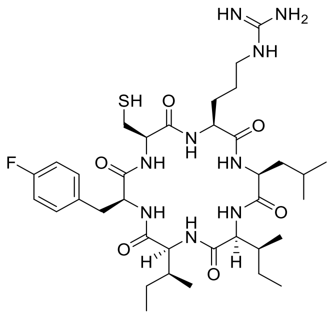

The linear peptide F(4-F)C(StBu)RLII was synthesised from Fmoc-Ile-Wang resin (Novabiochem) on a 0.1 mmol scale following the general procedure above. After precipitation from ether, the peptide was dissolved in DMF and cyclised following the general procedure above. The peptide was concentrated, and then deprotected following the general procedure above. The product was purified by RP-HPLC and lyophilised to give a white solid (43 mg, 57% overall yield). LCMS R_t: 2.32 min, Analytical HPLC R_t: 12.686 min (87% purity), LRMS *m/z* (ESI$^+$): 383.1 [M+2H] (100%), 764.8 [M+H] (47%), HRMS *m/z* (ESI$^+$): calc. for $C_{36}H_{59}FN_9O_6S^+$ ([M+H$^+$]) 764.4288, found 764.4286.

a)

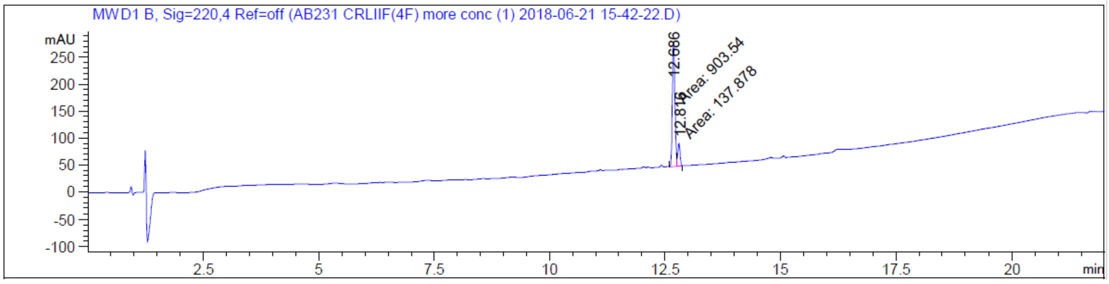

b)

Peak #	RetTime [min]	Type	Width [min]	Area [mAU*s]	Height [mAU]	Area %
1	12.686	FM	0.0625	903.54047	240.85384	86.7606
2	12.816	MF	0.0496	137.87791	46.34242	13.2394

c)

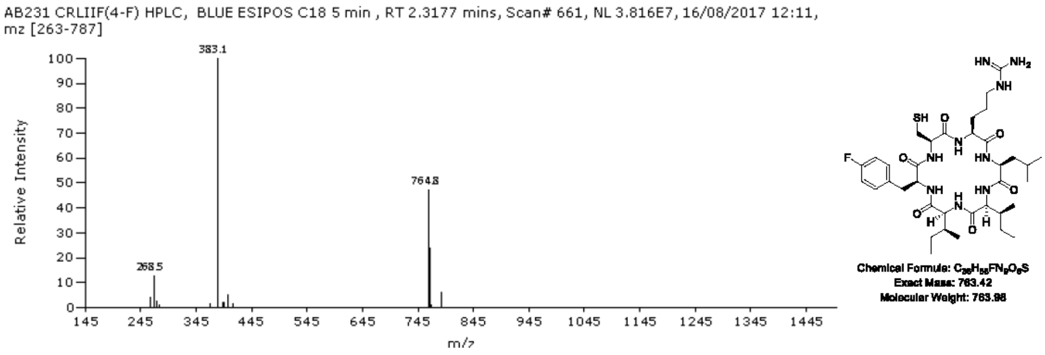

<u>*cyclo*-CRLIIF(4-Bz)</u>

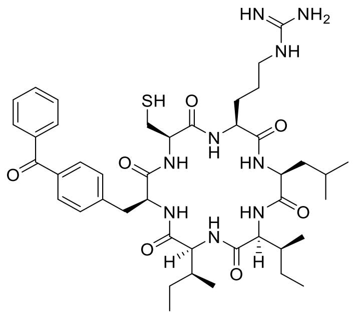

The linear peptide F(4-Bz)C(StBu)RLII was synthesised from Fmoc-Ile-Wang resin (Novabiochem) on a 0.1 mmol scale following the general procedure above. After precipitation from ether, the peptide was dissolved in DMF and cyclised following the general procedure above. The peptide was concentrated, and then deprotected following the general procedure above. The product was purified by RP-HPLC and lyophilised to give a white solid (47 mg, 55% overall yield). LCMS R_t: 2.01 min, Analytical HPLC R_t: 13.511 min (99% purity), LRMS *m/z* (ESI$^+$): 850.9 [M+H] (100%), 426.1 [M+2H] (90%), HRMS *m/z* (ESI$^+$): calc. for $C_{43}H_{64}N_9O_7S^+$ ([M+H$^+$]) 850.4644, found 850.4659.

a)

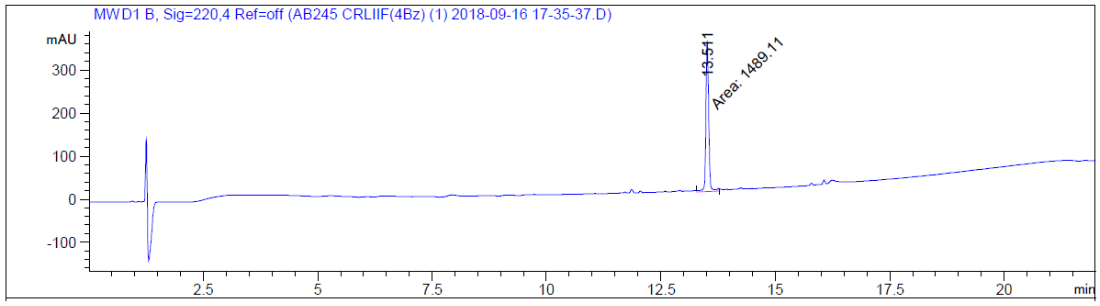

b)

Peak #	RetTime [min]	Type	Width [min]	Area [mAU*s]	Height [mAU]	Area %
1	13.511	MM	0.0712	1489.10889	348.75256	100.0000

c)

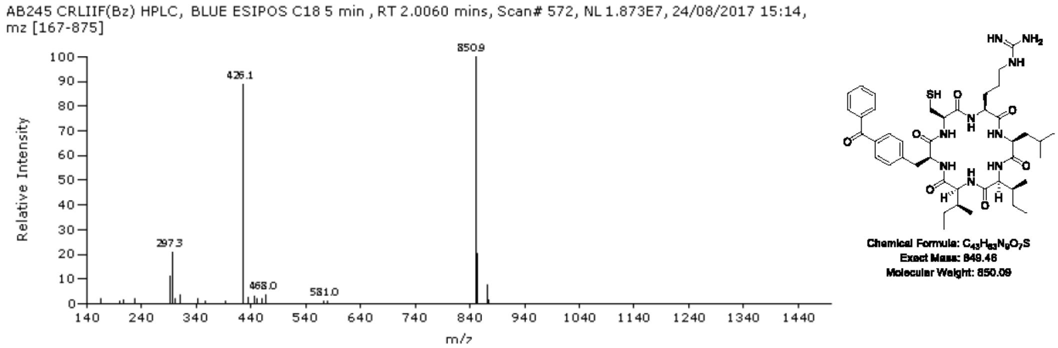

cyclo-CRLIIF(4-NO_2)

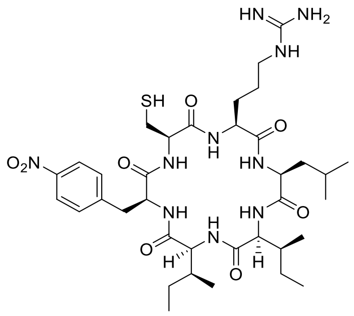

The linear peptide F(4-NO_2)C(StBu)RLII was synthesised from Fmoc-Ile-Wang resin (Novabiochem) on a 0.2 mmol scale following the general procedure above. After precipitation from ether, the peptide was dissolved in DMF and cyclised following the general procedure above. The peptide was concentrated, and then deprotected following the general procedure above. The product was purified by RP-HPLC and lyophilised to give a white solid (90 mg, 57% overall yield). LCMS R_t: 1.86 min, Analytical HPLC R_t: 12.507 min (99% purity), LRMS *m/z* (ESI^+): 791.9 [M+H] (100%), 396.6 [M+2H], HRMS *m/z* (ESI^+): calc. for $C_{36}H_{59}N_{10}O_8S^+$ ([M+H^+]) 791.4233, found 791.4230.

a)

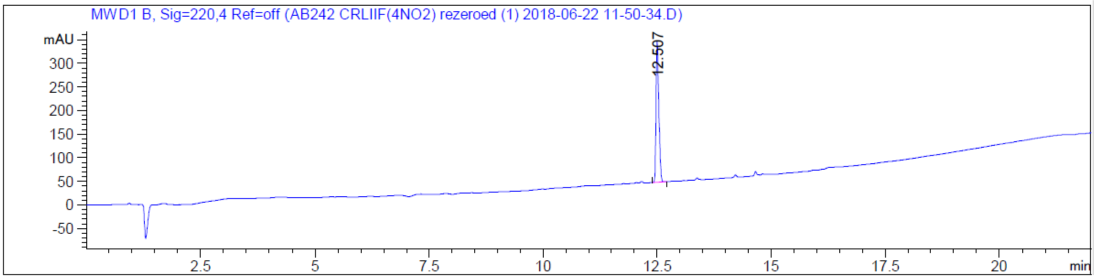

b)

Peak #	RetTime [min]	Type	Width [min]	Area [mAU*s]	Height [mAU]	Area %
1	12.507	BB	0.0840	1271.49756	285.42160	100.0000

c)

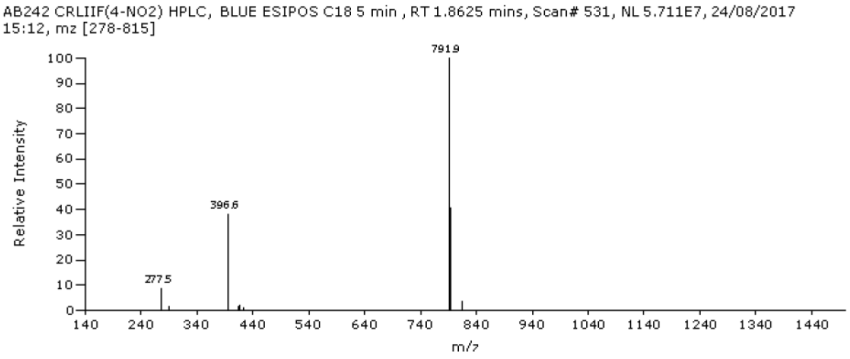

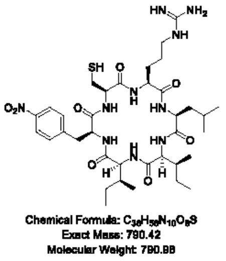

cyclo-CRLIIF(4-CN)

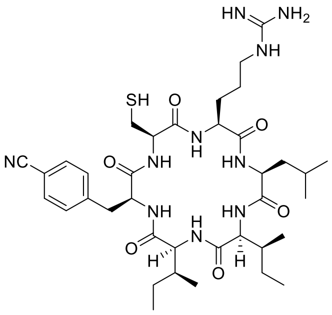

The linear peptide F(4-CN)C(StBu)RLII was synthesised from Fmoc-Ile-Wang resin (Novabiochem) on a 0.1 mmol scale following the general procedure above. After precipitation from ether, the peptide was dissolved in DMF and cyclised following the general procedure above. The peptide was concentrated, and then deprotected following the general procedure above. The product was purified by RP-HPLC and lyophilised to give a white solid (38 mg, 49% overall yield). LCMS R_t: 2.19 min, Analytical HPLC R_t: 12.002 min (86% purity), LRMS *m/z* (ESI^+): 771.8 [M+H] (100%), 386.6 [M+2H] (75%), HRMS *m/z* (ESI^+): calc. for $C_{37}H_{59}N_{10}O_6S^+$ ([M+H$^+$]) 771.4334, found 771.4319.

a)

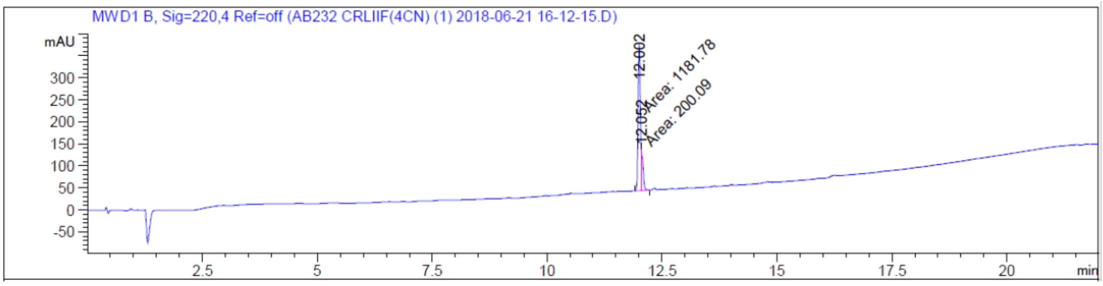

b)

Peak #	RetTime [min]	Type	Width [min]	Area [mAU*s]	Height [mAU]	Area %
1	12.002	FM	0.0625	1181.78186	315.01587	85.5204
2	12.052	FM	0.0352	200.08960	94.71465	14.4796

c)

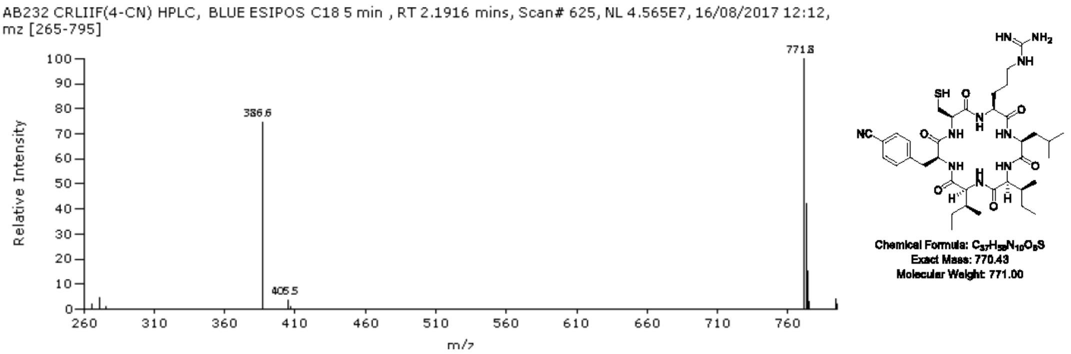

<u>*cyclo*-CRLII(Phg)</u>

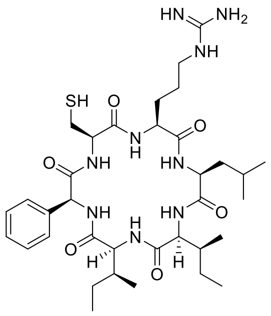

The linear peptide (Phg)C(StBu)RLII was synthesised from Fmoc-Ile-Wang resin (Novabiochem) on a 0.05 mmol scale following the general procedure above. After precipitation from ether, the peptide was dissolved in DMF and cyclised following the general procedure above. The peptide was concentrated, and then deprotected following the general procedure above. The product was purified by RP-HPLC and lyophilised to give a white solid (15 mg, 41% overall yield). LCMS R_t: 1.79 min, Analytical HPLC R_t: 12.113 min (93% purity), LRMS *m/z* (ESI^+): 367.1 [M+2H] (100%), 732.8 [M+H] (40%), HRMS *m/z* (ESI^+): calc. for $C_{35}H_{58}N_9O_6S^+$ ($[M+H^+]$) 732.4225, found 732.4230.

a)

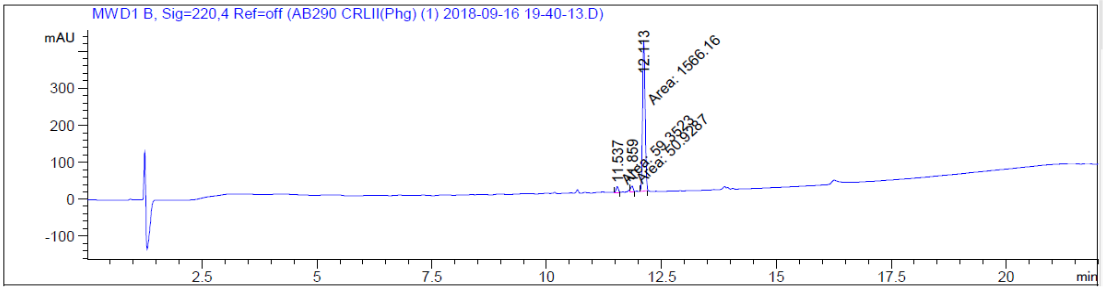

b)

Peak #	RetTime [min]	Type	Width [min]	Area [mAU*s]	Height [mAU]	Area %
1	11.537	MM	0.0590	59.35228	16.76459	3.5404
2	11.859	MM	0.0598	50.92872	14.18797	3.0379
3	12.113	MM	0.0640	1566.16394	407.92014	93.4217

c)

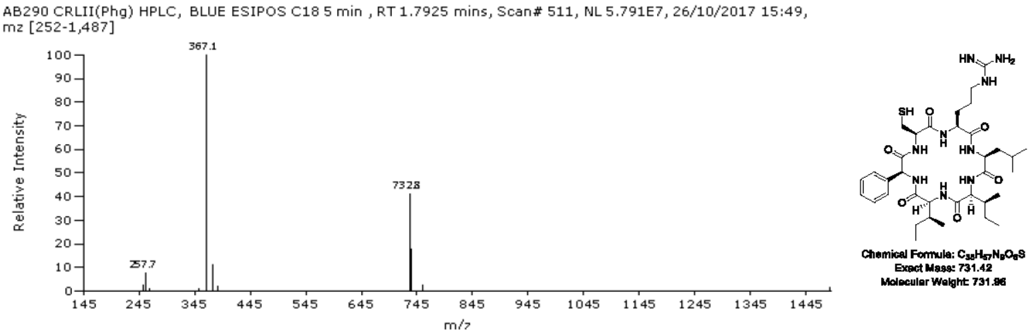

cyclo-CRLIIY

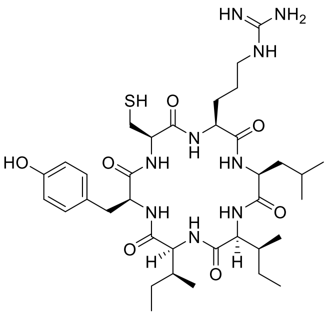

The linear peptide YC(StBu)RLII was synthesised from Fmoc-Ile-Wang resin (Novabiochem) on a 0.1 mmol scale following the general procedure above. After precipitation from ether, the peptide was dissolved in DMF and cyclised following the general procedure above. The peptide was concentrated, and then deprotected following the general procedure above. The product was purified by RP-HPLC and lyophilised to give a white solid (50 mg, 66% overall yield). LCMS R_t: 1.64 min, Analytical HPLC R_t: 10.951 min (85% purity), LRMS *m/z* (ESI^+): 382.1 [M+2H] (100%), 762.8 [M+H] (75%), HRMS *m/z* (ESI^+): calc. for $C_{36}H_{60}N_9O_7S^+$ ($[M+H]^+$) 762.4331, found 762.4316.

a)

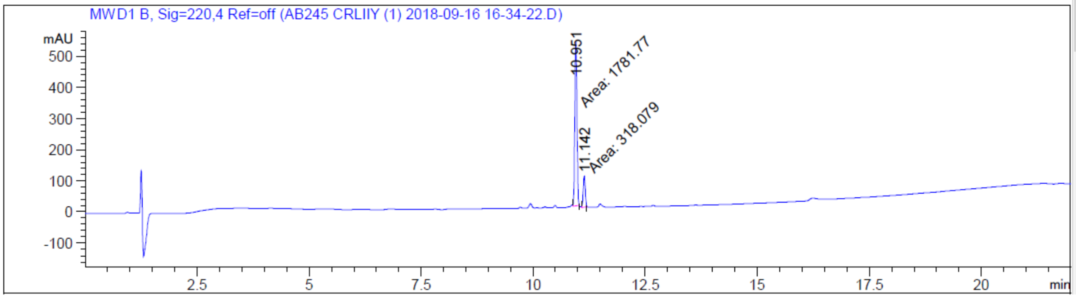

b)

Peak #	RetTime [min]	Type	Width [min]	Area [mAU*s]	Height [mAU]	Area %
1	10.951	MM	0.0561	1781.77185	529.22388	84.8523
2	11.142	MM	0.0524	318.07880	101.17374	15.1477

c)

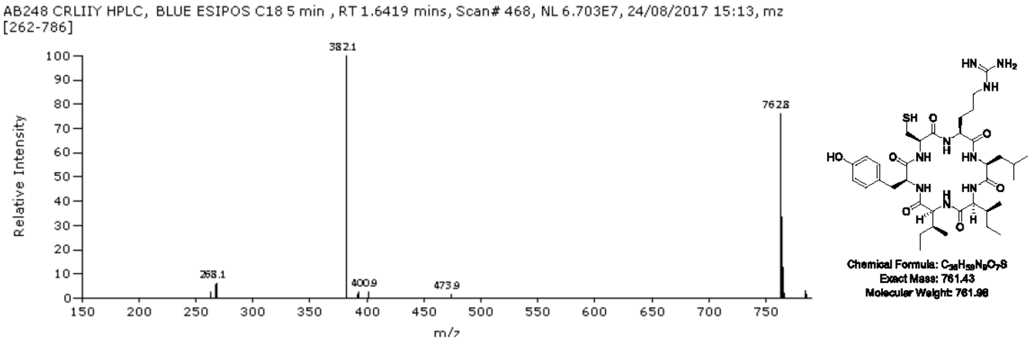

<u>*cyclo*-CRLIIY(Me)</u>

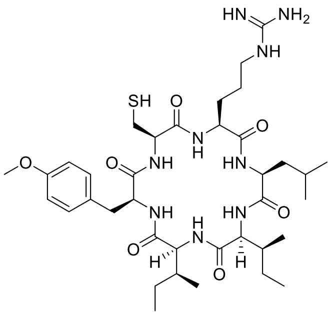

The linear peptide Y(Me)C(StBu)RLII was synthesised from Fmoc-Ile-Wang resin (Novabiochem) on a 0.1 mmol scale following the general procedure above. After precipitation from ether, the peptide was dissolved in DMF and cyclised following the general procedure above. The peptide was concentrated, and then deprotected following the general procedure above. The product was purified by RP-HPLC and lyophilised to give a white solid (42 mg, 54% overall yield). LCMS R_t: 2.25 min, Analytical HPLC R_t: 12.411 min (88% purity), LRMS *m/z* (ESI^+): 389.1 [M+2H] (100%), 776.8 [M+H] (50%). HRMS *m/z* (ESI^+): calc. for $C_{37}H_{62}IN_9O_7S^+$ ($[M+H^+]$) 776.4487, found 776.4475.

a)

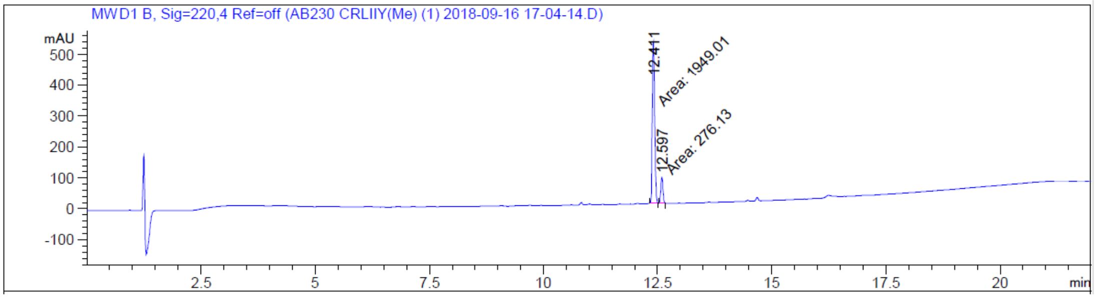

b)

Peak #	RetTime [min]	Type	Width [min]	Area [mAU*s]	Height [mAU]	Area %
1	12.411	MM	0.0614	1949.01367	529.06903	87.5905
2	12.597	MM	0.0537	276.12973	85.74096	12.4095

c)

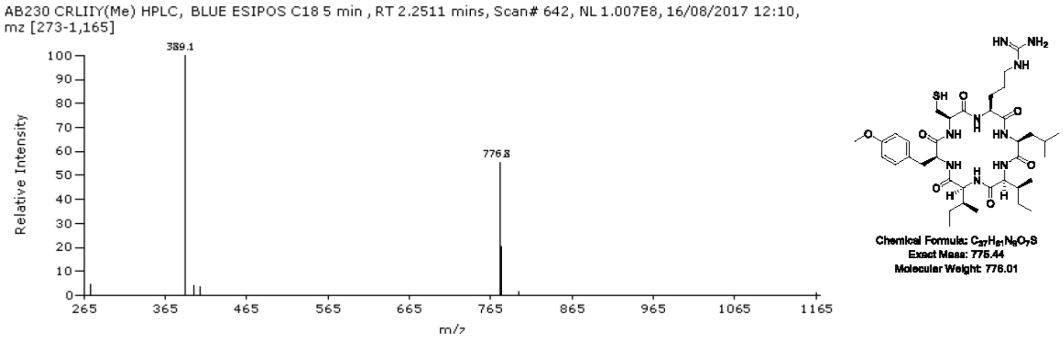

<u>*cyclo*-CRLII(4-Pal)</u>

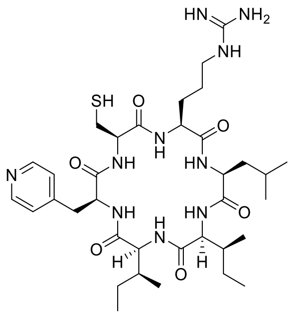

The linear peptide (4-Pal)C(StBu)RLII was synthesised from Fmoc-Ile-Wang resin (Novabiochem) on a 0.1 mmol scale following the general procedure above. After precipitation from ether, the peptide was dissolved in DMF and cyclised following the general procedure above. The peptide was concentrated, and then deprotected following the general procedure above. The product was purified by RP-HPLC and lyophilised to give a white solid (38 mg, 51% overall yield). LCMS R_t: 1.27 min, Analytical HPLC R_t: 8.884 min (85% purity), LRMS *m/z* (ESI$^+$): 374.6 [M+2H] (100%), 747.8 [M+H] (5%), HRMS *m/z* (ESI$^+$): calc. for $C_{35}H_{59}N_{10}O_6S^+$ ([M+H$^+$]) 747.4334, found 747.4319.

a)

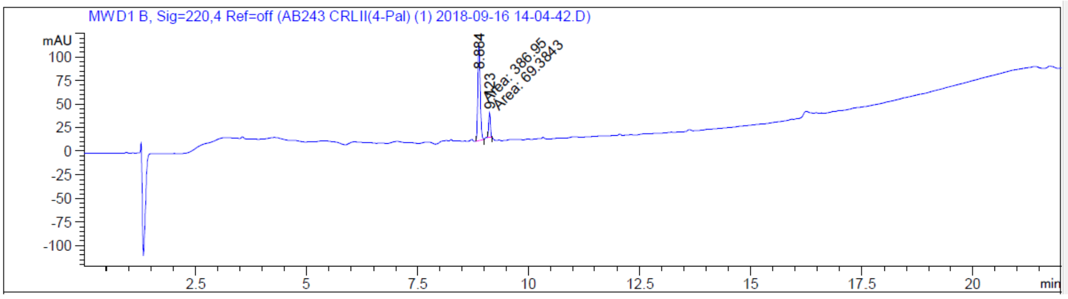

b)

Peak #	RetTime [min]	Type	Width [min]	Area [mAU*s]	Height [mAU]	Area %
1	8.884	MM	0.0619	386.94995	104.11131	84.7953
2	9.123	MM	0.0440	69.38430	26.29163	15.2047

c)

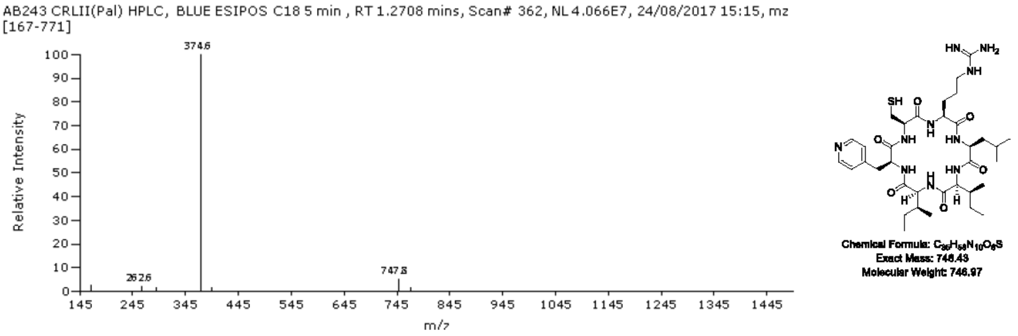

<u>*cyclo*-CRLII(D-Phe)</u>

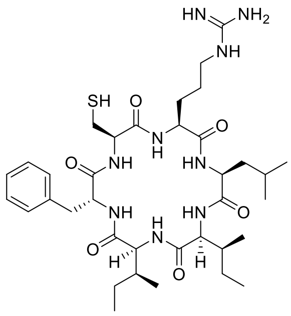

The linear peptide (D-Phe)C(StBu)RLII was synthesised from Fmoc-Ile-Wang resin (Novabiochem) on a 0.1 mmol scale following the general procedure above. After precipitation from ether, the peptide was dissolved in DMF and cyclised following the general procedure above. The peptide was concentrated, and then deprotected following the general procedure above. The product was purified by RP-HPLC and lyophilised to give a white solid (17 mg, 23% overall yield). LCMS R_t: 1.79 min, Analytical HPLC R_t: 11.791 min (84% purity), LRMS *m/z* (ESI$^+$): 374.1 [M+2H] (100%), 746.9 [M+H] (90%), HRMS *m/z* (ESI$^+$): calc. for $C_{36}H_{60}N_9O_6S^+$ ([M+H$^+$]) 746.4382, found 746.4391.

a)

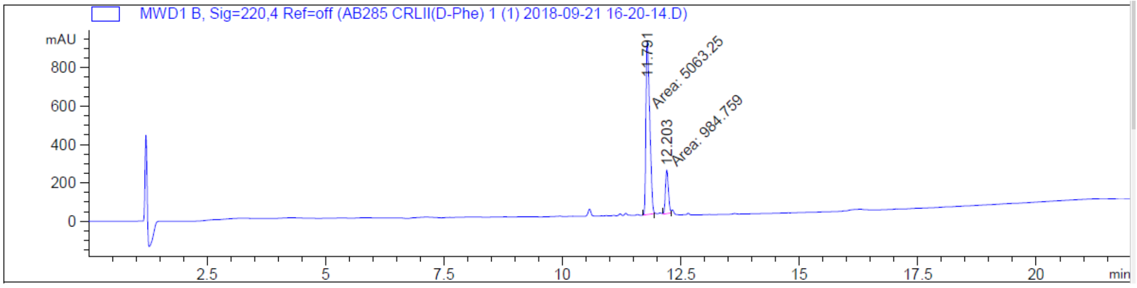

b)

Peak #	RetTime [min]	Type	Width [min]	Area [mAU*s]	Height [mAU]	Area %
1	11.791	MM	0.0927	5063.24609	909.88336	83.7176
2	12.203	MM	0.0716	984.75903	229.36151	16.2824

c)

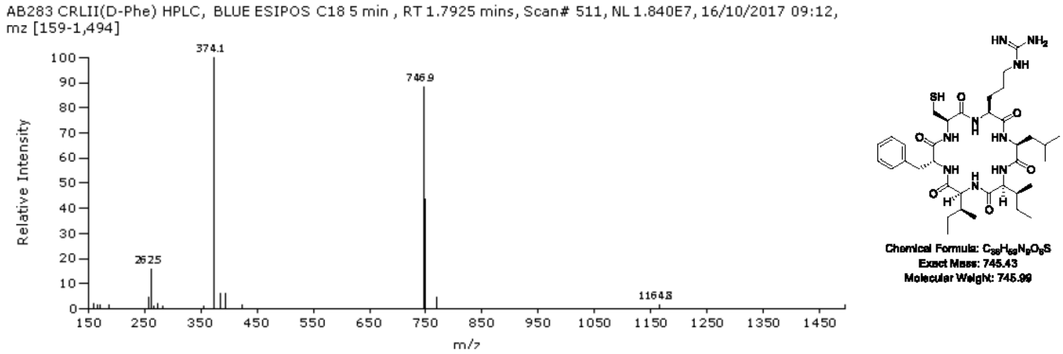

cyclo-CRLI(hL)F

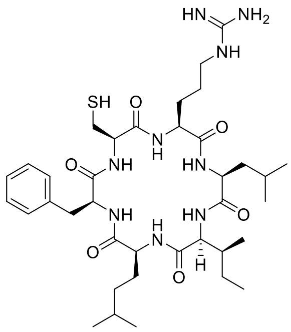

The linear peptide I(hL)FC(StBu)RL was synthesised from Fmoc-Leu-Wang resin (Novabiochem) on a 0.1 mmol scale following the general procedure above. After precipitation from ether, the peptide was dissolved in DMF and cyclised following the general procedure above. The peptide was concentrated, and then deprotected following the general procedure above. The product was purified by RP-HPLC and lyophilised to give a white solid (31 mg, 41% overall yield). LCMS R_t: 1.95 min, Analytical HPLC R_t: 13.394 min (99% purity), LRMS *m/z* (ESI$^+$): 381.2 [M+2H] (100%), 760.8 [M+H] (90%), HRMS *m/z* (ESI$^+$): calc. for $C_{37}H_{62}N_9O_6S^+$ ([M+H$^+$]) 760.4538, found 760.4548.

a)

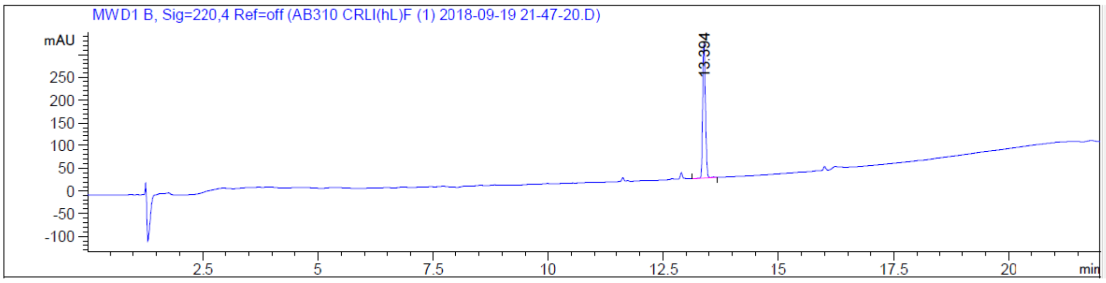

b)

Peak #	RetTime [min]	Type	Width [min]	Area [mAU*s]	Height [mAU]	Area %
1	13.394	BB	0.0911	1215.94128	277.60803	100.0000

c)

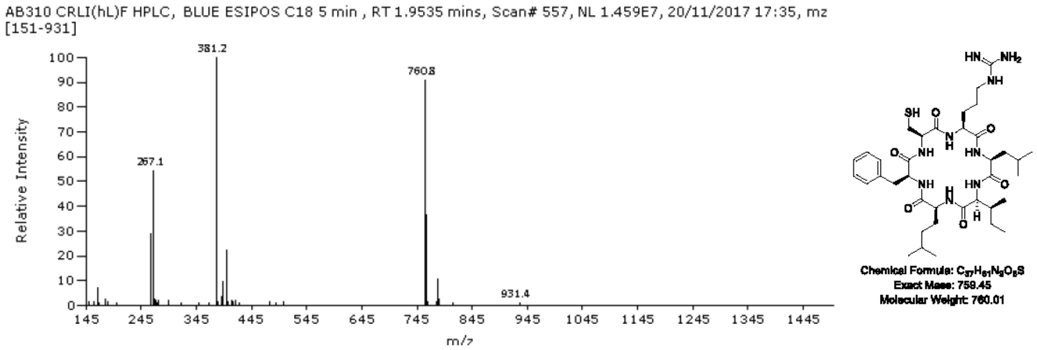

<u>*cyclo*-CRLI(Nle)F</u>

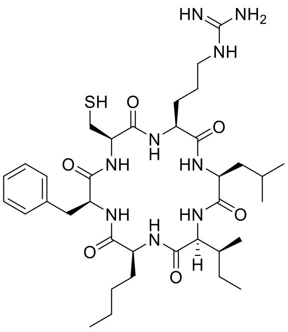

The linear peptide I(Nle)FC(StBu)RL was synthesised from Fmoc-Leu-Wang resin (Novabiochem) on a 0.1 mmol scale following the general procedure above. After precipitation from ether, the peptide was dissolved in DMF and cyclised following the general procedure above. The peptide was concentrated, and then deprotected following the general procedure above. The product was purified by RP-HPLC and lyophilised to give a white solid (44 mg, 59% overall yield). LCMS R_t: 1.87 min, Analytical HPLC R_t: 12.706 min (96% purity), LRMS *m/z* (ESI$^+$): 374.1 [M+2H] (100%), 746.8 [M+H] (90%), HRMS *m/z* (ESI$^+$): calc. for $C_{36}H_{60}N_9O_6S^+$ ([M+H$^+$]) 746.4382, found 746.4374.

a)

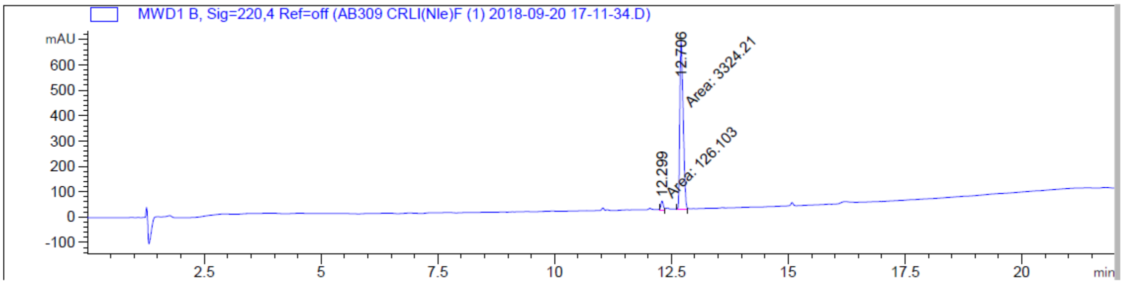

b)

Peak #	RetTime [min]	Type	Width [min]	Area [mAU*s]	Height [mAU]	Area %
1	12.299	MM	0.0595	126.10316	35.31187	3.6548
2	12.706	MM	0.0833	3324.20947	665.37469	96.3452

c)

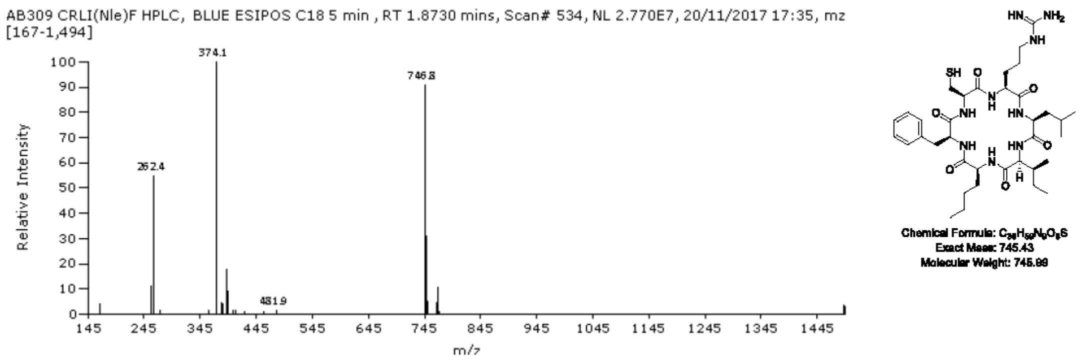

cyclo-CRLI(Nva)F

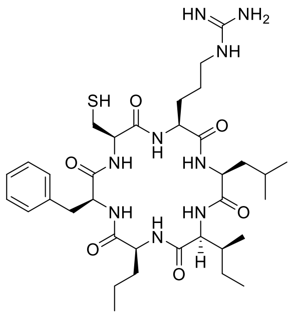

The linear peptide I(Nva)FC(StBu)RL was synthesised from Fmoc-Leu-Wang resin (Novabiochem) on a 0.1 mmol scale following the general procedure above. After precipitation from ether, the peptide was dissolved in DMF and cyclised following the general procedure above. The peptide was concentrated, and then deprotected following the general procedure above. The product was purified by RP-HPLC and lyophilised to give a white solid (32 mg, 44% overall yield). LCMS R_t: 1.77 min, Analytical HPLC R_t: 12.031 min (97% purity), LRMS *m/z* (ESI$^+$): 367.1 [M+2H] (100%), 732.7 [M+H] (75%), HRMS *m/z* (ESI$^+$): calc. for $C_{35}H_{58}N_9O_6S^+$ ([M+H$^+$]) 732.4225, found 732.4220.

a)

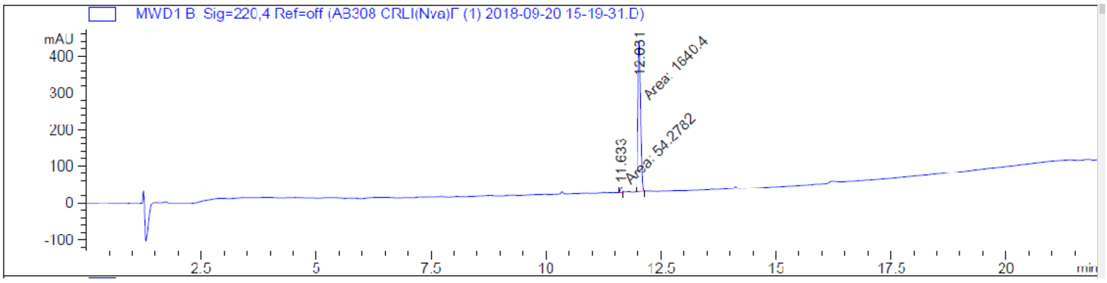

b)

Peak #	RetTime [min]	Type	Width [min]	Area [mAU*s]	Height [mAU]	Area %
1	11.633	MM	0.0556	54.27825	16.27815	3.2029
2	12.031	MM	0.0660	1640.40356	414.51065	96.7971

c)

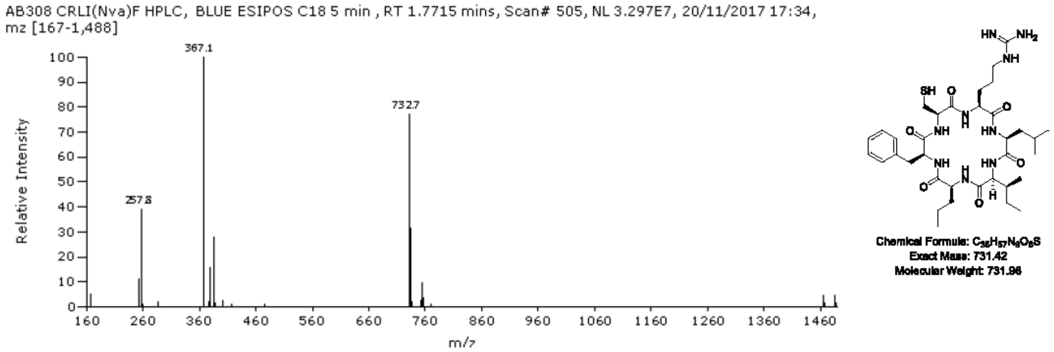

<u>*cyclo*-CRLILF</u>

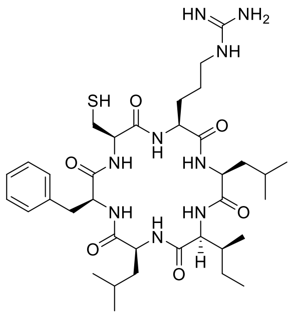

The linear peptide ILFC(StBu)RL was synthesised from Fmoc-Leu-Wang resin (Novabiochem) on a 0.1 mmol scale following the general procedure above. After precipitation from ether, the peptide was dissolved in DMF and cyclised following the general procedure above. The peptide was concentrated, and then deprotected following the general procedure above. The product was purified by RP-HPLC and lyophilised to give a white solid (43 mg, 57% overall yield). LCMS R_t: 1.87 min, Analytical HPLC R_t: 12.622 min (99% purity), LRMS *m/z* (ESI$^+$): 746.7 [M+H] (100%), 374.1 [M+2H] (85%), HRMS *m/z* (ESI$^+$): calc. for $C_{36}H_{60}N_9O_6S^+$ ([M+H$^+$]) 746.4382, found 746.4397.

a)

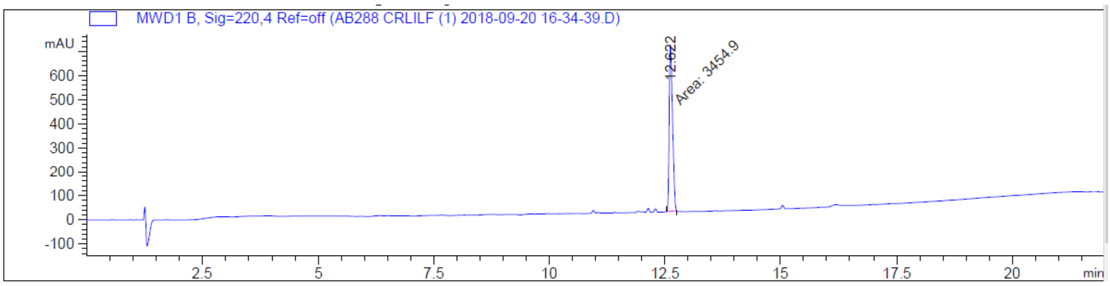

b)

Peak #	RetTime [min]	Type	Width [min]	Area [mAU*s]	Height [mAU]	Area %
1	12.622	MM	0.0828	3454.89697	695.15601	100.0000

c)

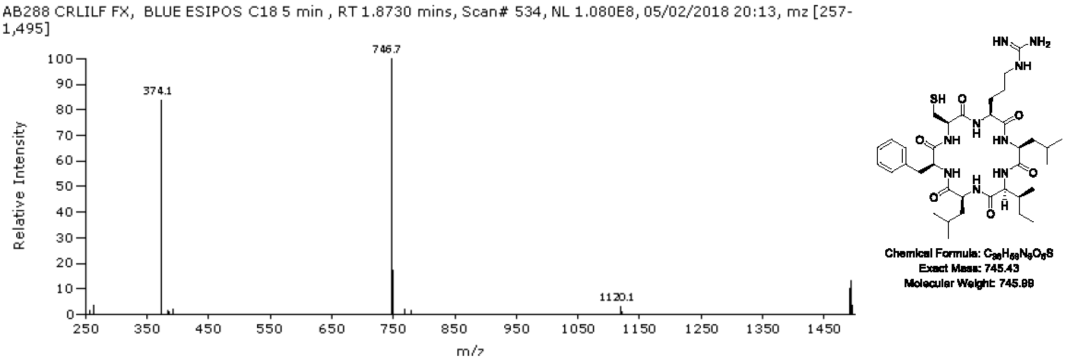

cyclo-CRLIVF

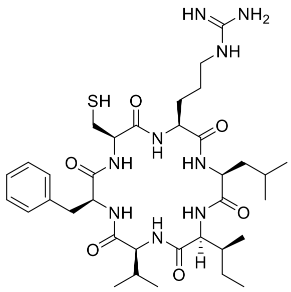

The linear peptide VFC(StBu)RLI was synthesised from Fmoc-Ile-Wang resin (Novabiochem) on a 0.1 mmol scale following the general procedure above. After precipitation from ether, the peptide was dissolved in DMF and cyclised following the general procedure above. The peptide was concentrated, and then deprotected following the general procedure above. The product was purified by RP-HPLC and lyophilised to give a white solid (15 mg, 21% overall yield). LCMS R_t: 1.79 min, Analytical HPLC R_t: 12.149 min (81% purity), LRMS *m/z* (ESI$^+$): 732.6 [M+H] (100%), 367.0 [M+2H] (50%), HRMS *m/z* (ESI$^+$): calc. for $C_{35}H_{58}N_9O_6S^+$ ([M+H$^+$]) 732.4225, found 732.4221.

a)

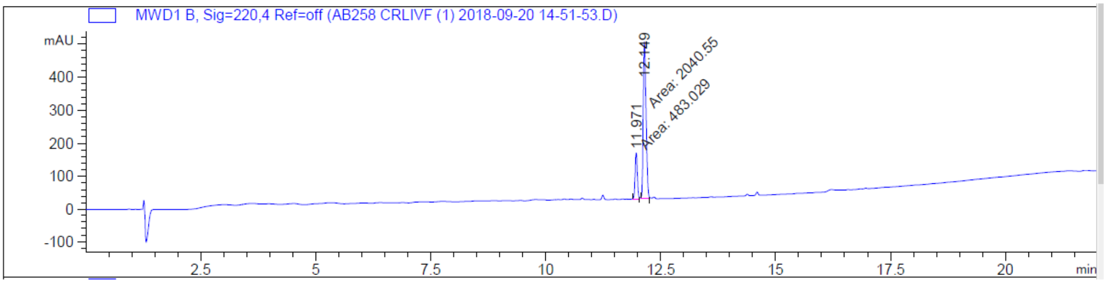

b)

Peak #	RetTime [min]	Type	Width [min]	Area [mAU*s]	Height [mAU]	Area %
1	11.971	MM	0.0567	483.02917	141.87651	19.1406
2	12.149	MM	0.0715	2040.55237	475.95917	80.8594

c)

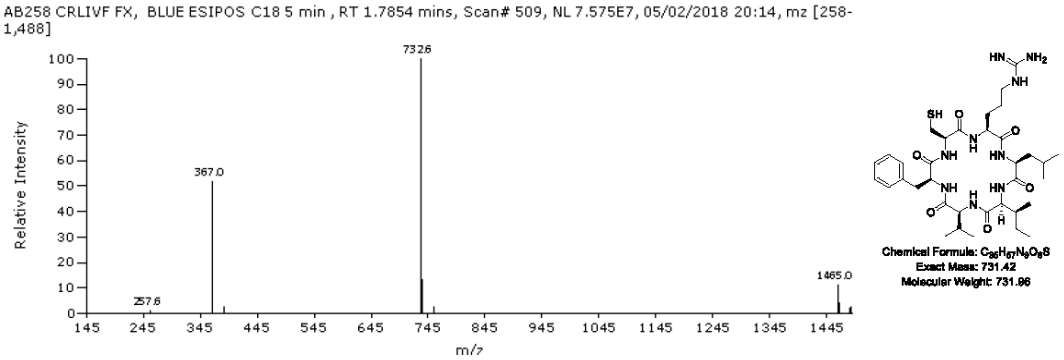

<u>*cyclo*-CRLI(Aib)F</u>

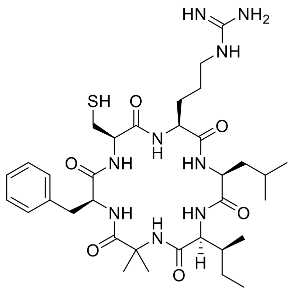

The linear peptide (Aib)FC(StBu)RLI was synthesised from Fmoc-Ile-Wang resin (Novabiochem) on a 0.1 mmol scale following the general procedure above. After precipitation from ether, the peptide was dissolved in DMF and cyclised following the general procedure above. The peptide was concentrated, and then deprotected following the general procedure above. The product was purified by RP-HPLC and lyophilised to give a white solid (18 mg, 25% overall yield). LCMS R_t: 1.63 min, Analytical HPLC R_t: 11.154 min (99% purity), LRMS *m/z* (ESI$^+$): 360.2 [M+2H] (100%), 718.8 [M+H] (70%), HRMS *m/z* (ESI$^+$): calc. for $C_{34}H_{56}N_9O_6S^+$ ([M+H$^+$]) 718.4069, found 732.4060.

a)

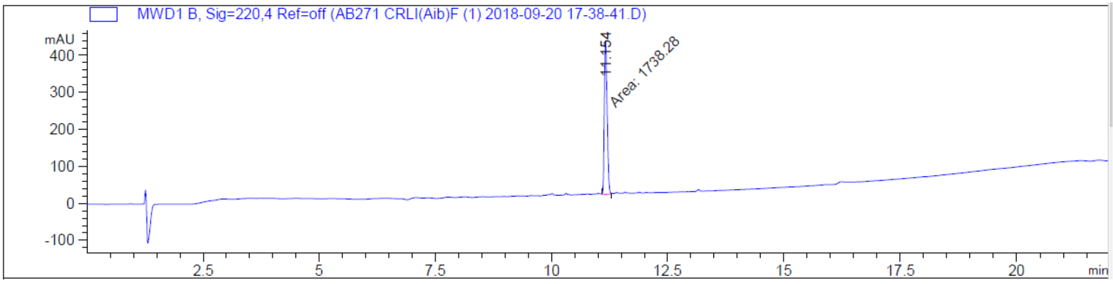

b)

Peak #	RetTime [min]	Type	Width [min]	Area [mAU*s]	Height [mAU]	Area %
1	11.154	MM	0.0695	1738.27832	416.67218	100.0000

c)

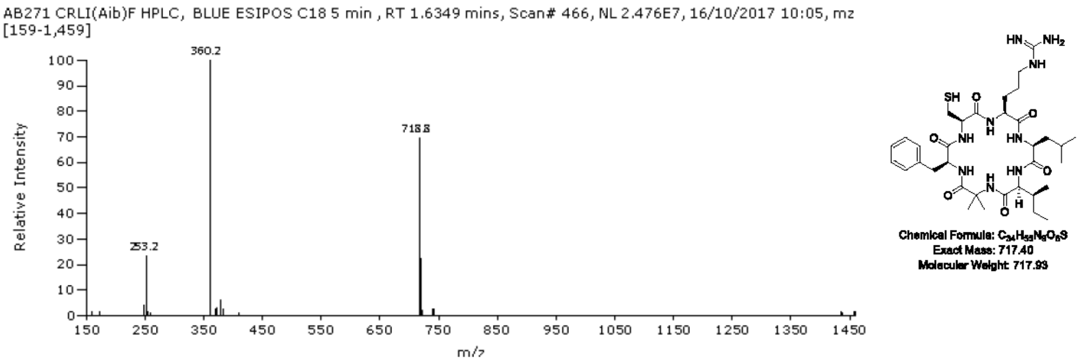

<u>*cyclo*-CRLI(Abu)F</u>

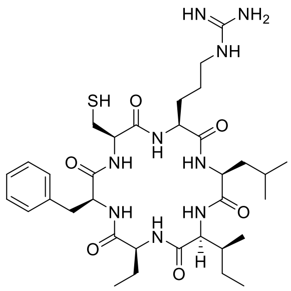

The linear peptide I(Abu)FC(StBu)RL was synthesised from Fmoc-Leu-Wang resin (Novabiochem) on a 0.1 mmol scale following the general procedure above. After precipitation from ether, the peptide was dissolved in DMF and cyclised following the general procedure above. The peptide was concentrated, and then deprotected following the general procedure above. The product was purified by RP-HPLC and lyophilised to give a white solid (48 mg, 67% overall yield). LCMS R_t: 1.69 min, Analytical HPLC R_t: 11.382 min (97% purity), LRMS *m/z* (ESI$^+$): 718.7 [M+H] (100%), 360.0 [M+2H] (80%), HRMS *m/z* (ESI$^+$): calc. for $C_{34}H_{56}N_9O_6S^+$ ([M+H$^+$]) 718.4069, found 718.4062.

a)

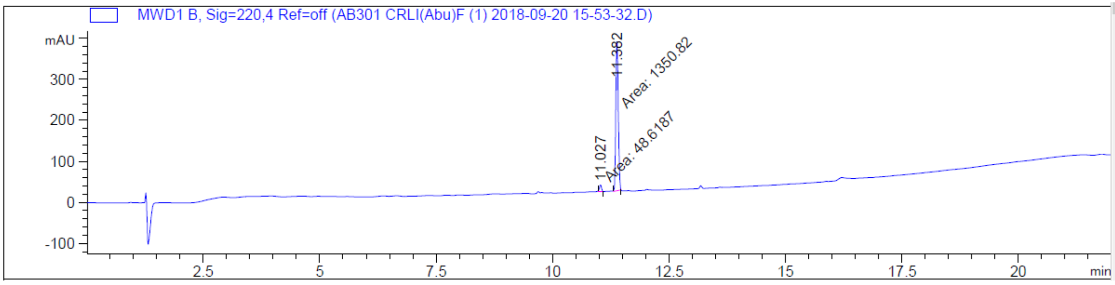

b)

Peak #	RetTime [min]	Type	Width [min]	Area [mAU*s]	Height [mAU]	Area %
1	11.027	MM	0.0509	48.61868	15.91651	3.4742
2	11.382	MM	0.0613	1350.82141	367.54324	96.5258

c)

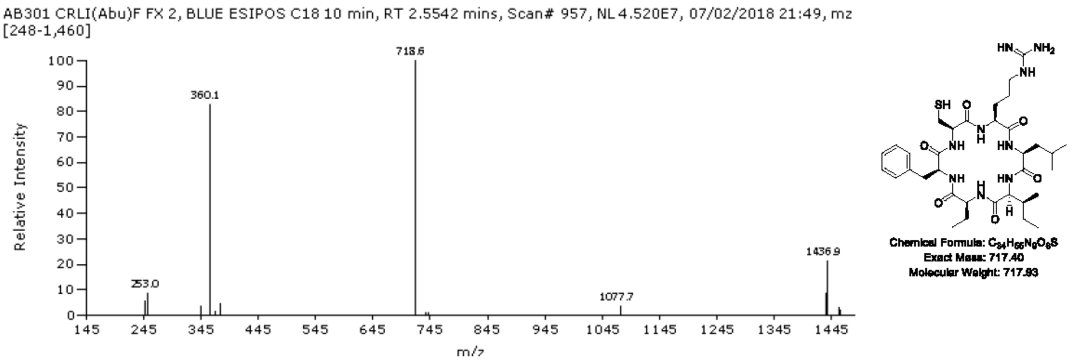

Ac-IFC-NH$_2$

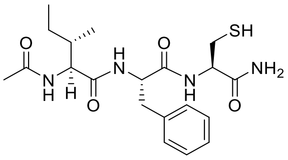

The peptide IFC(StBu) was synthesised from Fmoc-rink amide resin (Novabiochem) on a 0.2 mmol scale using SPPS coupling conditions described in section. The peptide, whilst still on-resin, was treated with a solution of Ac_2O (510 mg, 473 µL, 10 mmol, 25 eq.) and DIPEA (646 mg, 871 µL, 10 mmol, 25 eq.) in DMF (5 mL) and agitated with argon for 30 minutes. The resin was then washed three times with DMF, three times with DCM and three times with Et_2O and allowed to dry. Capping of the N-terminus was confirmed by Kaiser test. The peptide was then cleaved from resin using a TFA/TIS/H_2O cleavage cocktail and precipitated from ether as described in section. The peptide was then dissolved in DMF and deprotected following the general procedure above. The product was purified by RP-HPLC and lyophilised to give a white solid (29 mg, 34% overall yield). LCMS R_t: 1.90 min, Analytical HPLC R_t: 8.848 min (82% purity), LRMS *m/z* (ESI$^+$): 423.4 [M+H] (100%), 445.3 [M+Na] (65%), HRMS *m/z* (ESI$^+$): calc. for $C_{20}H_{30}N_4O_4SNa^+$ ([M+Na$^+$]) 445.1880, found 445.1886.

a)

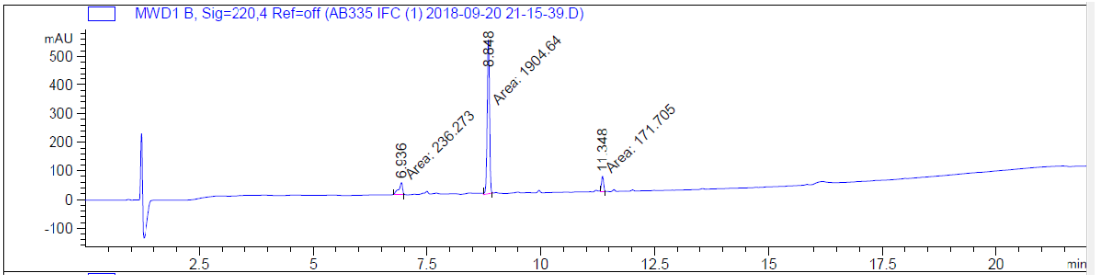

b)

Peak #	RetTime [min]	Type	Width [min]	Area [mAU*s]	Height [mAU]	Area %
1	6.936	MM	0.0949	236.27269	41.48911	10.2167
2	8.848	MM	0.0584	1904.63574	543.88672	82.3586
3	11.348	MM	0.0533	171.70496	53.64857	7.4247

c)

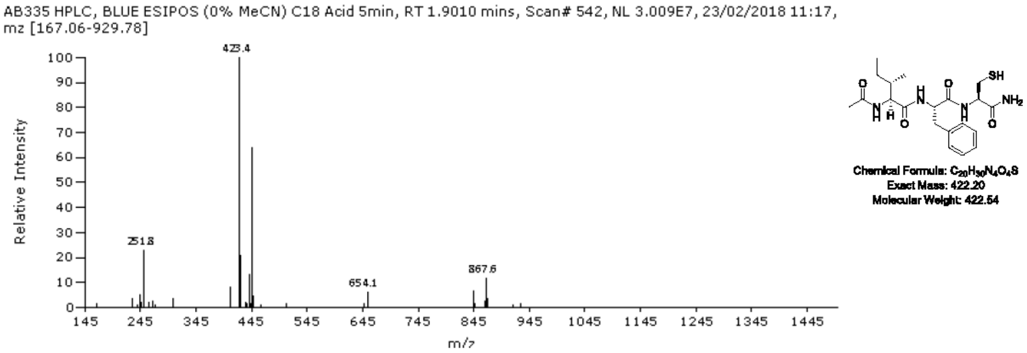

<u>Ac-IF(4-I)C-NH2</u>

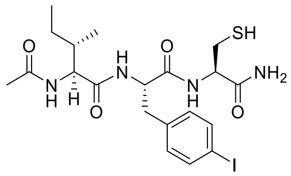

The peptide IFC(StBu) was synthesised from Fmoc-rink amide resin (Novabiochem) on a 0.2 mmol scale using SPPS coupling conditions described in section. The peptide, whilst still on-resin, was treated with a solution of Ac_2O (510 mg, 473 µL, 10 mmol, 25 eq.) and DIPEA (646 mg, 871 µL, 10 mmol, 25 eq.) in DMF (5 mL) and agitated with argon for 30 minutes. The resin was then washed three times with DMF, three times with DCM and three times with Et_2O and allowed to dry. Capping of the N-terminus was confirmed by Kaiser test. The peptide was then cleaved from resin using a TFA/TIS/H_2O cleavage cocktail and precipitated from ether as described in section. The peptide was then dissolved in DMF and deprotected following the general procedure above. The product was purified by RP-HPLC and lyophilised to give a white solid (5 mg, 9% overall yield). LCMS R_t: 1.68 min, Analytical HPLC R_t: 11.062 min (86% purity), LRMS *m/z* (ESI$^+$): 571.3 [M+Na] (100%), 549.3 [M+H] (75%), HRMS *m/z* (ESI$^+$): calc. for $C_{20}H_{29}IN_4O_4SNa^+$ ([M+Na$^+$]) 571.0846, found 571.0832.

a)

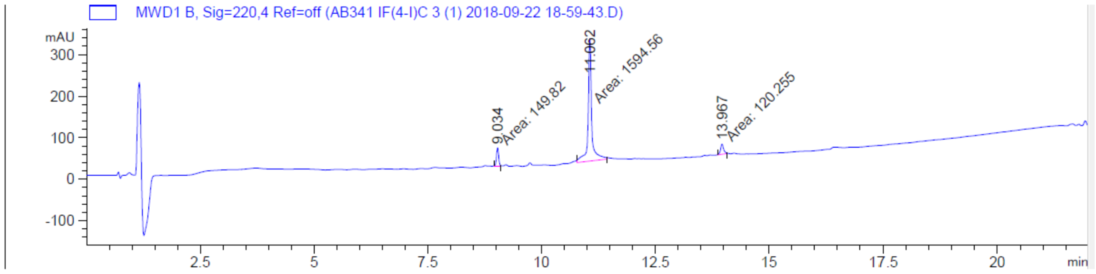

b)

Peak #	RetTime [min]	Type	Width [min]	Area [mAU*s]	Height [mAU]	Area %
1	9.034	MM	0.0558	149.81992	44.71027	8.0348
2	11.062	MM	0.0898	1594.56079	295.90875	85.5160
3	13.967	MM	0.0806	120.25474	24.85474	6.4492

c)

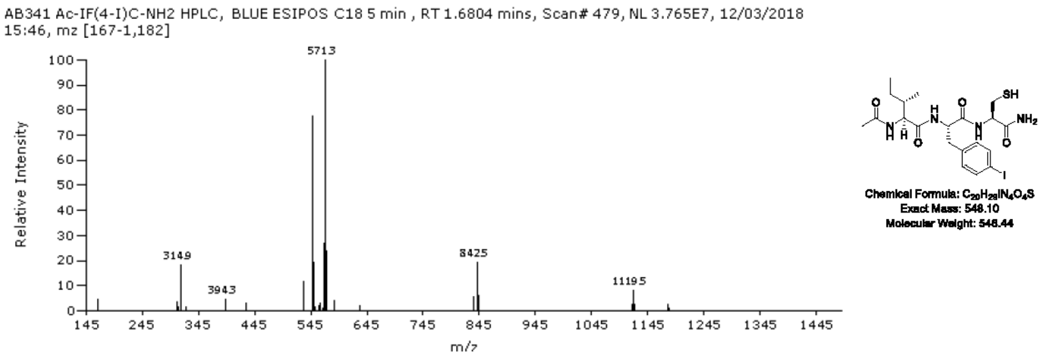

References

1. Maier JA, Martinez C, Kasavajhala K, Wickstrom L, Hauser KE, Simmerling C. ff14SB: Improving the Accuracy of Protein Side Chain and Backbone Parameters from ff99SB. Journal of Chemical Theory and Computation. 2015;11(8):3696-713 doi: 10.1021/acs.jctc.5b00255.
2. Jorgensen WL, Chandrasekhar J, Madura JD, Impey RW, Klein ML. Comparison of simple potential functions for simulating liquid water. The Journal of Chemical Physics. 1983;79(2):926-35 doi: 10.1063/1.445869.
3. Salt DW, Hudson BD, Banting L, Ellis MJ, Ford MG. DASH: a novel analysis method for molecular dynamics simulation data. Analysis of ligands of PPAR-gamma. J Med Chem. 2005;48(9):3214-20 doi: 10.1021/jm049216s.
4. Bussi G, Donadio D, Parrinello M. Canonical sampling through velocity rescaling. The Journal of Chemical Physics. 2007;126(1):014101 doi: 10.1063/1.2408420.
5. Parrinello M, Rahman A. Polymorphic transitions in single crystals: A new molecular dynamics method. Journal of Applied Physics. 1981;52(12):7182-90 doi: 10.1063/1.328693.
6. van Zundert GCP, Rodrigues JPGLM, Trellet M, Schmitz C, Kastritis PL, Karaca E, et al. The HADDOCK2.2 Web Server: User-Friendly Integrative Modeling of Biomolecular Complexes. Journal of Molecular Biology. 2016;428(4):720-5 doi: https://doi.org/10.1016/j.jmb.2015.09.014.
7. Bhardwaj G, Mulligan VK, Bahl CD, Gilmore JM, Harvey PJ, Cheneval O, et al. Accurate de novo design of hyperstable constrained peptides. Nature. 2016;538(7625):329-35 doi: 10.1038/nature19791.
8. Mirdita M, Schütze K, Moriwaki Y, Heo L, Ovchinnikov S, Steinegger M. ColabFold: making protein folding accessible to all. Nature Methods. 2022;19(6):679-82 doi: 10.1038/s41592-022-01488-1.
9. Jumper J, Evans R, Pritzel A, Green T, Figurnov M, Ronneberger O, et al. Highly accurate protein structure prediction with AlphaFold. Nature. 2021;596(7873):583-9 doi: 10.1038/s41586-021-03819-2.
10. Wu D, Potluri N, Lu J, Kim Y, Rastinejad F. Structural integration in hypoxia-inducible factors. Nature. 2015;524(7565):303-8 doi: 10.1038/nature14883.
